# Consumer beliefs about healthy foods and diets

**DOI:** 10.1371/journal.pone.0223098

**Published:** 2019-10-15

**Authors:** Jayson L. Lusk

**Affiliations:** Department of Agricultural Economics, Purdue University, West Lafayette, Indiana, United States of America; SOAS, University of London, UNITED KINGDOM

## Abstract

**Background:**

The U.S. Food and Drug Administration has begun a public process to redefine how companies are allowed to use the term “healthy” on food packages. Although the definition is formulated based on the latest consensus in nutrition and epidemiological research, it is also important to understand how consumers define and understand the term if it is to be behaviorally relevant. This research is an exploratory study designed to provide a descriptive account of consumers’ perceptions of and beliefs about the meaning of “healthy” food.

**Methods:**

A nationwide U.S. sample of 1,290 food consumers was surveyed in December 2018. Respondents answered 15 questions designed to gauge perceptions of healthy food and to elicit preference for policies surrounding healthy food definitions. Responses are weighted to demographically match the population. Categorical variables have a sampling error of ±2.7%. Exploratory factor analysis is used to determine latent dimensions of health perceptions related to food type.

**Results:**

Consumers were about evenly split on whether a food can be deemed healthy based solely on the foods’ nutritional content (52.1% believing as such) or whether there were other factors that affect whether a food is healthy (47.9% believing as such). Consumers were also about evenly split on whether an individual food can be considered healthy (believed by 47.9%) or whether this healthiness is instead a characteristic of one’s overall diet (believed by 52.1%). Ratings of individual food products revealed that “healthy” perceptions are comprised of at least three underlying latent dimensions related to animal origin, preservation, and freshness/processing. Focusing on individual macronutrients, perceived healthiness was generally decreasing in a food’s fat, sodium, and carbohydrate content and increasing in protein content. About 40% of consumers thought a healthy label implied they should increase consumption of the type of food bearing the label and about 15% thought the label meant they could eat all they wanted.

**Conclusions:**

Results suggest consumer’s perceptions of “healthy,” which is primarily based on fat content, partially aligns with the FDA definition but also suggest consumers perceive the word as a broader and more nuanced concept that defies easy, uniform definition. Results highlight areas where nutrition education may be needed and suggest disclosures may need to accompany health claims so that consumers know what, precisely, is being communicated.

## Background

Food labels and claims presumably enable consumers to make more informed food choices [1]. However, creating and regulating labels is costly, and labels can sometimes mislead rather than inform [2,3]. These competing considerations suggest the need to evaluate the costs and benefits of changes in labeling policy.

There is a large academic literature investigating the impact of various nutrition-related labels on consumer choice, e.g., see reviews in [4, 5, 6]. Examples include impacts of calorie labels on restaurant menus [7, 8, 9], nutrition facts panels [10, 11], front-of-pack nutrition labeling [12, 13], traffic light, numeric, and symbolic nutrition labels [14, 15, 16, 17, 18], exercise-equivalent calorie labels [19], and more. There have also been a number of studies focused on consumers’ reactions to specific nutrition-related claims such as “light” [20], “low fat” [21], “low calorie” [22], “less sugar” [23] or health-related claims such as “lowers cholesterol” [24]. Many of these studies show that these labels and claims have small or sometimes counter-intuitive effects, in part because such labels may send other signals about taste, convenience, or affordability [25].

Despite this large body of literature focused on health- and nutrition-related labels and claims, much less is known about how consumers perceive the concept of “healthy” in and of itself. One of the few studies on the subject [26], found, in a simulated supermarket shopping experiment with Dutch consumers, that a “healthy choice” label had an impact statistically indistinguishable from a “special offer” label, but this study did not explore consumers perceptions of the term. While some nutritional information is purely “objective” and scientific in nature (e.g., the Nutrition Facts Panel), there are some unregulated claims that companies use for marketing purposes (e.g., the use of the word “natural” on non-meat food items). “Healthy” claims lie somewhere in this spectrum. Use of the term is regulated by the Food and Drug Administration (FDA) and current science, as outlined for example in the Dietary Guidelines for Americans, serves as the basis for the legal definition. But, of course, consumer’s perceptions of the term do not necessarily align with the scientific and legal definition.

“Healthy” has been defined by the FDA since 1993 with primary reference to total fat content, with changes made in 2016 to discriminate between different types of fat [27–29]. To meet the conditions allowing a “healthy” claim today, foods must generally be low in total fat, low in saturated fat, meet certain cholesterol specifications, and provide at least 10% of the recommended daily intake of certain vitamins or minerals [29]. The exact requirements for the claim depend on the type of food as outlined in [27]. Generally, low fat implies the food, “contains 3 g or less of fat per reference amount customarily consumed” [29]. Low in saturated fat generally implies, “The food contains 1 g or less of saturated fatty acids per reference amount customarily consumed and not more than 15 percent of calories from saturated fatty acids” [29]. Depending on the type of food, a food labeled “healthy” must contain at least 10% of the recommended daily amounts of one or more of the following: vitamin A, vitamin C, calcium, iron, protein or fiber [27].

At the time of the initial implementation, American Dietetic Association argued that there were no good or bad foods, without consideration of one’s overall diet; they also went on to define criteria all types of food should meet to attain a healthy label [30]. Recently, however, the FDA began a process to re-define the term. In their public request for comments [28], the FDA echoed some of these previous debates and asked for feedback on questions like, “Is the term ‘healthy’ most appropriately categorized as a claim based only on nutrient content?” The FDA also asked, “What is consumer’s understanding of the meaning of the term “healthy” as it relates to food? What are consumers’ expectations of foods that carry a “healthy” claim? We are especially interested in any data or other information that evaluates whether or not consumers associate, confuse, or compare the term “healthy” with other descriptive terms and claims” [28]. The purpose of this study is to provide such information.

It is clear that consumers are willing to pay more for products they perceive as healthy, e.g., [15], but more fundamental information is needed on the determinants of health perceptions–i.e., what foods, ingredients, and processes consumers perceive as healthy. Federal definitions behind health claims are based on current thinking in nutritional and epidemiological science, but it is possible that consumer beliefs might diverge from that of scientists if there is a lag between the evolution of scientific knowledge and consumer perceptions or if consumers distrust scientific consensus. In cases where consumer perceptions diverge from latest scientific thinking, there are potential opportunities for nutritional education to narrow the gap. To the extent there is a divergence in perceptions and claim definitions, possibilities for confusion and mis-understanding exist, which might suggest the need for additional disclosures on food packages to clarify the definition of labeling claims. Finally, identification of gaps between lay- and scientific-perceptions and understanding can lead to the development of models to help understand how such gaps arise and what to do about them [31–33]. This research is an exploratory study designed to provide a descriptive account of consumers’ perceptions of and beliefs about the meaning of “healthy” food.

## Methods

### Participants

A nationwide, online survey of U.S. food consumers was fielded from December 11^th^ to the 17^th^, 2018. The survey was written and programmed by the author and was administered to an online panel maintained by Survey Sampling International. Purdue University’s Human Research Protection Program determined that the research project qualifies as exempt from IRB review under U.S. federal human subjects research regulations. A total of 2,306 responses were obtained.

The first question asked how much of the grocery shopping the respondent did for their household. Anyone who provided an answer indicating that they were responsible for less than half their household’s grocery shopping was immediately directed to the end of the survey and were excluded from this analysis (excluding 322 respondents). Additional exclusionary criteria entailed removal of respondents who failed checks for data quality control. Two “trap” questions were included, which asked respondents to choose a specific answer (e.g., “somewhat agree”) if they were paying attention (see [34], [35] for further discussion on use of trap questions to improve quality of survey responses). Respondents who missed either of the trap questions were also excluded from the analysis. Lastly, as a further quality control measure, responses to open-ended questions were inspected, and respondents who provided nonsensical answers (e.g., “asdkf”) were removed from the sample. After applying the aforementioned exclusionary criteria, the final sample consists of 1,290 respondents. Demographic characteristics of the respondents are presented in Table A in S1 Appendix. This paper reports the results from a survey which included multiple objectives. A document, which shows all questions asked and the top-line results, as well as the entire dataset and the associated codebook are available at: https://doi.org/10.7910/DVN/IEHI3U.

### Study design

This paper focuses on a set of questions aimed at measuring perceptions of “healthy.” A number of measures were used to judge consumers’ perceptions of healthy, which focused on 1) the concept of healthy as a whole, 2) particular ingredients, 3) particular foods, and 4) particular nutrients. The order of these questions was randomized across respondents.

Healthy as a meta concept was explored by asking two questions, both of which pitted two competing views; respondents were asked, “Which of the following best matches your view?” The first focused on whether healthiness relates to more than nutrient content. The two options, presented in random order were: A) If I know the nutrient content of the food (the amount of fat, protein, carbs, vitamins, etc.), I know enough to decide whether a food is healthy, or B) I need to know more than just the nutrient content (the amount of fat, protein, carbs, vitamins, etc.) of a food to decide whether it is healthy. The second question aimed to identify whether healthiness is a property of particular foods or the overall diet. The two options, presented in random order, were: A) It is better to think about “healthy” on a food-by-food basis (some foods are healthy and some aren’t) or “It is better to think about “healthy” by looking at a whole dietary pattern (healthiness is defined by combinations of foods in a diet).

Healthy perceptions as related to particular foods was gauged by asking, “Do you consider each of the following foods to be healthy or unhealthy for you?” Fifteen different foods were listed, and for each, respondents indicated one of three categories: healthy, neither healthy nor unhealthy, or unhealthy. The food items were selected to represent broad food categories that were easily recognizable by consumers. In general, food items were selected to be consistent with the categories employed in the widely-studied Bureau of Labor Statistics Consumer Expenditure Survey, which collects and reports household spending in 5 broad food categories and 19 sub-categories. Attention was focused on those sub-categories where consumers spend the most money: cereal and bakery products, meats (including beef, pork, poultry, fish, and eggs), dairy products, fresh and processed fruits and vegetables, and “other food at home” (the largest expense sub-categories of which are “sugar and other sweets” and “fats and oils”). “Candy” was the food used to represent the “sugar and other sweets” category. For each item, a healthiness score was created by subtracting the percent of respondents who considered a process unhealthy from the percent of respondents who considered a process healthy.

Health perceptions as related to ingredients was ascertained by asking, “Which of the following affects whether or not you would consider a food healthy for you? (check up to 3 items that most apply).” Thirteen ingredients/attributes were listed including sugar content, fat content, use of GMOs, use of preservatives, etc. Rather than asking “choose all that apply”, respondents were asked to choose a limited number, i.e., “choose the three items that most apply” so as to force respondents to prioritize their responses and to induce more careful consideration.

Health perceptions related to nutrients was measured by asking, “Which of the following do you consider to be the most healthy for you?” for four different macro nutrients: sodium, carbohydrates, fat, and protein. For each nutrient, there were five response categories ranging from low, medium low, medium, medium high, and high.

In addition to measuring health perceptions, the study elicited behavioral implications of healthy labels. Respondents were asked, “If a food is labeled “healthy”, what would that mean to you? (check up to 3 items that most apply).” Six different options, in random order, were presented to respondents. Respondents preferences of how the FDA should define healthy was elicited with the following question: “Currently, the Food and Drug Administration (FDA) allows the label "healthy" to be used on foods low in fat and saturated fat and that provide at least 10% of recommended amounts of vitamin A, vitamin C, calcium, iron, protein, or fiber. How do you believe healthy labels should be regulated in the future?” There were four mutually exclusive response categories: A) The FDA should regulate to prevent the use of the term “healthy” on food packages, B) The FDA should regulate the use of the term “healthy” by requiring companies follow a uniform, consistent definition, C) The FDA should not regulate the use of the term “healthy” on food packages, and D) The FDA should keep the current definition of “healthy.”

Finally, respondents a number of questions eliciting socio-economic and demographic characteristics.

### Statistical analysis

Weights were created that, when applied to calculations of means and proportions, force the sample to match the U.S. population in terms of region of residence in the U.S., age, education, and gender. For categorical variables, the study sample size yields a sampling error of 2.7% for outcome variables of interest. For questions related to perceived healthiness of different foods, exploratory factor analysis was conducted. Factor analysis groups variables (or in this case types of foods) according to underlying latent or “hidden” dimensions. Thus, factor analysis enables one to uncover the dimensions or factors that people use to define healthiness beyond just the type of food without necessarily having to know ex ante how consumers may group and conceptualize the healthfulness of different foods.

## Results

Table 1 shows consumers were about evenly split on whether a food can be deemed healthy based solely on the foods’ nutritional content (52.1% believing as such) or whether there were other factors that affect whether a food is healthy (47.9% believing as such). Consumers were also evenly split on whether an individual food can be considered healthy (believed by 47.9%) or whether this healthiness is instead a characteristic of one’s overall diet and the combination of foods consumed (believed by 52.1%). Responses to these two questions are not determinative of each other, but rather there are four distinct consumer segments with regard to healthy food conceptions as illustrated in the cross tab in Table 1.

**Table 1:** Percent of respondents with four different views on how healthy should be defined.

	Single foods can be considered healthy or unhealthy	Only a whole diet can be considered healthy or unhealthy	Total
Healthiness based on nutrients alone	26.5% (Food-Nutrient Focus: health based on a food’s nutrients)	25.6% (Diet-Nutrient Focus: health based on nutrients from whole diet)	52.1%
Healthiness based on more than nutrient content	21.4% (Food-Nonnutritive Focus: health based on a food’s entire composition)	26.5% (Diet- Nonnutritive Focus: health based on holistic consumption pattern)	47.9%
Total	47.9%	52.1%	

Note: sampling error for each statistic is ±2.7%.

Given responses were about evenly divided among all four response categories, it is possible that outcomes may reflect random response patterns. One way to attempt to disentangle random responses from truly divided opinions is to explore whether response patterns correlate with demographic variables, which would suggest more systematic drivers of choice. A multinomial logit model constructed to explain responses falling into one of the four categories in Table 1, in fact, results reveal several demographic variables are predictive of response patters. For example, men, less educated, and older consumers are significantly more likely to fall in the joint category (healthiness based on nutrients alone, single foods can be considered healthy or unhealthy) than women, more educated, younger consumers.

**Fig 1:**
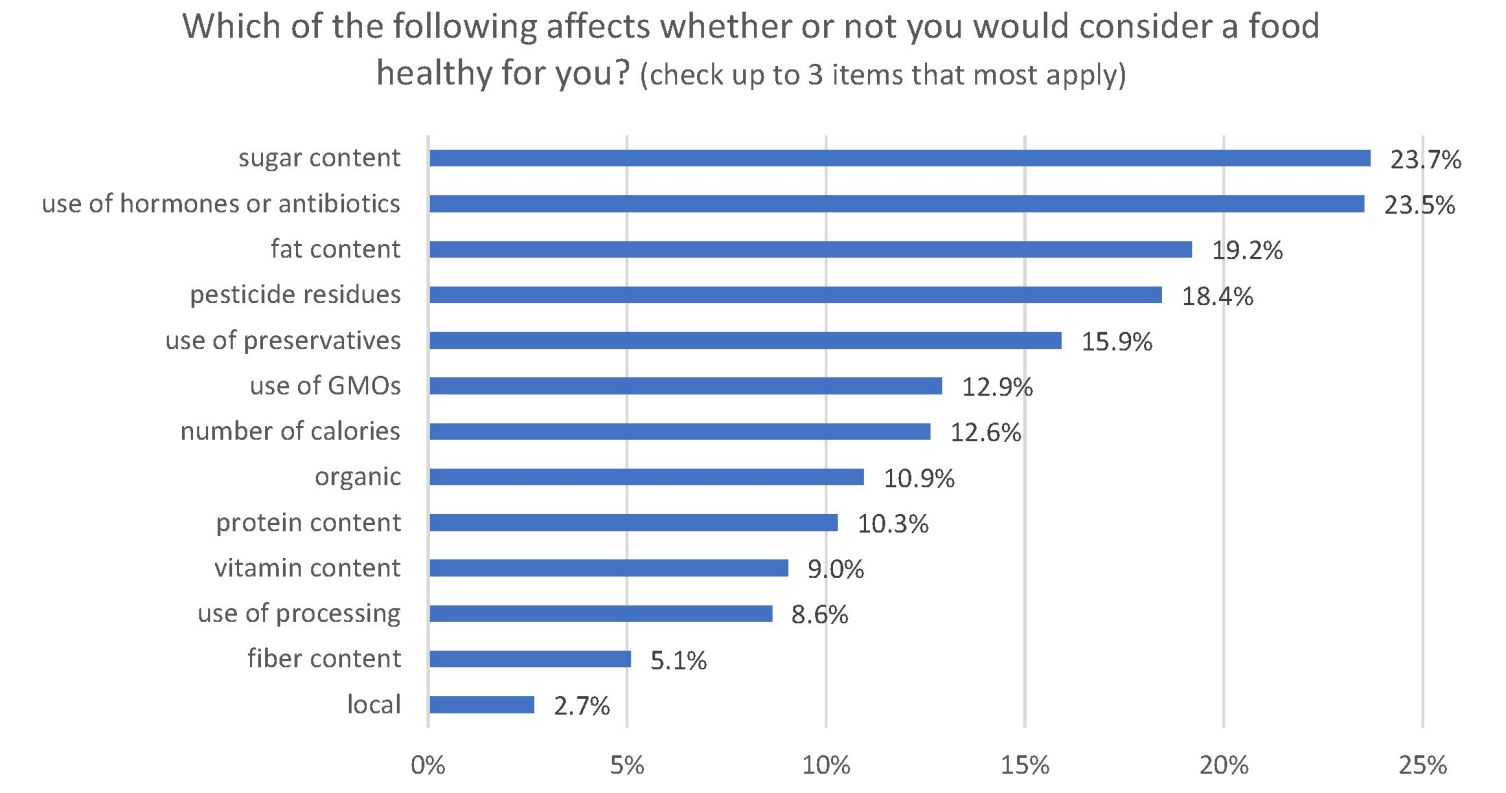
Factors affection consumers’ perceptions of a food’s healthiness (*sampling error for each statistic is ±2*.*7%*.*)*.

Focusing on ingredient-related healthy definition, respondents were provided with a list of 13 factors that consumers might use to judge whether a food is healthy. Fig 1 shows that about a quarter of respondents indicated sugar content and use of hormones or antibiotics as, 19.2% pointed to fat content, and 18.4% pointed to pesticide residue, as ingredients/attributes that are most likely to influence healthy perceptions of a food. The top four answers included two nutrients (sugar and fat) and two food production processes/ingredients (hormones and pesticides), suggesting consumers consider healthiness to be more than just defined by nutrient content. Use of GMOs was considered as a factor affecting healthiness at about the same rate as caloric content. The least frequently picked items were processing, fiber content, and local.

**Fig 2:**
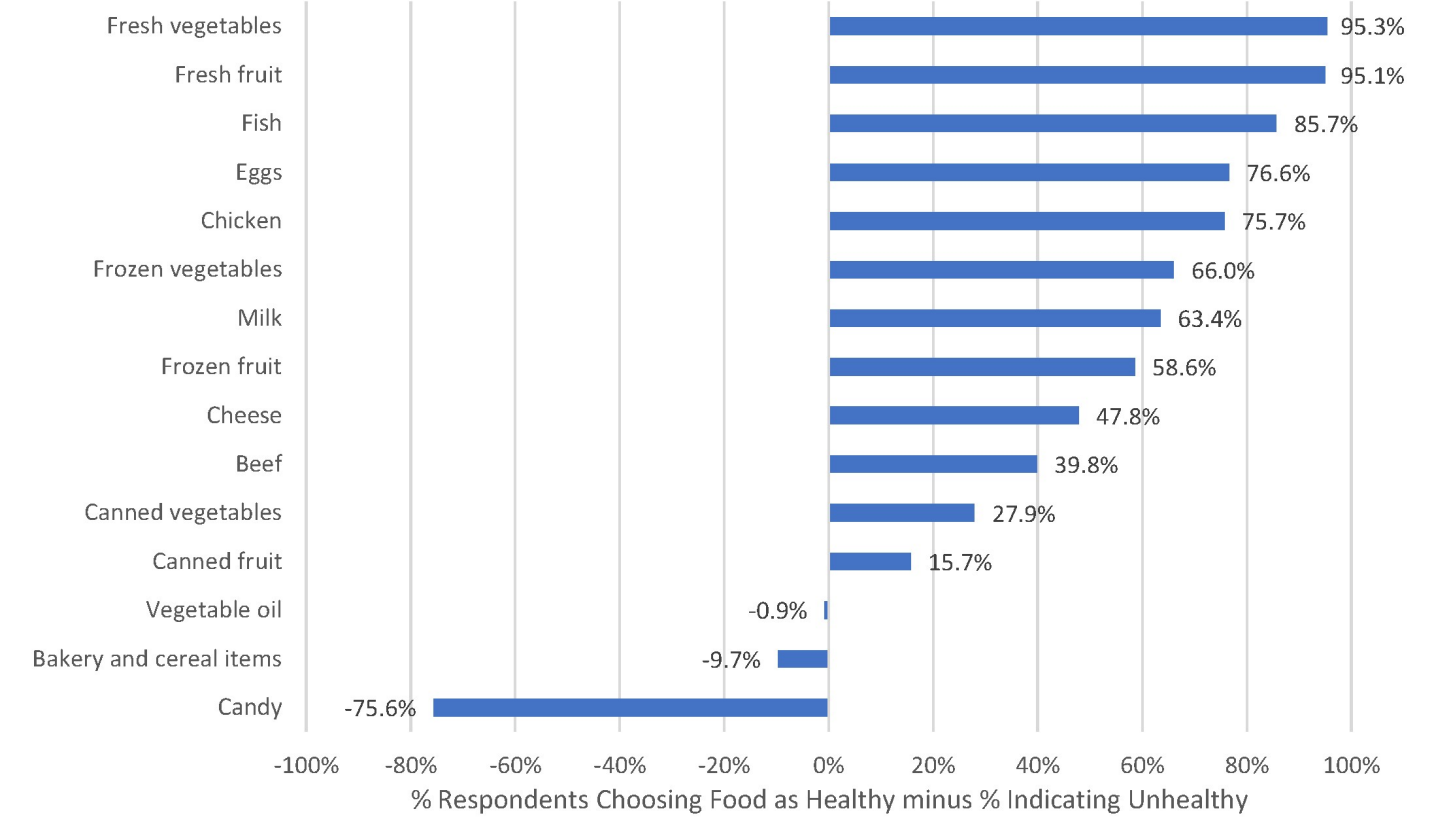
Perceived healthiness of 15 Foods.

Respondents’ healthy perceptions as they related to particular foods is shown in Fig 2. Almost all respondents (96.2%) considered fresh vegetables to be healthy, and almost none (0.9%) considered them unhealthy, yielding a net healthy score of 96.2–0.9 = 95.3% for fresh vegetables. Fresh fruit, fish, eggs, and chicken were likewise broadly considered healthier than not. Frozen vegetables/fruit were considered less healthy than fresh, and canned were considered less healthy than frozen, although even canned was considered, on net, more healthy than unhealthy. Only three of the 15 items listed were considered by more respondents to be unhealthy than healthy: vegetable oil, bakery and cereal items, and particularly candy. A third of respondents thought bakery and cereal items were unhealthy, but 23.3% thought such items were healthy, and 43.7% thought such items were neither healthy nor unhealthy. 49% (the highest for any food considered) said vegetable oil was neither healthy nor unhealthy. Candy was the only item a plurality of respondents thought was unhealthy.

**Fig 3:**
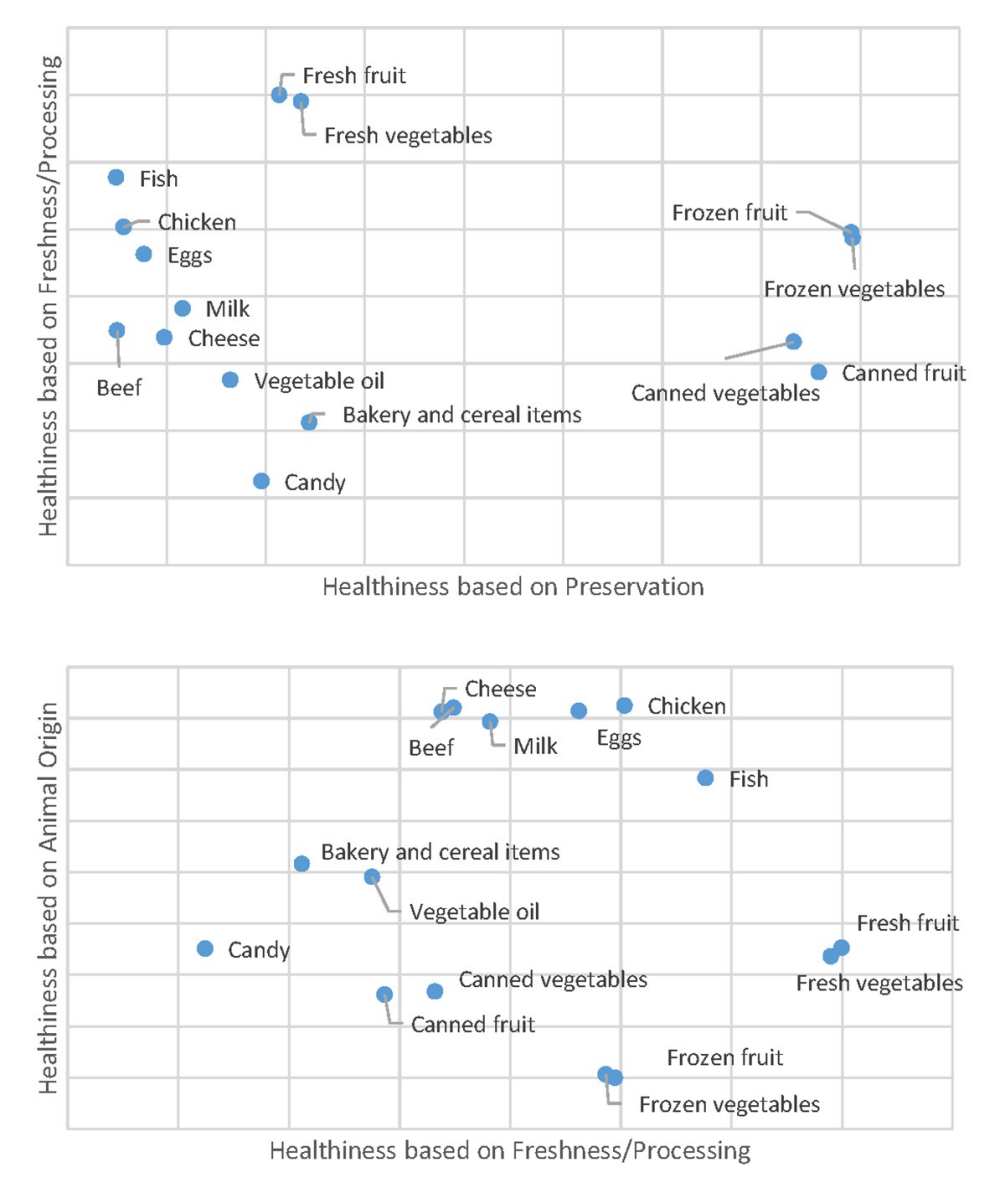
Three dimensions of 15 food’s healthiness (Factor loadings from promax rotation).

To explore how consumers conceptualized the healthiness of different foods, the questions used to create Fig 2 were further analyzed using exploratory factor analysis. Analysis reveals that there are three underlying factors that explain 54%, 31%, and 22% of the variation in whether consumers rate a food as healthy or not (see Tables B and C in S1 Appendix for additional inputs to and outputs of the factor analysis). Fig 3 plots the 15 foods according to their factor loadings (standardized regression coefficients) from promax rotation. The first factor (explaining 54% of the total variance), shown on the vertical axis of the bottom panel of Fig 3 shows all animal products with high values and other non-animal products with lower values, suggesting consumers use animal origin as a primary factor in judging whether a food is healthy. A second factor (explaining 31% of the total variance), illustrated on the horizontal axis of the top panel of Fig 3, has canned and frozen fruits and vegetables with the highest values, bakery and cereal items, candy, and fresh fruits and vegetables with mid-to-low values, and animal products with the lowest values, which seems to suggest consumers use degree of preservation as another dimension of healthiness. Finally, the third factor (explaining 22% of total variance), illustrated on the vertical axis of the top panel and the horizontal axis of the bottom panel of Fig 3, indicates freshness or degree of processing is another dimension to healthiness evaluations. These results indicate that healthiness is not a single unifying construct, but rather consumers evaluate healthiness along a number of different dimensions or factors. A food, such as beef or fish, can be seen as scoring high in some dimensions of healthy but low in another.

**Fig 4:**
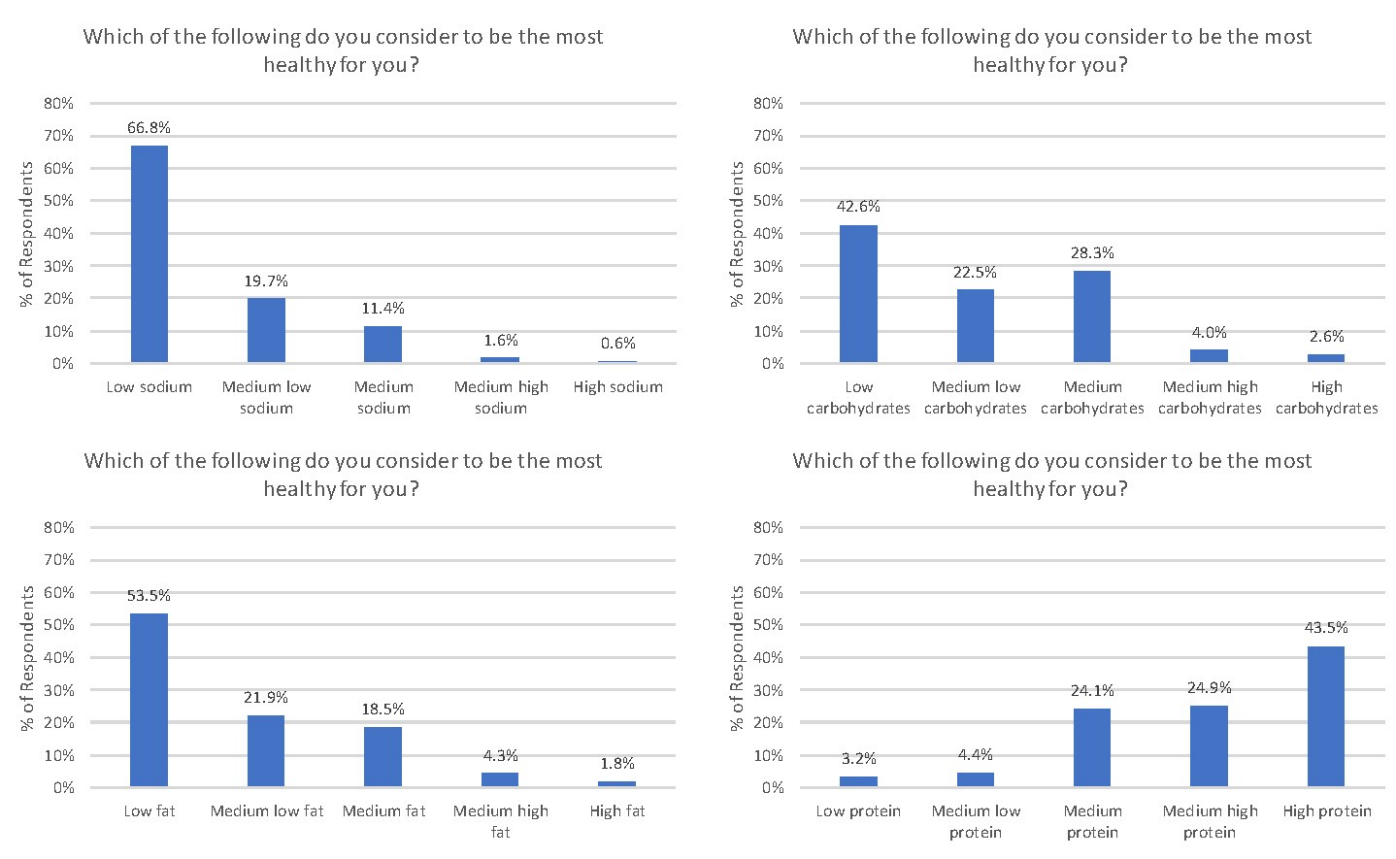
Perception of healthiness of sodium, carbohydrates, fat, and protein (*sampling error for each statistic is ±2*.*7%*.*)*.

A set of four questions was designed to evaluate how consumers perceived the healthiness of different nutrients and minerals. Consumers were asked, “Which of the following do you consumer to be most healthy for you”, and indicated low, medium low, medium, medium high, or high levels of sodium, carbohydrates, fat, and protein. Fig 4 shows that about two-thirds of respondents believed low sodium was most healthy for them. There were more disparate views about carbohydrates. A plurality of consumers thought a low amount of carbohydrates was most healthy, but 28.3% considered a medium amount of carbohydrates as most healthy. Low fat diet was considered healthiest by 53.5% of consumers, and another 21.9% thought medium low fat was healthiest. About 6% of respondents thought medium high or high fat diets were healthiest. In general, higher protein diets were considered healthier than lower protein diets.

**Fig 5:**
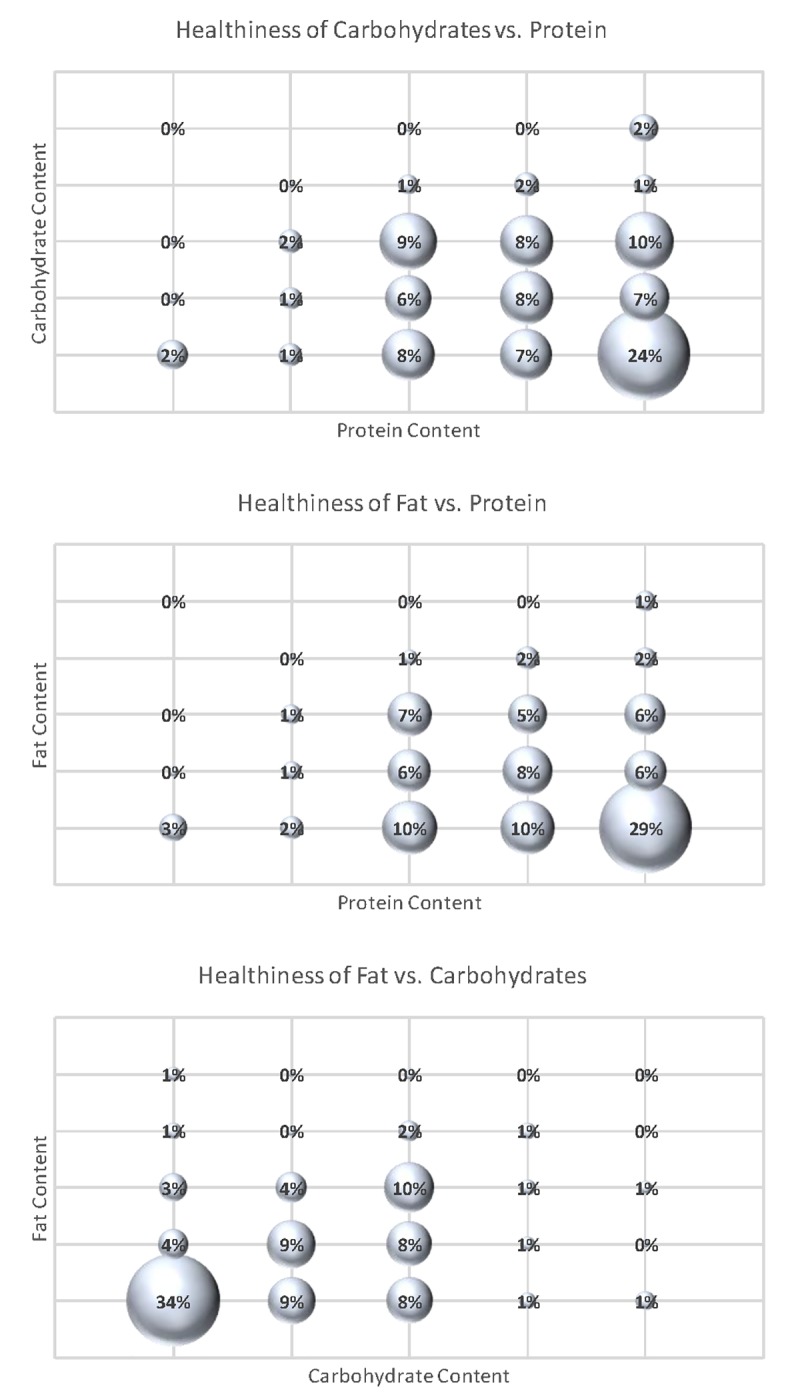
Joint perceptions of healthiness of carbohydrates, fat, and protein (*sampling error for each statistic is ±2*.*7%*.*)*.

Fig 4 indicates the most common category chosen by respondents in each category is at the extreme (lowest sodium, lowest fat, lowest carbohydrate, and highest protein). However, it is useful to consider how consumers evaluated the healthiness of combinations of these nutrients. Fig 4 illustrates the percent of respondents that indicated the healthiness of joint-combinations of carbohydrates, fat, and protein. As the top panel of Fig 5 reveals, 24% of respondents indicated the highest level of protein and lowest level of carbohydrates as healthiest. About 10% of respondents indicated high protein and medium carbohydrates as the next most healthy combination, followed by 9% who picked the medium level of both carbohydrates and protein as healthiest. The middle panel indicates 29% of respondents indicated highest protein and lowest fat levels as healthiest, followed by 20% who indicated low fat and medium or medium high protein as healthiest. The final panel in Fig 4 plots carbohydrates against fat. There were very few respondents (about 2%) who consider high fat, low carbohydrate diets as healthiest.

**Fig 6:**
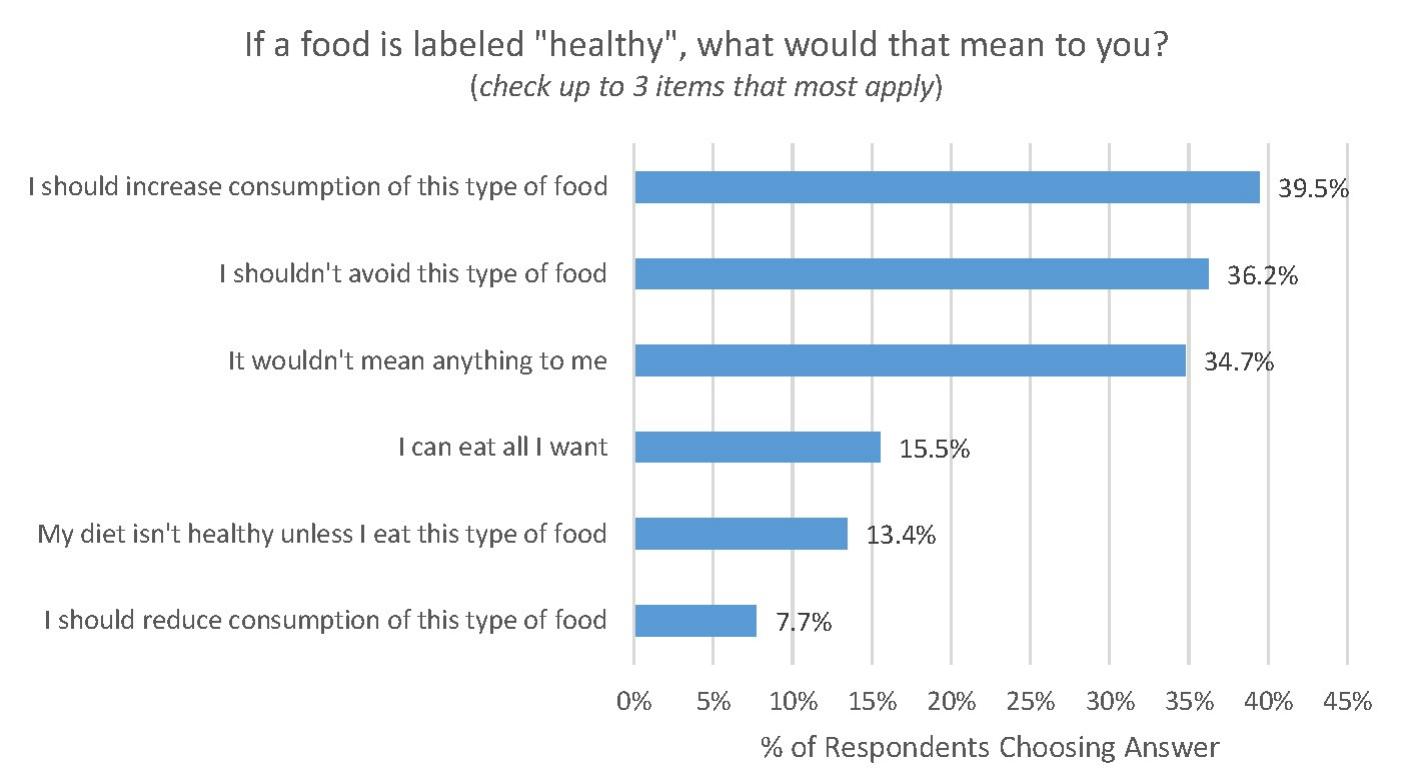
Behavioral implications of healthy food labels (*sampling error for each statistic is ±2*.*7%*.*)*.

Moving on from consumers’ definitions of foods and nutrients that are considered healthy or unhealthy, consumers were asked what they think “healthy” means in terms of behavior (Fig 6). About 40% of consumers thought a healthy label implied they should increase consumption of this type of food, and 15.5% thought the label meant they could eat all they wanted. A little over a third of respondents (34.7%) indicated that a healthy label would not mean anything to them.

Finally, consumers were asked how they thought healthy labels should be regulated. A majority of consumers (54.2%) felt the FDA should regulate the use of the term by requiring companies to follow a uniform, consistent definition. Thus, consumers want the FDA to define “healthy”; however, it is not clear that consumers agree on the definition. Indeed only 19.3% of respondents believed FDA should keep the current definition. About 17% indicated they believed the FDA should regulate to prevent the use of the term “healthy” on food packages, and 9/3% indicated that the FDA should not regulate the use of the term “healthy” on food packages.

## Conclusions

This report conveys the results of a nationwide survey of 1,290 U.S. food consumers, with primary focus on consumers’ perceptions of healthy food. The FDA has signaled efforts to re-define the terms for use on food labels, and as such, insights into how consumers define and interpret has been sought by the FDA [28].

Slightly more consumers than not thought a food could be deemed healthy based solely on the foods’ nutritional content. However, it was also the case that slightly more consumers than not thought healthiness is a characteristic of one’s overall diet and the combination of foods consumed rather than something that can be determined looking at individual foods. There were four broad types of consumers with about a quarter falling into each of four categories of views about healthy: food-nutrient focus, food-nonnutritive focus, diet-nutrient focus, and diet-nonnutritive focus. Such findings presenting something of a challenge because “healthy” labels appear on individual foods rather than on combinations of foods (i.e. an overall diet). Given that roughly half of consumers view “healthy” as a purview of one’s over-all diet rather than a specific food, it suggests food-specific healthy labels may be less impactful for a significant portion of the population. Moreover, current “healthy” labels are based exclusively on a food’s nutrient content, which according to half this survey sample, is insufficient to determine whether a food, for them, is considered healthy.

Ratings of individual food products according to healthiness reveals that “healthy” is not a single unifying construct, but rather consumers evaluate healthiness along a number of different dimensions or factors related to animal origin, preservation, and freshness/processing. Focusing on individual nutrients, perceived healthiness is generally decreasing in a food’s fat, sodium, and carbohydrate content and increasing in protein content. Only about 2% of consumers jointly rated high fat and low carbohydrates as the healthiest nutrient combination.

About 40% of consumers thought a healthy label implied they should increase consumption of the type of food bearing the label, and indeed about 15% thought the label meant they could eat all they wanted. A little over a third of respondents (34.7%) indicated that a healthy label would not mean anything to them.

While a slight majority of consumers (54%) felt the FDA should regulate the use of the term “healthy” by requiring companies to follow a uniform, consistent definition, only about 19% believed the FDA should keep the current definition, raising questions about how–exactly–consumers believe the term should be defined.

There are some limitations to the present study. The survey data originate from a non-probability based (or “opt-in”) panel maintained by a marketing research firm. Prior research suggests the accuracy of non-probability internet samples is lower than that of probability based phone or internet samples, although post-stratification weighting sometimes helps reduce inaccuracies [36]. Nonetheless, in recent years use of non-probability based internet samples has expanded rapidly in marketing and academic research as well as political polling [37, 38], and there is evidence that even highly non-representative samples are just as accurate as forecasting election outcomes as more traditional methods [39] and that differences between different probability based samples can be as large as the difference between probability-based and non-probability based samples [40]. The trend toward opt-in online panels may be explained in part by the significantly lower cost of non-probability based samples as well as emerging problems with conventional survey approaches. For example, [41] posits that the difference between opting out of a probability sample (which often have response rates less than 10%) and opting in to a non-probability sample is practically nill (see also [42]). Moreover, the emergence of mobile phones and unlisted numbers has made random-digit dialing techniques less reliable [37] and there has been a significant decline in response rates and data quality even in “high quality” government surveys [43]. The use of post-stratification weights in this study forces the sample to match the population in terms of selected demographic variables, and any degree of inaccuracy arising from the use of a non-probability based sample is unknown.

There are a number of avenues for future research. This survey included questions asking about health perceptions of macro nutrients (i.e., fat, protein, carbohydrates). Future research on perceptions of micronutrients such as different types of fats (e.g., saturated vs. unsaturated) or carbohydrates (e.g., whole vs. refined grains; added vs. natural sugars) would also provide useful information relative to health perceptions. It could also be of interest to inquire about other policy preferences. In this study, respondents were asked a broad question about consumer’s perception of the FDA’s role in regulating and defining a claim like “healthy.” It might also be useful, however, to inquire as to whether and how consumers believe, conditional on the fact the FDA already defines healthy, the label definition should be updated over time.

Overall, results of this study suggest consumer’s perceptions of “healthy,” which is primarily based on fat content, partially aligns with the FDA definition, but findings also suggest consumers perceive the word as a broader and more nuanced concept that defies easy, uniform definition. Given heterogeneity in beliefs about the definition of healthy, findings from this study suggest such labels may need to be accompanied with nutrition education or that on-package disclosures may need to accompany health claims so that consumers know what, precisely, is being communicated. Nationwide nutrition education around the label claim “healthy” is likely to be costly and results of such a campaign are uncertain. Such considerations point to on-package definitions/disclosures as a more effective means of communicating the federal definition of “healthy.”

## Supporting information

S1 File CRA Survey topline results. (PDF)

S1 Appendix (Table A) Demographic Characteristics of Respondents. (Table B) Means and Correlations of Health Perceptions of 15 Foods. (Table C) Results of Factor Analysis Applied to Health Perceptions of 15 Foods: Rotated Factor Pattern (Standardized Regression Coefficients).
(DOCX)
