# Supplementary material for: Consumer beliefs about healthy foods and diets
Source: PLoS One. 2019 Oct 15;14(10):e0223098. doi: 10.1371/journal.pone.0223098 (PMC6793866; doi:10.1371/journal.pone.0223098)
Supplement: S1 File — (PDF) [file pone.0223098.s001.pdf]

Corn Refiners Association Survey

Topline Results

Author: Jayson Lusk

Date: 1/2/19

This document reports the topline results of the responses to a nationwide U.S. of 1,290 respondents who met exclusionary criteria. This sample size provides a margin of error of plus or minus 2.7%. Responses are weighted to match the U.S. population in terms of gender, age, education, and region of residence. The survey was fielded in mid-December 2018.

Q2 How much of the grocery shopping do you do for your household?
I buy almost all the groceries for my household (68.1%)
I buy most of the groceries for my household (15.8%)
I buy about half of the groceries for my household (16.2%)
I buy less than half the groceries for my household (excluded)
I rarely buy the groceries for my household (excluded)

Q3 Is there additional information you would like to see on food packages that is not typically there?
Yes (29.6%)
No (70.4%)

Display This Question:
If Q3 = Yes

Q4 What additional information would you like to see on food packages? *(please type your answer in the blank below)*
<<see report for full analysis of this open ended question>>

Q5 Which of the following labels or information on food packages do you find to be most informative? (*check up to 3 items that most apply*)

health claim	8.6%
natural label	9.0%
organic label	10.8%
origin label	11.6%
healthy label	14.3%
non-GMO label	15.8%
brand name	29.5%
package weight or volume	33.3%
nutrition facts panel	61.1%
ingredient list	69.0%

Q6 How important are the following items to you when deciding which foods to buy? *(please drag 3 items in to the most important box and 3 items into the least important box)*

Most Important (Place 3 items here)	Least Important (Place 3 items here)
Taste (72.7%)	Convenience (57.2%)
Price (69.9%)	Familiarity (56.6%)
Healthiness (54%)	Appearance (50.2%)
Safety (32.3%)	Environmental Impact (49.7%)
Naturalness (22.5%)	Naturalness (37%)
Familiarity (15.5%)	Safety (15.5%)
Convenience (11.9%)	Price (14.5%)
Appearance (11.8%)	Healthiness (13.9%)
Environmental Impact (9.4%)	Taste (5.4%)

Food Value	% Most - % Least
Taste	67.3%
Price	55.4%
Healthiness	40.2%
Safety	16.8%
Naturalness	-14.6%
Appearance	-38.5%
Environmental Impact	-40.3%
Familiarity	-41.1%
Convenience	-45.3%

Q7 What does it mean to you for a food to be called "natural"? *(please type one or two words in the blank below)*

<<see report for full analysis of this open ended question>>

Q8 What does it mean to you for a food to be called "healthy"? *(please type one or two words in the blank below)*

<<see report for full analysis of this open ended question>>

Start of Block: Natural

Q9 If a food was produced with the following processes or practices, would you consider the food to be natural or not natural?

	Natural	Neither Natural or Not	Not Natural
Chopped (e.g., baby carrots, sliced apples, cut watermelon)	76.9%	17.8%	5.3%
Vegetable oil from pressed grains like soybeans, corn, or canola	51.3%	32.6%	16.2%
Fermentation (e.g., sauerkraut, yogurt, bread, beer)	50.4%	31.4%	18.1%
Preserved by canning, freezing, pasteurization, or smoking	41.0%	35.1%	23.9%
Preserved with sugar, salt, or vinegar	36.6%	31.7%	31.7%
Washing (e.g., cleaning grains or fruits and vegetables using organic acids or cholrine)	36.2%	32.9%	31.0%
Extracted starch from corn, potatoes, or wheat	32.9%	38.9%	28.2%
Flavor enhanced with sugar, salt, or smoking	24.4%	31.6%	44.1%
Added enzymes to improve texture (e.g., in cheese or bread)	11.9%	31.3%	56.8%
Appearance altered through decoloring or bleaching (e.g., flour, sugar, rice)	6.9%	21.2%	71.9%
Preserved with benzoates, nitrites, sulphites, or sorbates	6.6%	19.6%	73.8%
Flavor enhanced with MSG, guanylic acid, or Monopotassium glutamate	6.1%	15.1%	78.9%

Q10 What does it mean to you for a food to be called "natural"? (*check up to 3 items that most apply*)

no preservatives	58.8%
no hormones or antibiotics	53.5%
no pesticide residues	37.7%
fresh	29.4%
organic	26.0%
close to nature	22.8%
few added ingredients	22.3%
foods my grandmother would recognize	7.1%
anything I could make in my kitchen	4.7%
local	4.1%
uncooked	3.0%

Q11 Which of the following foods or processes do you consider to be natural? (*click up to 5 items on the image that you believe are natural*)

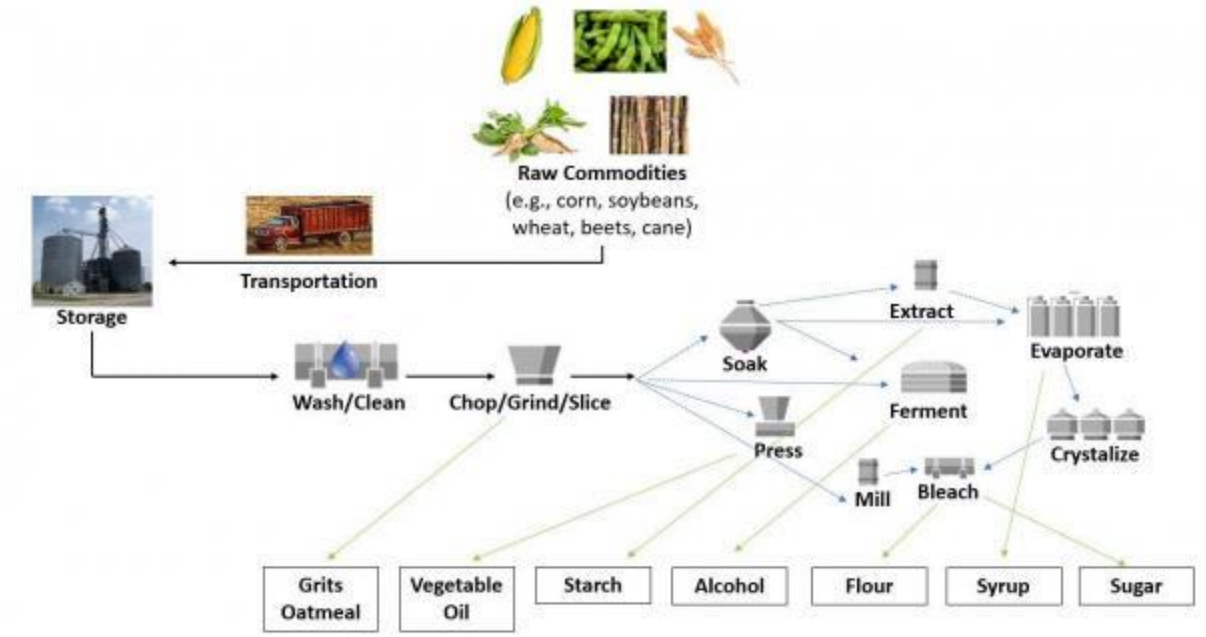

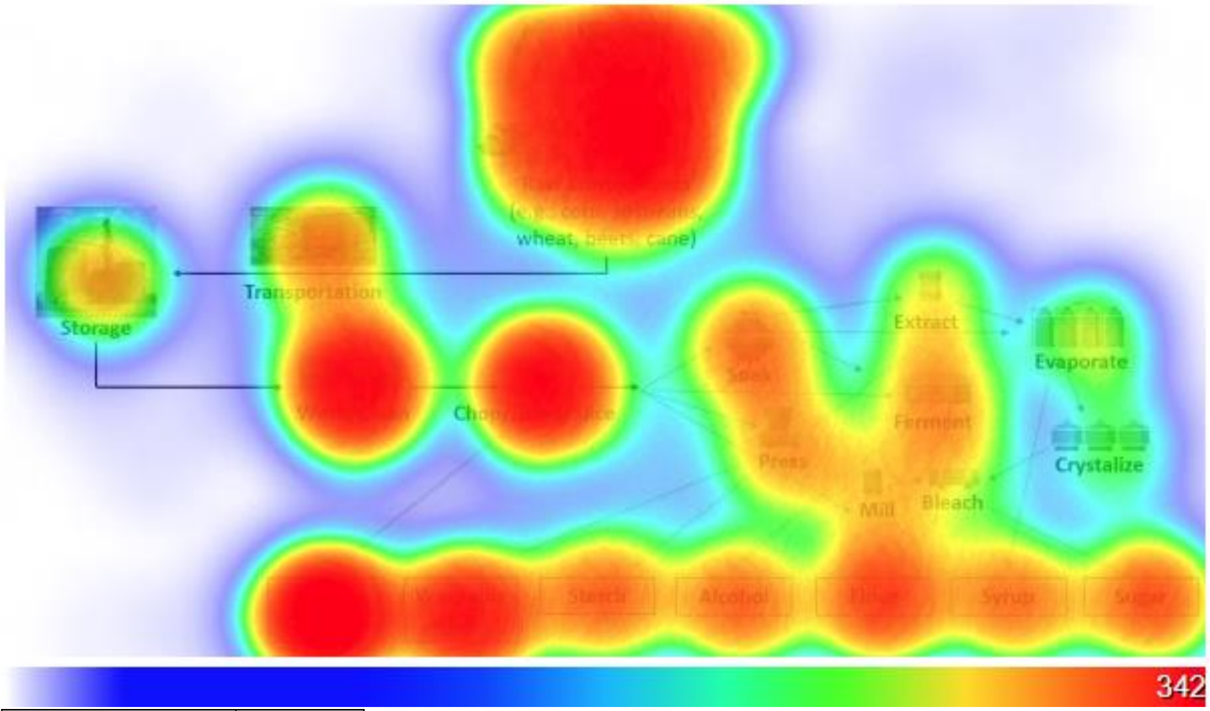

Raw Commodity	47.1%
Grits/Oatmeal	28.9%
Wash/Clean	26.3%
Chop/Grind/Slice	23.8%
Vegetable Oil	19.8%
Flour	13.3%

Transportation	12.9%
Starch	10.5%
Alcohol	10.2%
Soak	9.7%
Sugar	8.6%
Syrup	7.9%
Storage	7.7%
Ferment	7.1%
Press	6.3%
Extract	4.7%
Mill	4.0%
Evaporate	3.6%
Crystalize	2.2%
Bleach	1.2%

Q12 Which of the following foods or processes do you consider to be NOT natural? (*click up to 5 items on the image that you believe are NOT natural*)

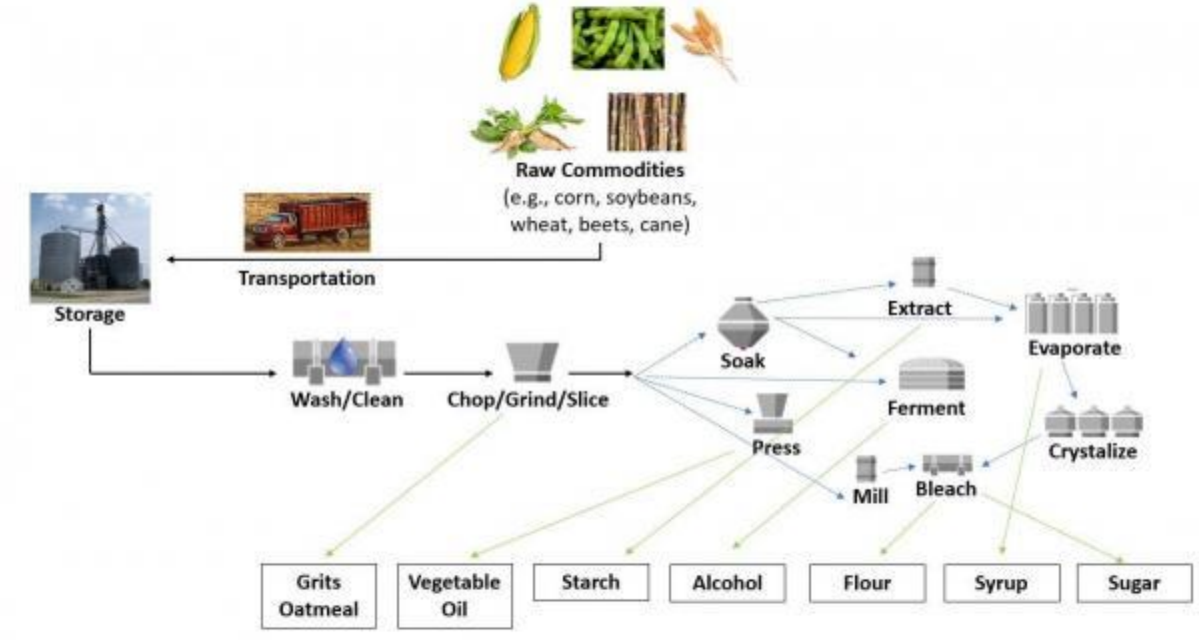

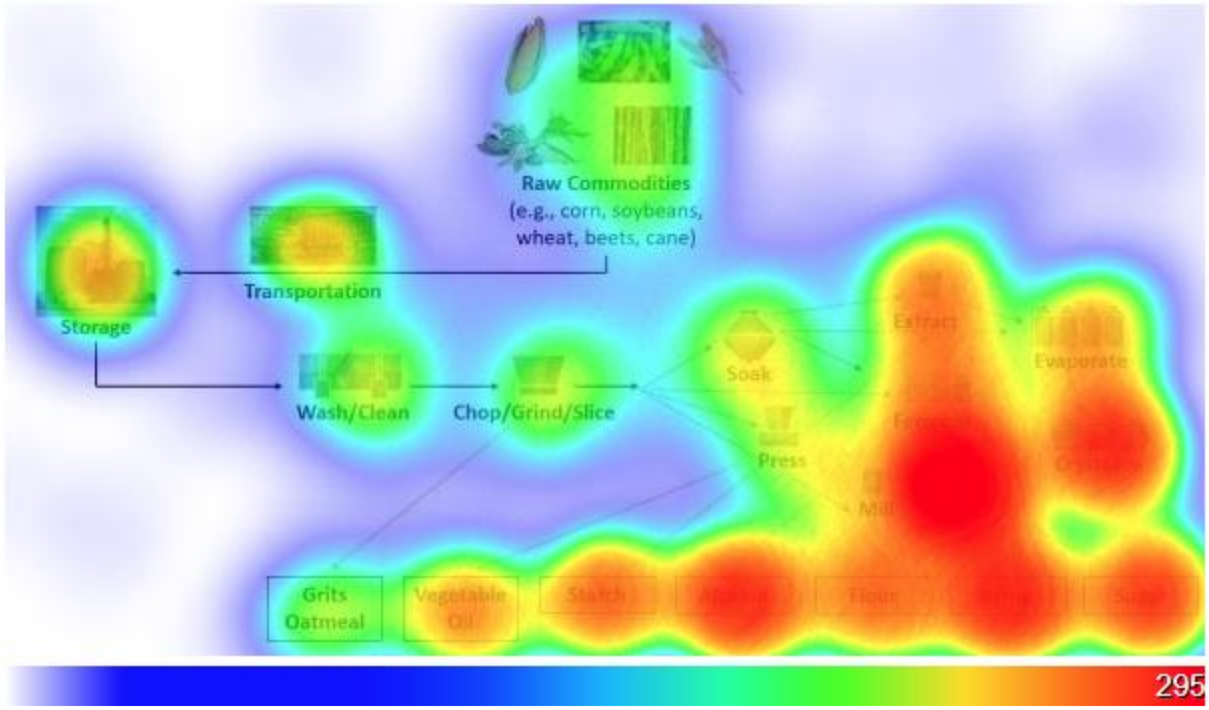

Bleach	33.8%
Crystalize	22.2%
Alcohol	21.7%
Syrup	20.1%
Sugar	15.3%
Starch	13.4%
Flour	13.2%
Extract	12.0%
Ferment	11.7%
Vegetable Oil	10.7%
Storage	9.7%
Evaporate	9.4%
Transportation	9.2%
Raw Commodity	5.4%
Wash/Clean	5.1%
Press	4.3%
Chop/Grind/Slice	4.0%
Soak	3.9%
Mill	3.7%
Grits/Oatmeal	2.7%

Q13 If a food was produced from a grain, oilseed, fruit, or vegetable that was grown or farmed using the following processes or practices, would you consider the food to be natural or not natural?

	Natural	Neither	Not Natural
organically grown crops	83.3%	13.4%	3.4%
crops grown indoors in greenhouses	70.4%	23.9%	5.7%
hydroponically grown crops	44.3%	34.6%	21.1%
hybrid seeds	29.1%	37.7%	33.2%
crops sprayed with natural pesticides like copper sulfate or bacillus thuringiensis	23.0%	32.6%	44.4%
seeds created with genetic engineering or GMOs	11.6%	22.9%	65.5%
seeds created using gene editing	11.1%	27.5%	61.5%
seeds created using mutagenesis	9.5%	37.8%	52.7%
crops sprayed with synthetic pesticides like glyphosate or chlorpyrifos	7.1%	17.7%	75.2%

Q14 To what extent do you agree or disagree with each of the following statements?

	Strongly Disagree	Somewhat Disagree	Neither Agree nor Disagree	Somewhat Agree	Strongly Agree
Natural food is healthier	2.7%	3.1%	17.4%	39.9%	36.9%
Natural food is better for the environment	2.3%	4.5%	24.4%	37.9%	31.0%
Natural food is tastier	4.0%	11.9%	40.2%	25.7%	18.3%
Natural food is more affordable	23.6%	36.9%	22.0%	11.0%	6.5%
Natural food is safer to eat	2.7%	4.8%	20.9%	38.0%	33.5%
Natural food is more convenient to eat	6.8%	21.1%	42.7%	18.0%	11.4%
Natural food is more sustainable	3.2%	9.7%	35.7%	28.9%	22.4%

Q15 Currently, the Food and Drug Administration (FDA) does not define the term "natural" on food packages but food manufacturers are allowed to use the label. How do you believe natural labels should be regulated?

The FDA should regulate to use of the term "natural" by requiring companies to follow a uniform, consistent definition (65.7%)

The FDA should regulate to prevent the use of the term "natural" on food packages (21.5%)

The FDA should not regulate the use of the term "natural" on food packages (12.8%)

Q16 To what extent do you trust or distrust the FDA to define the term "natural" in a way that you would find useful in making food choices?

Highly trust	10.0%
Somewhat trust	34.4%
Neither trust nor distrust	33.0%
Somewhat distrust	14.9%
Highly distrust	7.7%

Q17 The U.S. Department of Agriculture (USDA) regulates the use of the label "natural" for meat. Which of the following best matches the current USDA definition for "natural" meat?

no hormones	31.3%
minimally processed	26.6%
no antibiotics	23.8%
grass fed	14.5%
cage free	3.8%

Start of Block: Healthy

Q18 Do you consider each of the following foods to be healthy or unhealthy for you?

	Healthy	Neither Healthy nor Unhealthy	Unhealthy
Fresh vegetables	96.2%	3.0%	0.9%
Fresh fruit	95.6%	3.9%	0.5%
Fish	87.8%	10.2%	2.1%
Chicken	79.5%	16.7%	3.8%
Eggs	79.2%	18.2%	2.6%
Milk	70.4%	22.6%	7.0%
Frozen vegetables	69.7%	26.6%	3.7%
Frozen fruit	63.8%	31.1%	5.2%
Cheese	57.3%	33.3%	9.5%
Beef	52.4%	35.1%	12.6%
Canned vegetables	43.2%	41.4%	15.4%
Canned fruit	37.3%	41.2%	21.6%
Vegetable oil	25.0%	49.2%	25.8%
Bakery and cereal items	23.3%	43.7%	33.0%
Candy	4.1%	16.1%	79.8%

Q19 Which of the following affects whether or not you would consider a food healthy for you? (*check up to 3 items that most apply*)

sugar content	23.7%
use of hormones or antibiotics	23.5%
fat content	19.2%
pesticide residues	18.4%
use of preservatives	15.9%
use of GMOs	12.9%
number of calories	12.6%
organic	10.9%
protein content	10.3%
vitamin content	9.0%
use of processing	8.6%
fiber content	5.1%
local	2.7%
none of the above	0.0%

Q20 Which of the following diseases or health conditions do you think would affect which foods you consider to be healthy? (*check up to 3 items that most apply*)

diabetes	63.3%
heart disease	54.8%
obesity	50.1%
food allergy	30.5%
cancer	29.5%
none of the above	0.0%

Q21 Which of the following do you consider to be the most healthy for you?

Low sodium	66.8%
Medium low sodium	19.7%
Medium sodium	11.4%
Medium high sodium	1.6%
High sodium	0.6%

Q22 Which of the following do you consider to be the most healthy for you?

Low carbohydrates	42.6%
Medium low carbohydrates	22.5%
Medium carbohydrates	28.3%
Medium high carbohydrates	4.0%
High carbohydrates	2.6%

Q23 Which of the following do you consider to be the most healthy for you?

Low fat	53.5%
Medium low fat	21.9%
Medium fat	18.5%
Medium high fat	4.3%
High fat	1.8%

Q24 Which of the following do you consider to be the most healthy for you?

Low protein	3.2%
Medium low protein	4.4%
Medium protein	24.1%
Medium high protein	24.9%
High protein	43.5%

Q25 Which of the following statements best matches your view?

If I know the nutrient content of the food (the amount of fat, protein, carbs, vitamins, etc.), I know enough to decide whether a food is healthy (52.1%)

I need to know more than just the nutrient content (the amount of fat, protein, carbs, vitamins, etc.) of a food to decide whether it is healthy (47.9%)

Q26 Which of the following statements best matches your view?

It is better to think about "healthy" on a food-by-food basis (some foods are healthy and some aren't) (47.9%)

It is better to think about "healthy" by looking at a whole dietary pattern (healthiness is defined by combinations of foods in a diet) (52.1%)

Q27 If a food is labeled "healthy", what would that mean to you? (*check up to 3 items that most apply*)

I should increase consumption of this type of food	39.5%

I shouldn't avoid this type of food	36.2%
It wouldn't mean anything to me	34.7%
I can eat all I want	15.5%
My diet isn't healthy unless I eat this type of food	13.4%
I should reduce consumption of this type of food	7.7%

Q28 To what extent do you agree or disagree with each of the following statements?

	Strongly Disagree	Somewhat Disagree	Neither Agree nor Disagree	Somewhat Agree	Strongly Agree
Healthy food is safer to eat	2.3%	4.8%	26.0%	39.5%	27.5%
Individual needs determine whether various foods are healthy for an individual	1.4%	6.4%	21.2%	43.8%	27.2%
Healthy food is more natural	2.9%	5.5%	24.4%	41.5%	25.7%
Healthy food is better for the environment	3.5%	5.5%	38.3%	30.6%	22.1%
Healthy food is more sustainable	2.7%	9.2%	40.2%	29.8%	18.1%
Healthy food is tastier	5.7%	16.4%	43.9%	20.7%	13.4%
Healthy food is more convenient to eat	9.2%	24.1%	42.2%	14.4%	10.1%
Healthy food is more affordable	22.3%	35.5%	27.8%	7.6%	6.8%

Q29 Currently, the Food and Drug Administration (FDA) allows the label "healthy" to be used on foods low in fat and saturated fat and that provide at least 10% of recommended amounts of vitamin A, vitamin C, calcium, iron, protein, or fiber.

How do you believe healthy labels should be regulated in the future?

The FDA should regulate the use of the term "healthy" by requiring companies follow a uniform, consistent definition	54.2%
The FDA should keep the current definition of "healthy"	19.3%
The FDA should regulate to prevent the use of the term "healthy" on food packages	17.2%
The FDA should not regulate the use of the term "healthy" on food packages	9.3%

Q30 To what extent do you trust or distrust the FDA to define the term "healthy" in a way that you would find useful in making food choices?

Highly trust	8.9%
Somewhat trust	37.3%
Neither trust nor distrust	31.1%
Somewhat distrust	15.8%
Highly distrust	7.0%

Start of Block: Ingredients

Q31 Which of the two words below would you find more informative if you saw it on an ingredient list of a food package? *(Both words mean the same thing but one is the precise scientific description the other is the common usage)*

Maltodextrin (10.9%)
Corn starch (89.1%)

Q32 Which of the two words below would you find more informative if you saw it on an ingredient list of a food package? *(Both words mean the same thing but one is the precise scientific description the other is the common usage*)

Pyridoxine (9.7%)
Vitamin B-6 (90.3%)

Q33 Which of the two words below would you find more informative if you saw it on an ingredient list of a food package? (*Both words mean the same thing but one is the precise scientific description the other is the common usage*)

Resistant maltodextrin (13.4%)
Soluble corn fiber (86.6%)

<<There are 10 sets of questions, asking respondents in indicate the most and least attractive areas of a cereal box in addition to their purchase intention. Respondents were randomly assigned to answer 2 of the 10 sets of questions.>>

Which Area of the Box is Most Attractive?

				% of Respondents Clicking on Area of the Box		
Question Set	Price	Label(s)		Label	Price	Other Area
1	$2.98	none		1.3%	27.6%	71.1%
2	$4.98	none		0.8%	57.9%	41.3%
3	$2.98	natural		17.7%	29.0%	53.4%
4	$4.98	natural		14.6%	49.4%	36.0%
5	$2.98	healthy		22.6%	21.7%	55.7%
6	$4.98	healthy		19.3%	44.2%	36.4%
7	$2.98	organic		19.3%	27.0%	53.8%
8	$4.98	organic		11.7%	39.8%	48.5%
9	$2.98	natural, healthy, and organic		33.4%	23.2%	43.5%
10	$4.98	natural, healthy, and organic		27.1%	39.7%	33.2%

Which Area of the Box is Least Attractive?

				% of Respondents Clicking on Area of the Box		
Question Set	Price	Label(s)		Label	Price	Other Area
1	$2.98	none		87.7%	1.5%	10.9%
2	$4.98	none		98.5%	0.0%	1.6%
3	$2.98	natural		66.0%	24.8%	9.2%
4	$4.98	natural		71.2%	26.5%	2.3%
5	$2.98	healthy		68.4%	21.5%	10.2%
6	$4.98	healthy		76.4%	21.7%	1.9%
7	$2.98	organic		64.8%	28.5%	6.7%
8	$4.98	organic		65.3%	33.0%	1.7%
9	$2.98	natural, healthy, and organic		53.2%	43.4%	3.5%
10	$4.98	natural, healthy, and organic		55.7%	41.9%	2.4%

Purchase Intention

				Likelihood of purchase (0 = no chance of buying; 100 = 100% chance of buying)		
Question Set	Price	Label(s)		Mean	Median	Stdev
1	$2.98	none		37.8	35	29.1
2	$4.98	none		34.9	30	27.2
3	$2.98	natural		45.3	50	28.6
4	$4.98	natural		36.7	39	25.6
5	$2.98	healthy		43.1	50	28.0
6	$4.98	healthy		38.2	37	28.3
7	$2.98	organic		44.1	49	26.9
8	$4.98	organic		42.2	49	27.7
9	$2.98	natural, healthy, and organic		44.0	49	30.3
10	$4.98	natural, healthy, and organic		41.0	40	27.9

Start of Block: Cereal1 Control Low Price

Q34 Which part of the cereal box below is <u>most</u> attractive to you? *(please click or touch the image in the most attractive location)*

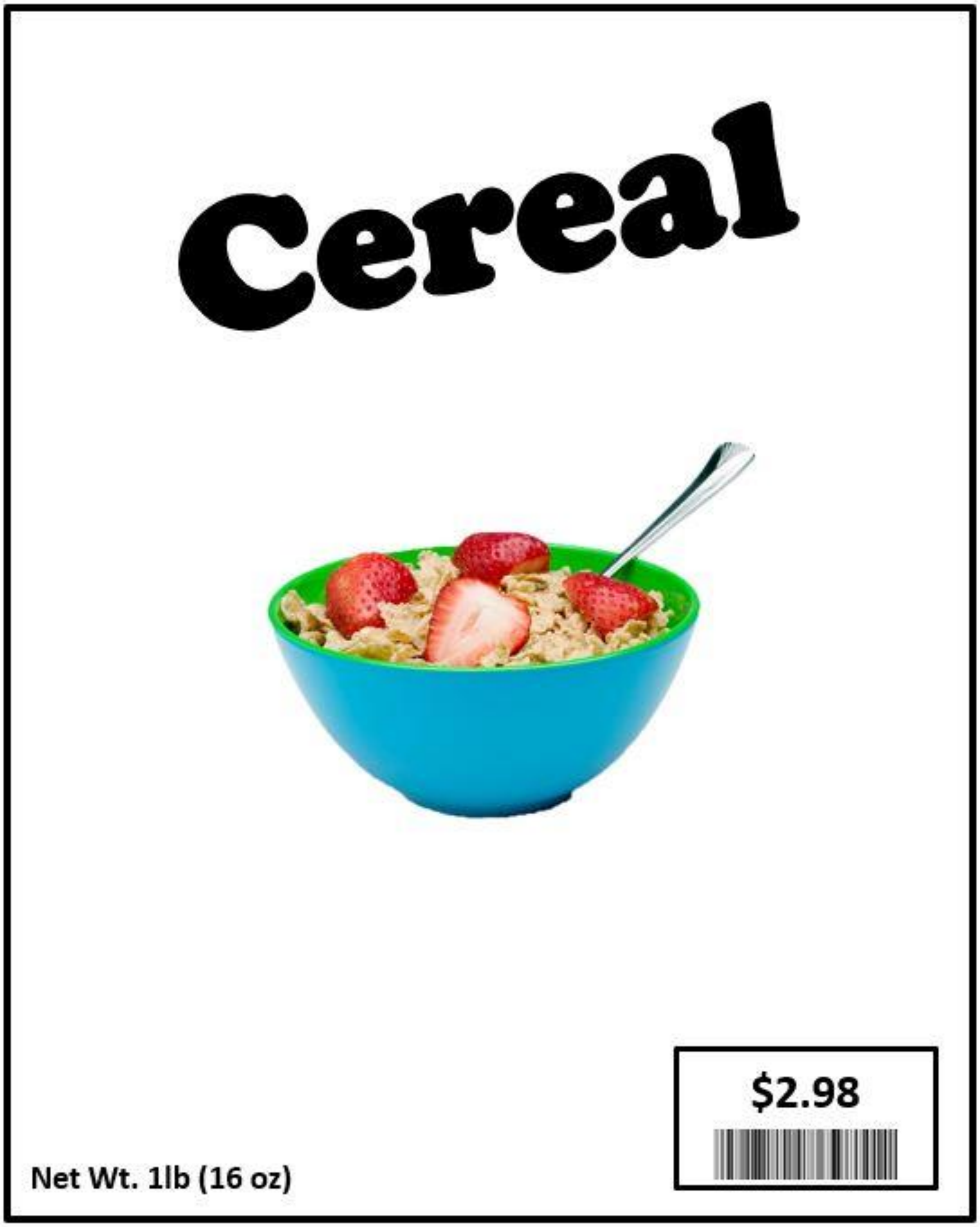

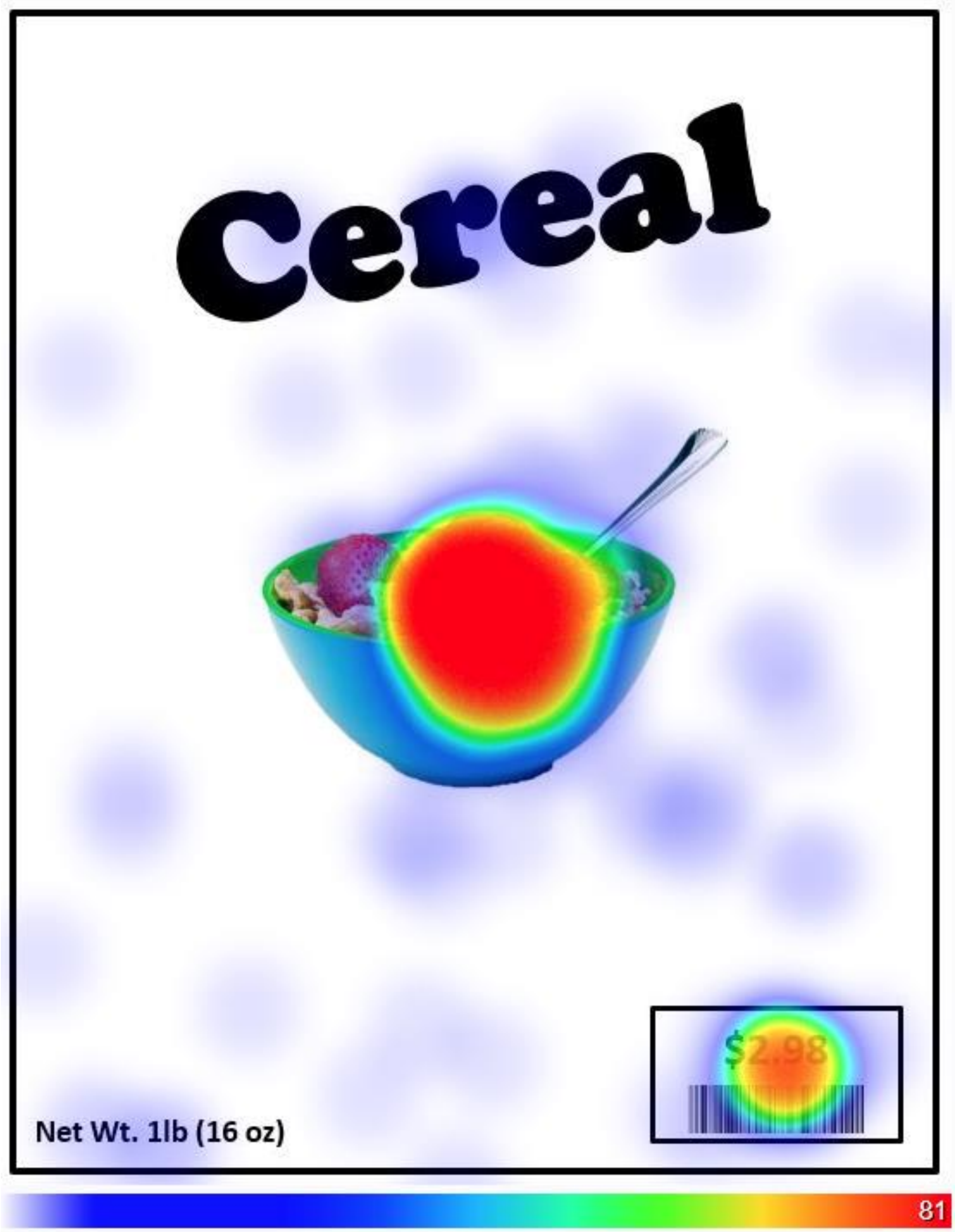
Cereal
$2.98
Net Wt. 1lb (16 oz)
81

Q35 Which part of the cereal box below is <u>least</u> attractive to you? *(please click or touch the image in the most attractive location)*

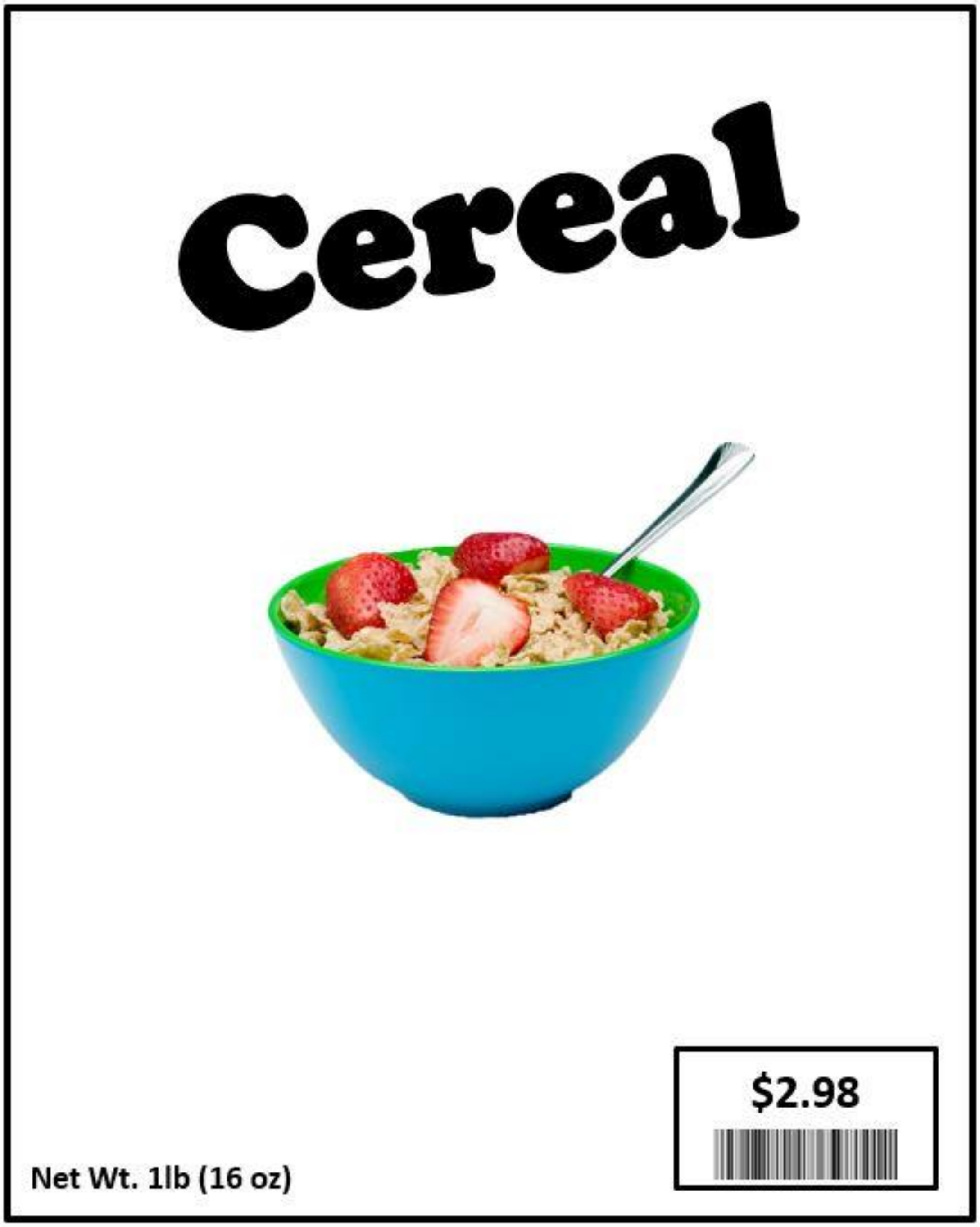

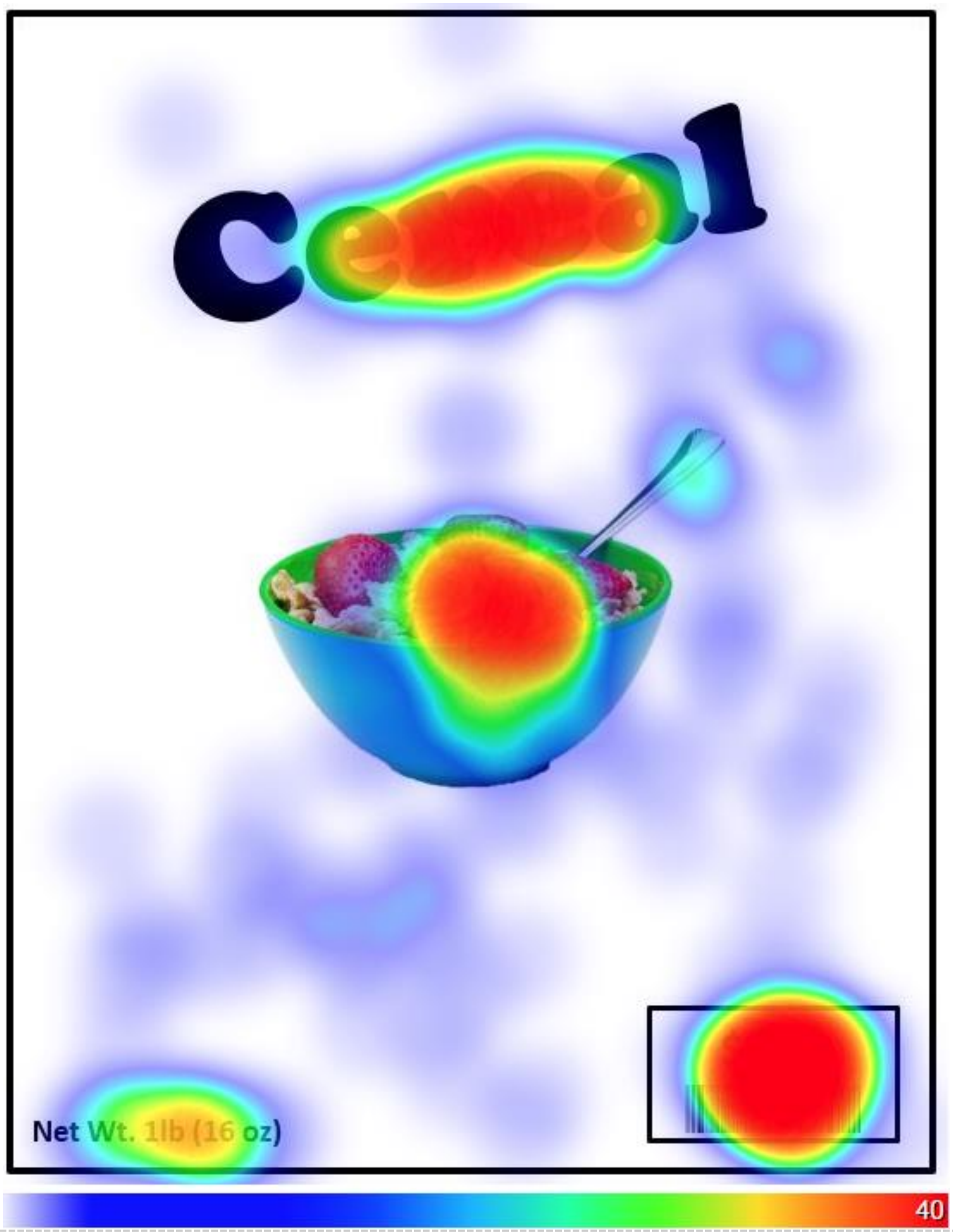

Q36 If you encountered this box of cereal in the grocery store, how likely would you be to purchase it? *(0 = no chance of buying; 100 = 100% chance of buying)*

0%=Very unlikely Very likely=100%

0 10 20 30 40 50 60 70 80 90 100

. ()	

Start of Block: cereal2_control_high

Q37 Which part of the cereal box below is <u>most</u> attractive to you? ***(please click or touch the image in the most attractive location)***

Cereal

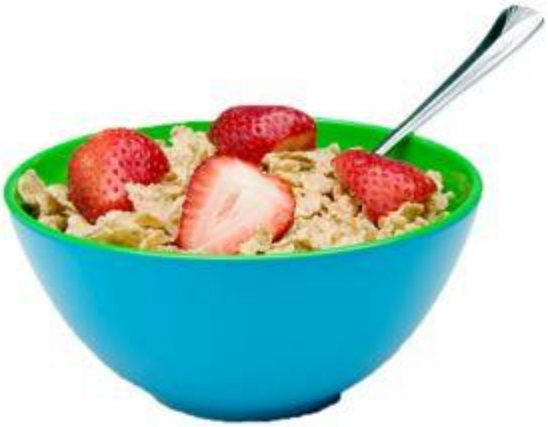

$4.98

Net Wt. 1lb (16 oz)

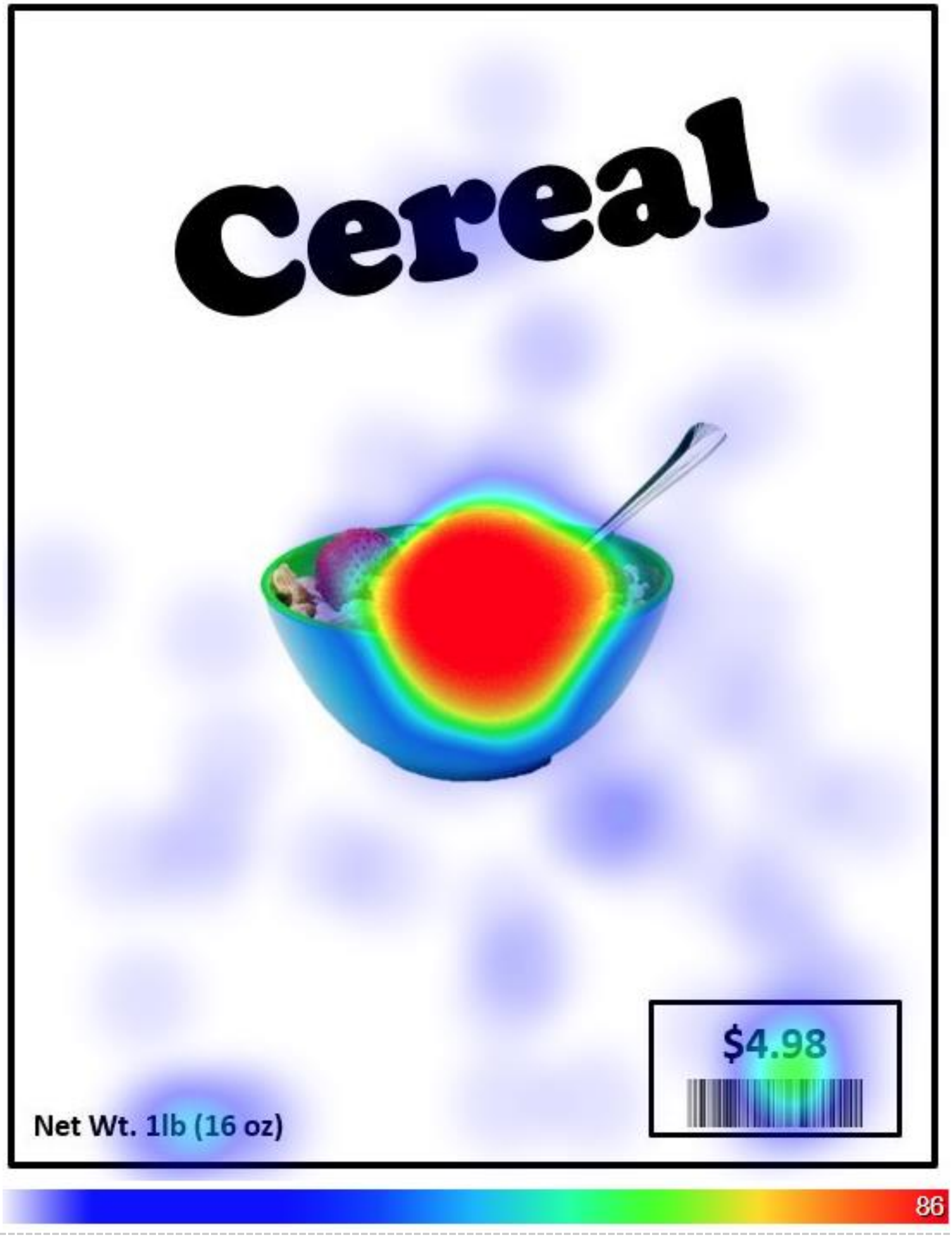
Cereal
$4.98
Net Wt. 1lb (16 oz)
86

Q38 Which part of the cereal box below is <u>least</u> attractive to you? *(please click or touch the image in the most attractive location)*

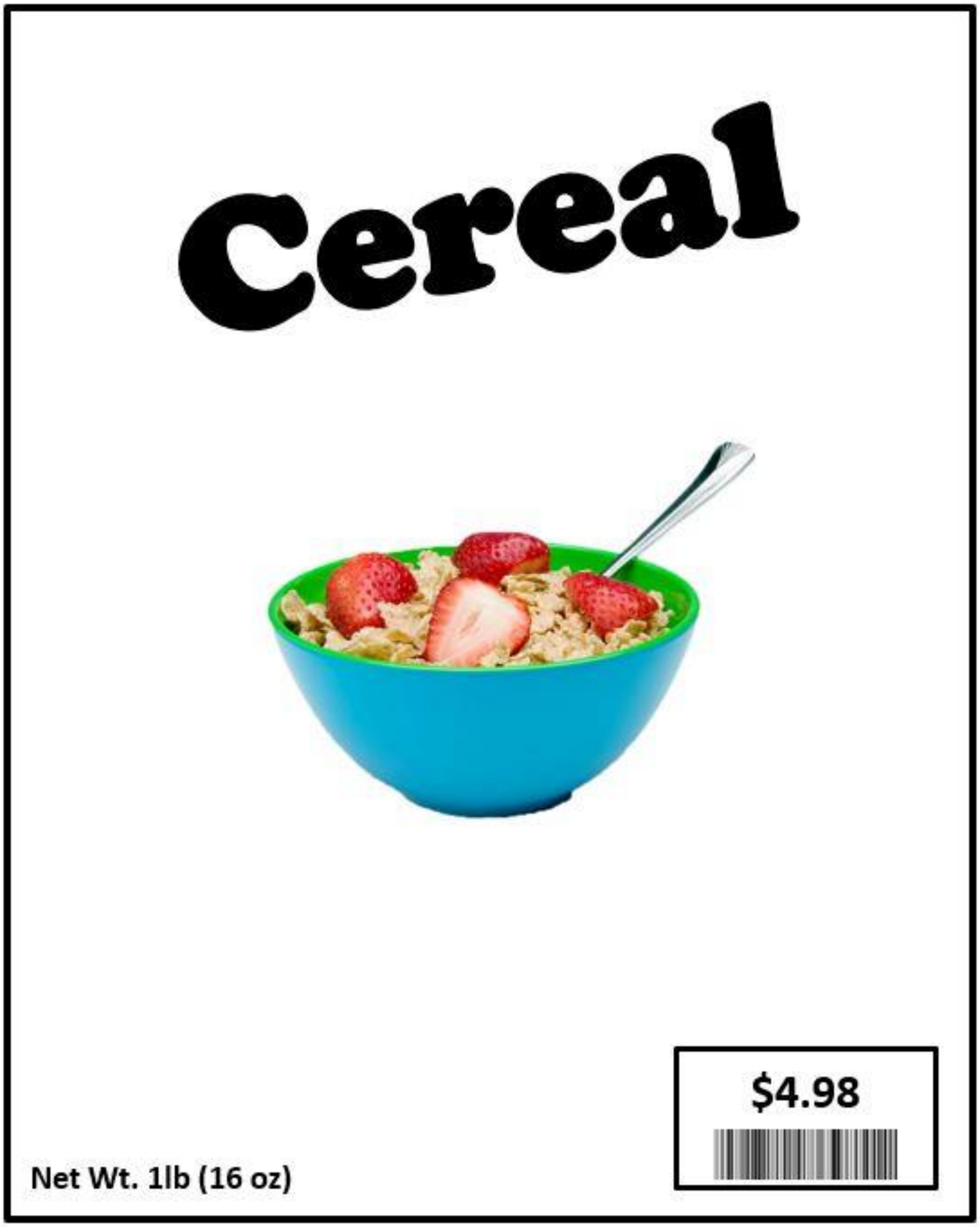

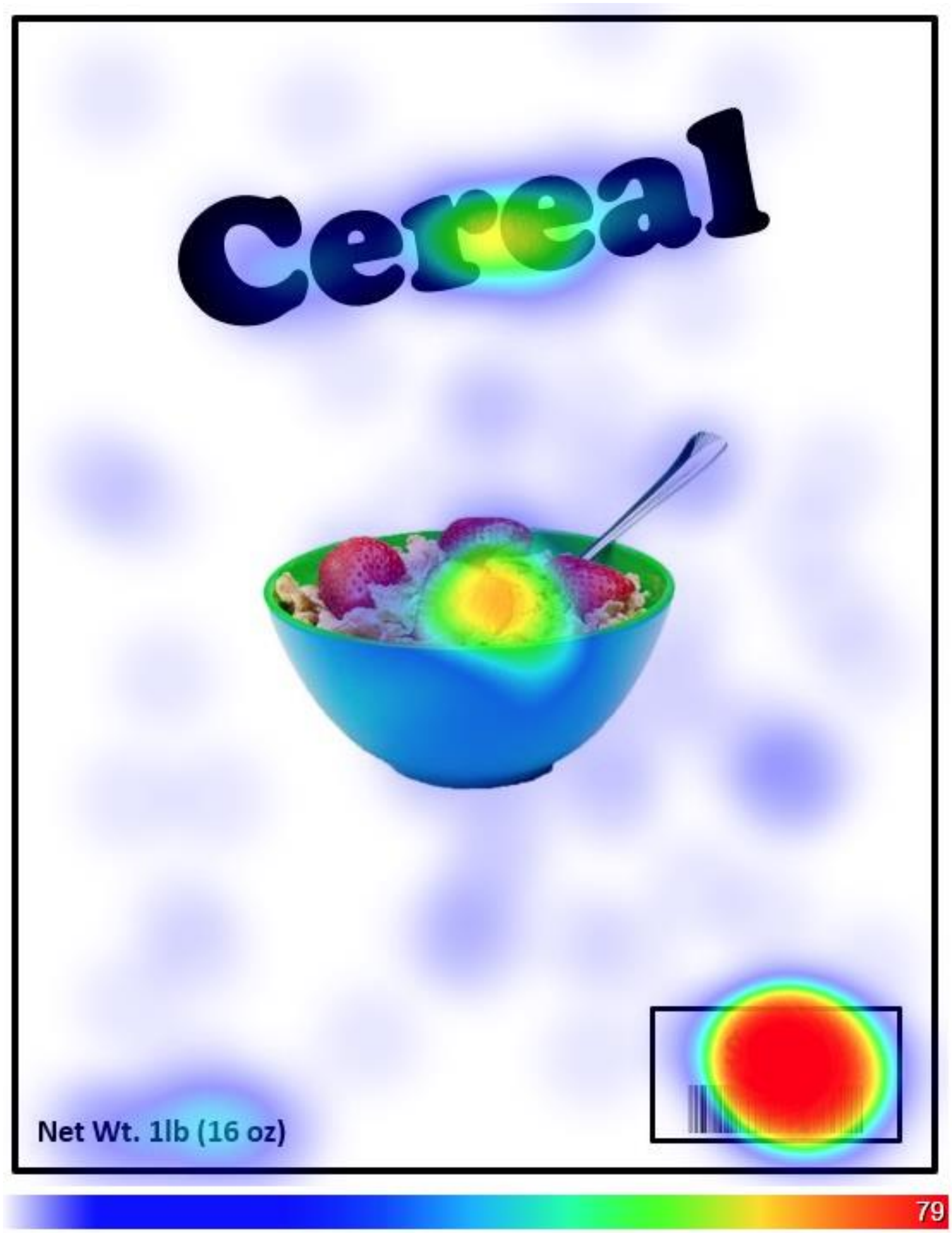

Q39 If you encountered this box of cereal in the grocery store, how likely would you be to purchase it? (*0 = no chance of buying; 100 = 100% chance of buying*)

End of Block: cereal2_control_high

Start of Block: cherrio3_natural_low

Q40 Which part of the cereal box below is <u>most</u> attractive to you? *(please click or touch the image in the most attractive location)*

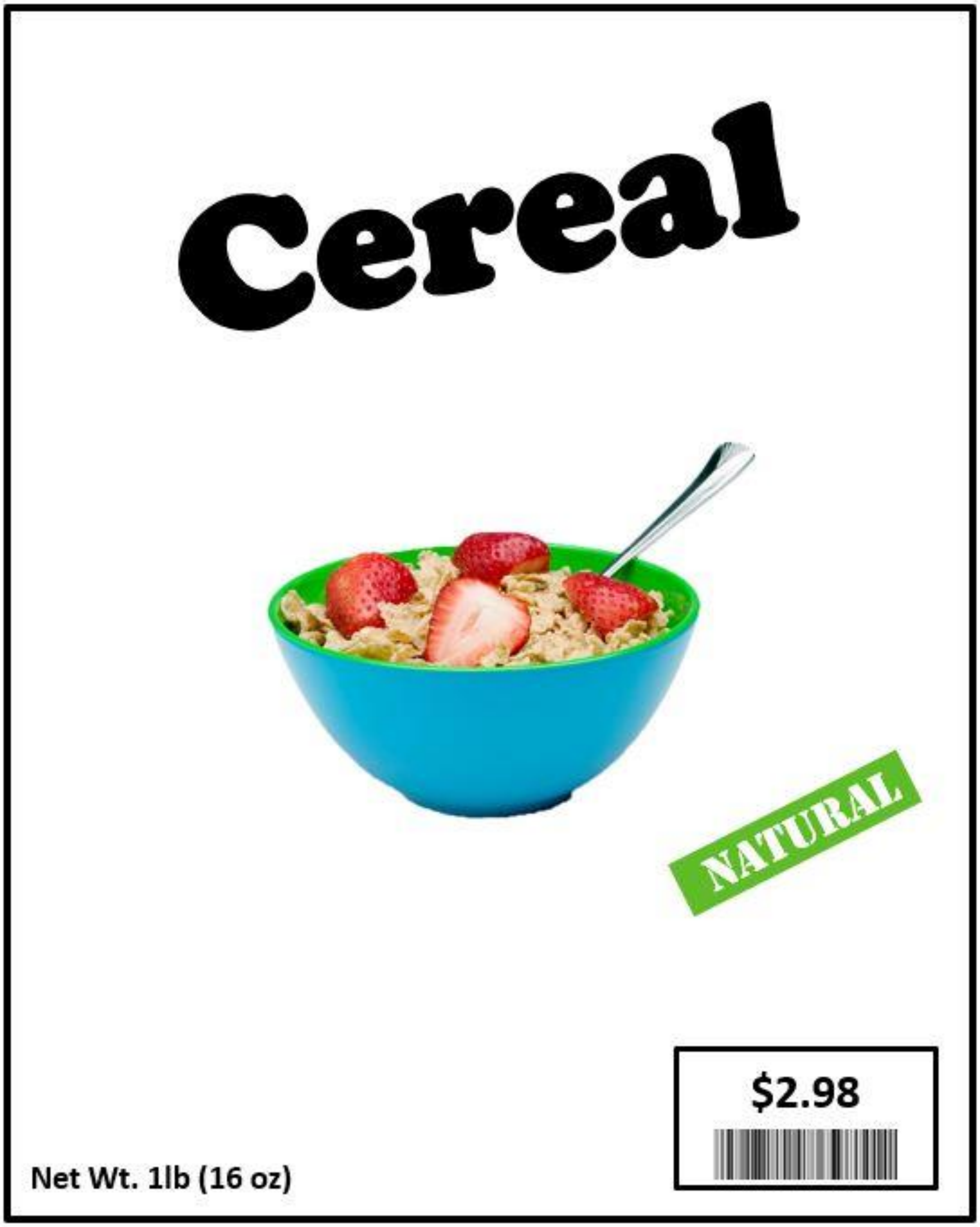

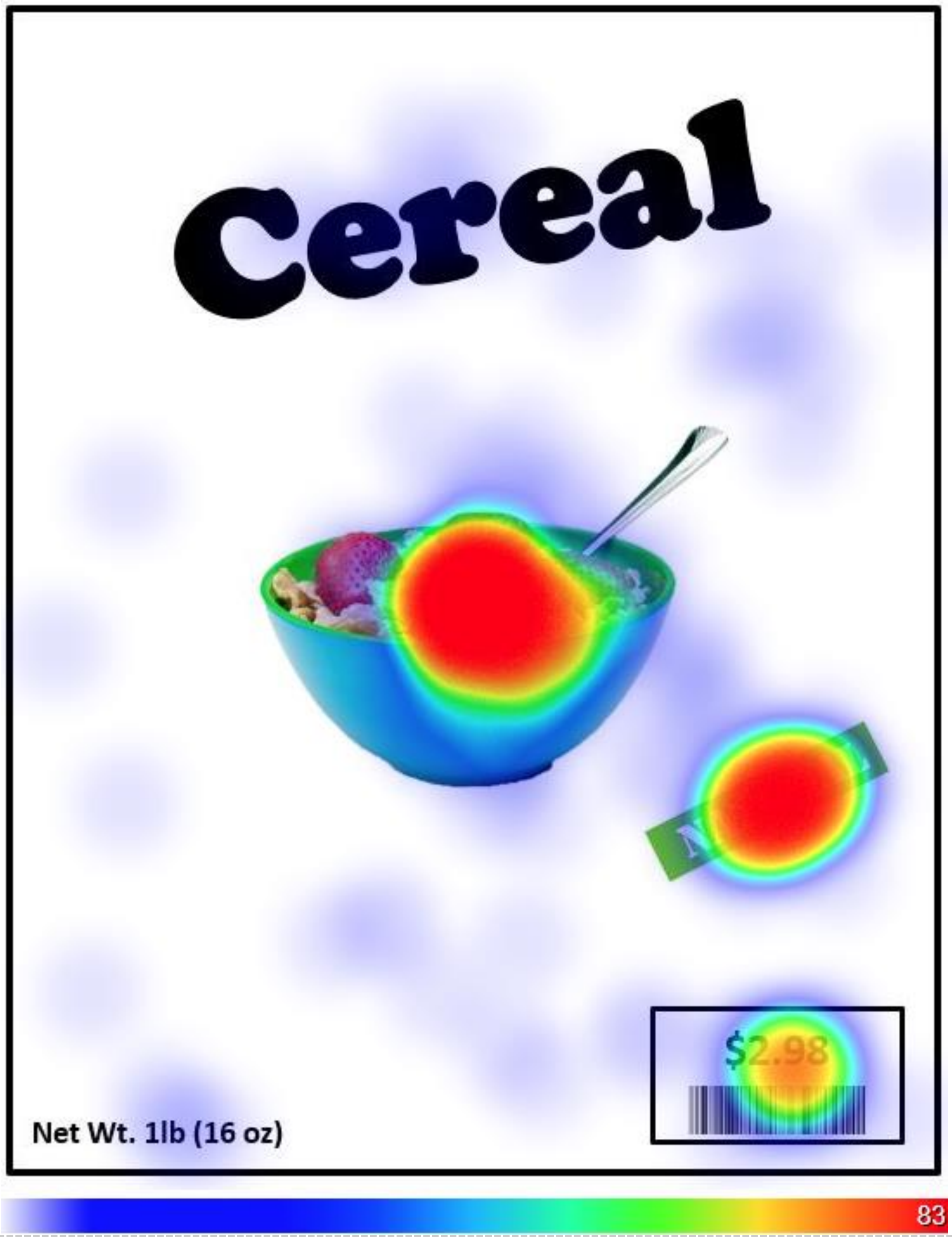
Cereal
$2.98
Net Wt. 1lb (16 oz)
83

Q41 Which part of the cereal box below is least attractive to you? *(please click or touch the image in the most attractive location)*

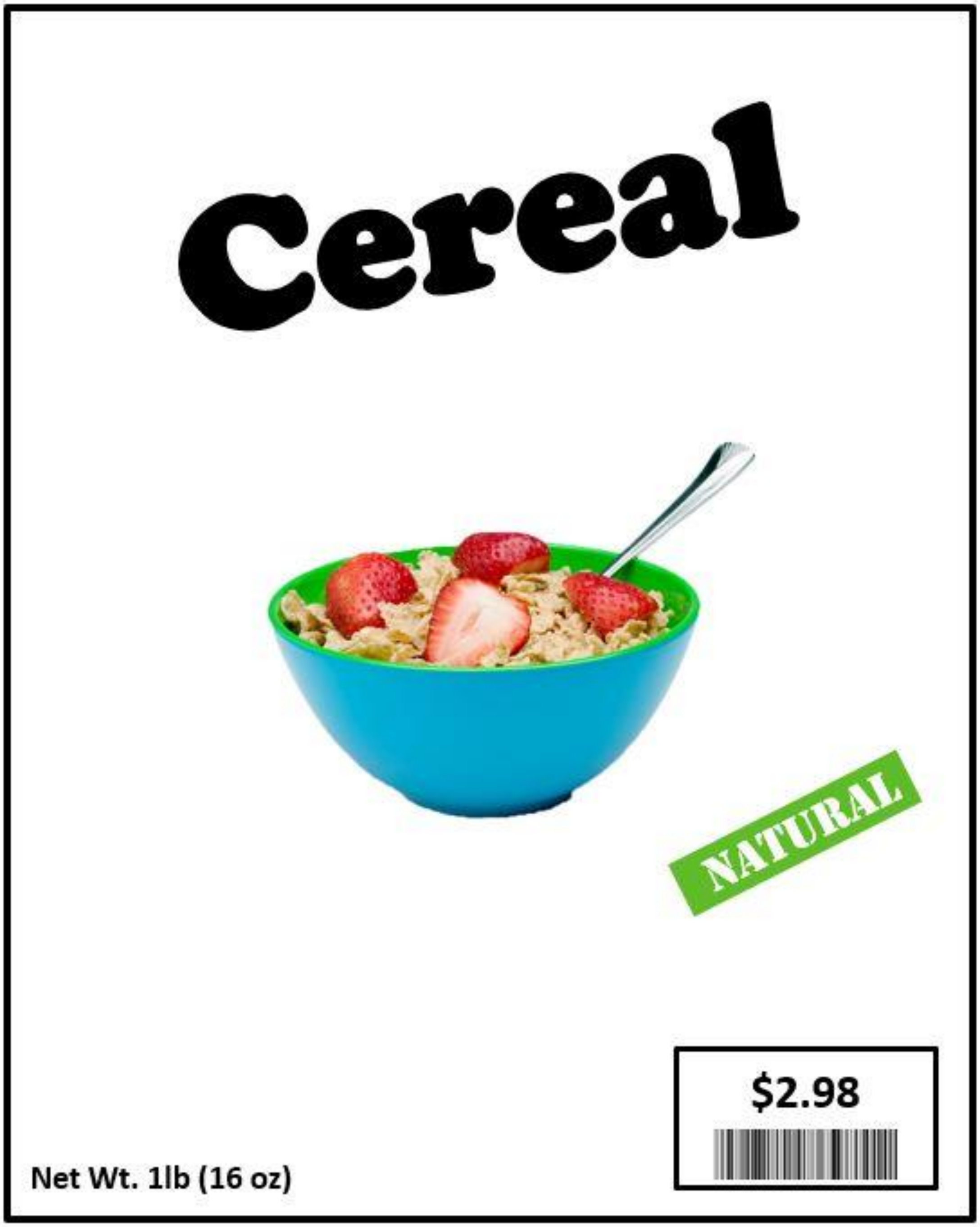

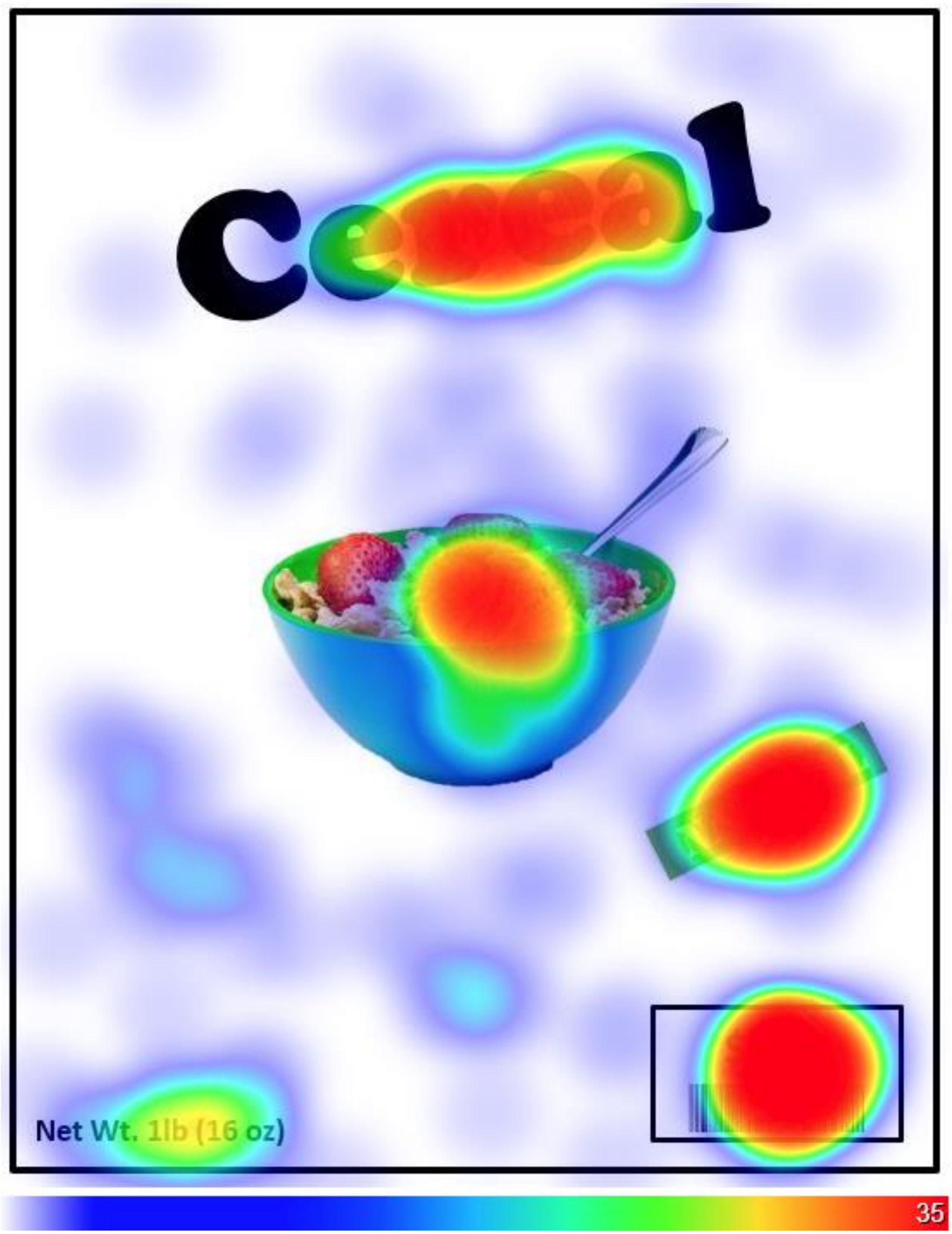

Q42 If you encountered this box of cereal in the grocery store, how likely would you be to purchase it? *(0 = no chance of buying; 100 = 100% chance of buying)*

End of Block: cherrio3_natural_low

Start of Block: cereal4_natural_high

Q43 Which part of the cereal box below is <u>most</u> attractive to you? *(please click or touch the image in the most attractive location)*

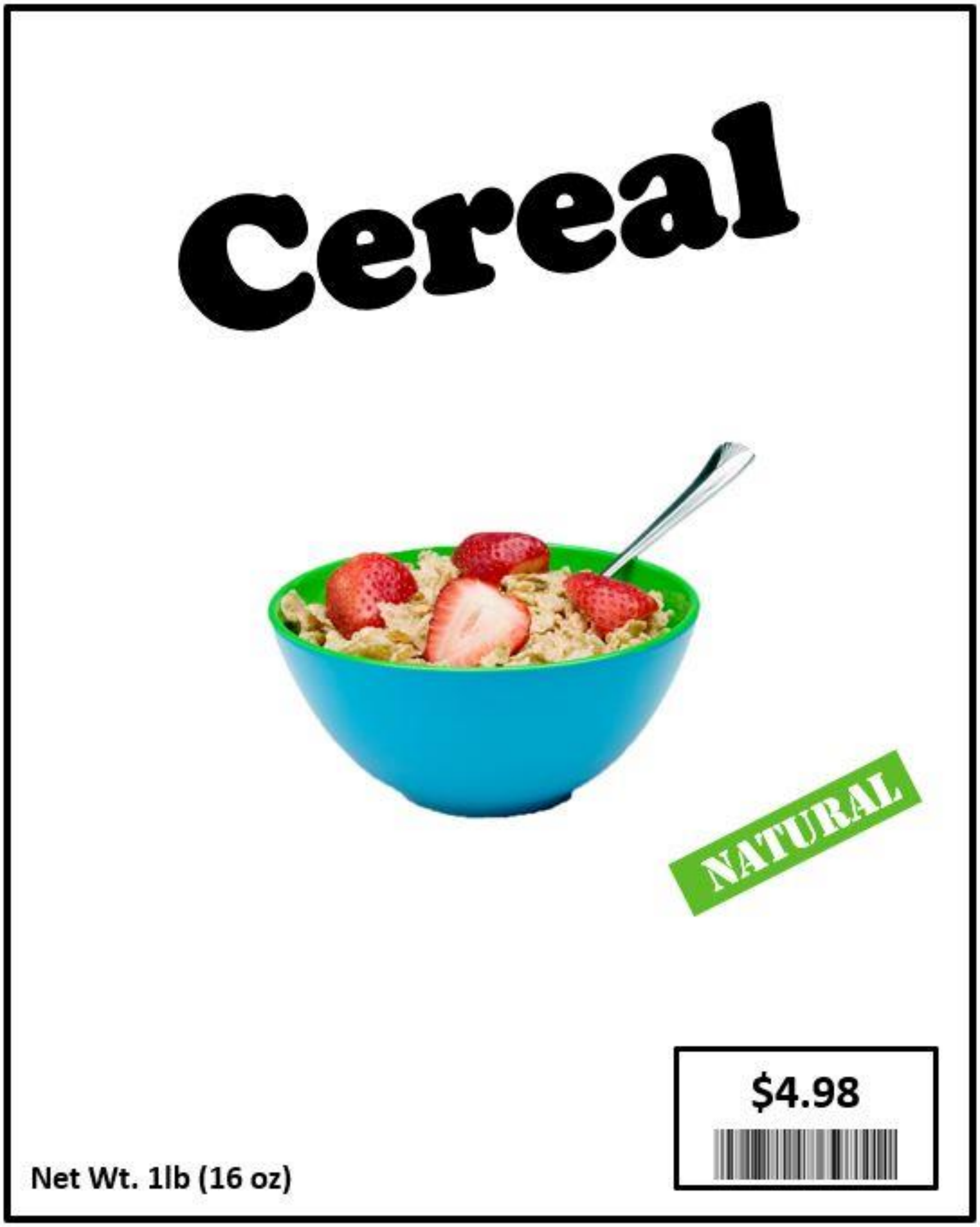

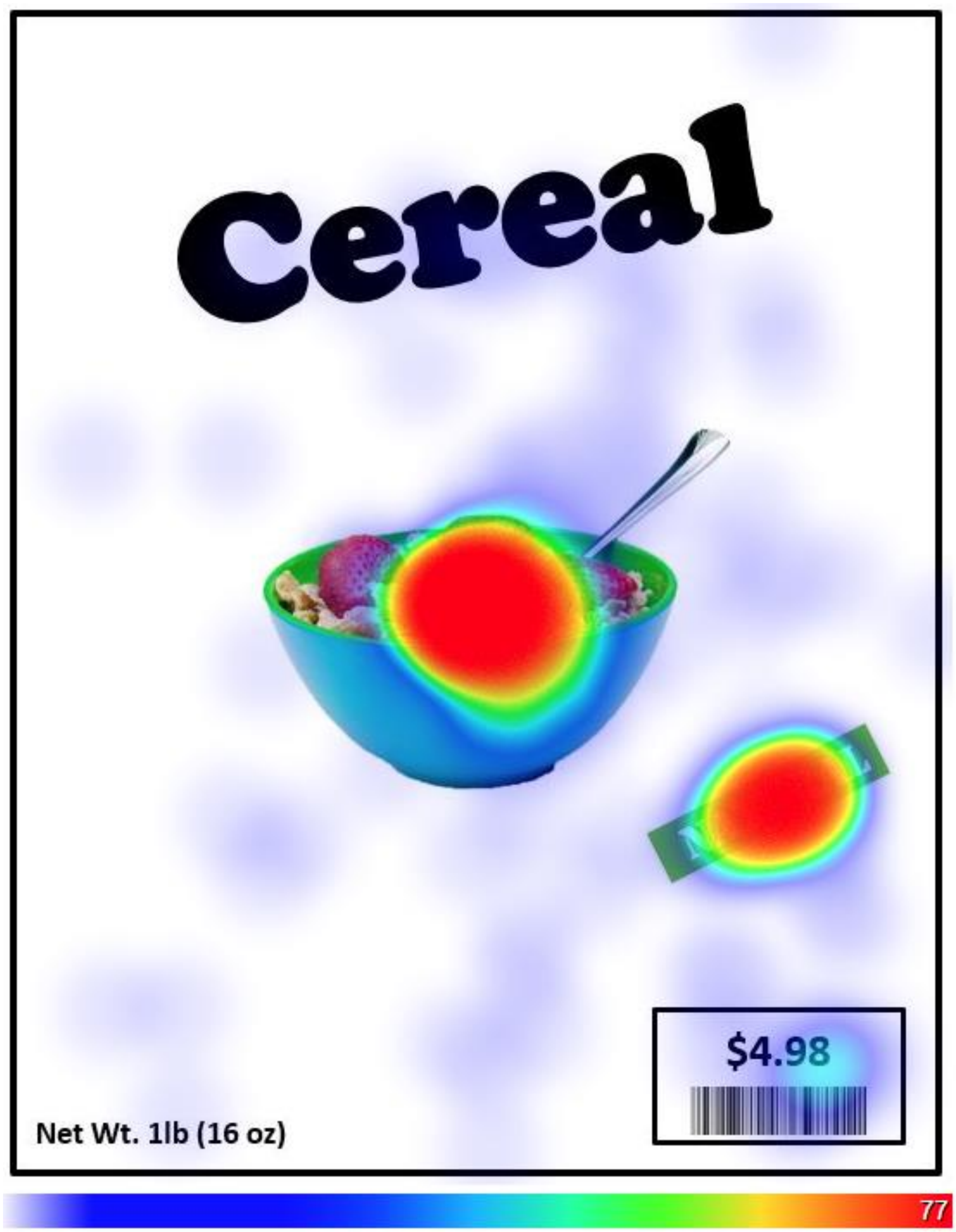
Cereal
$4.98
Net Wt. 1lb (16 oz)
77

Q44 Which part of the cereal box below is <u>least</u> attractive to you? *(please click or touch the image in the most attractive location)*

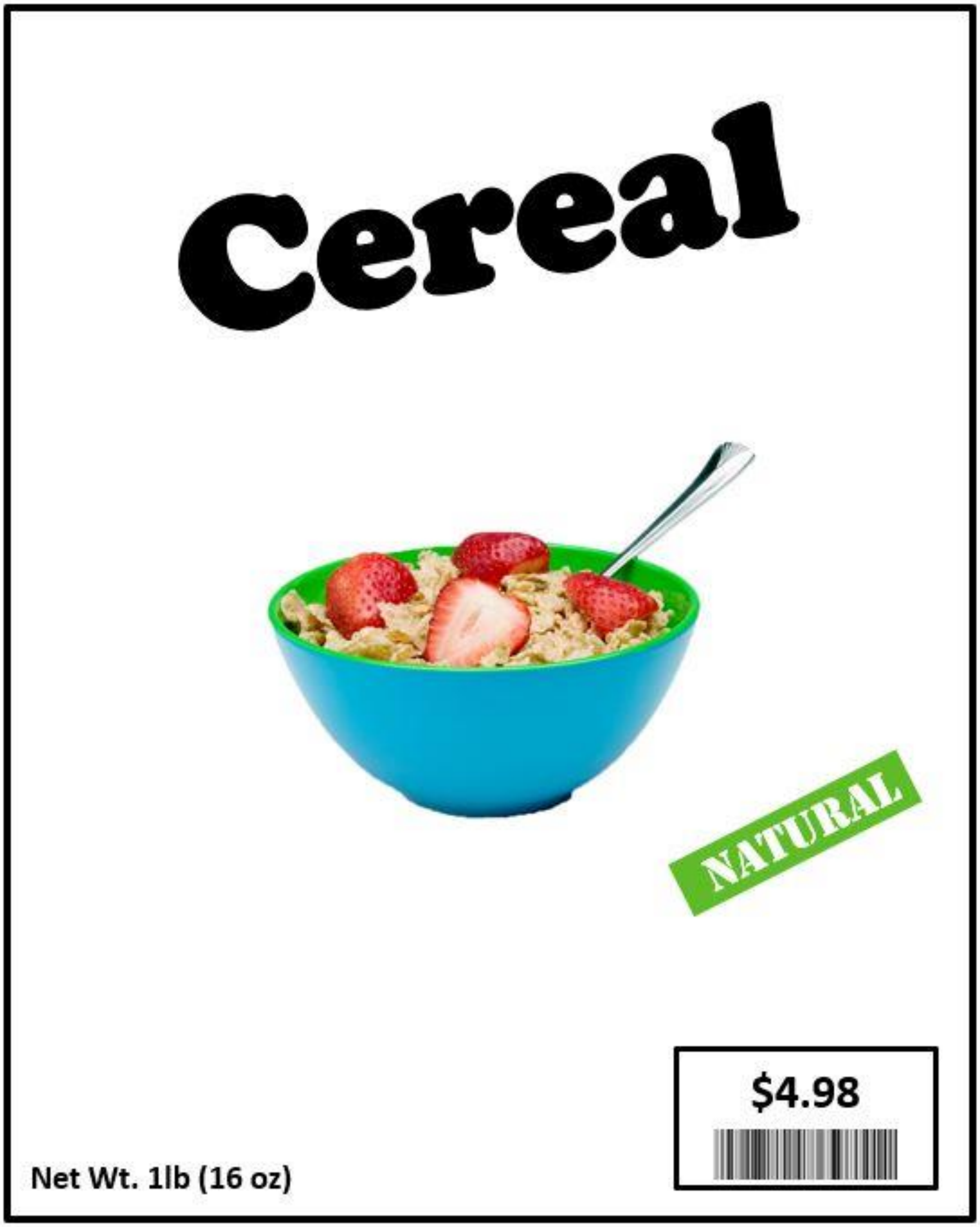

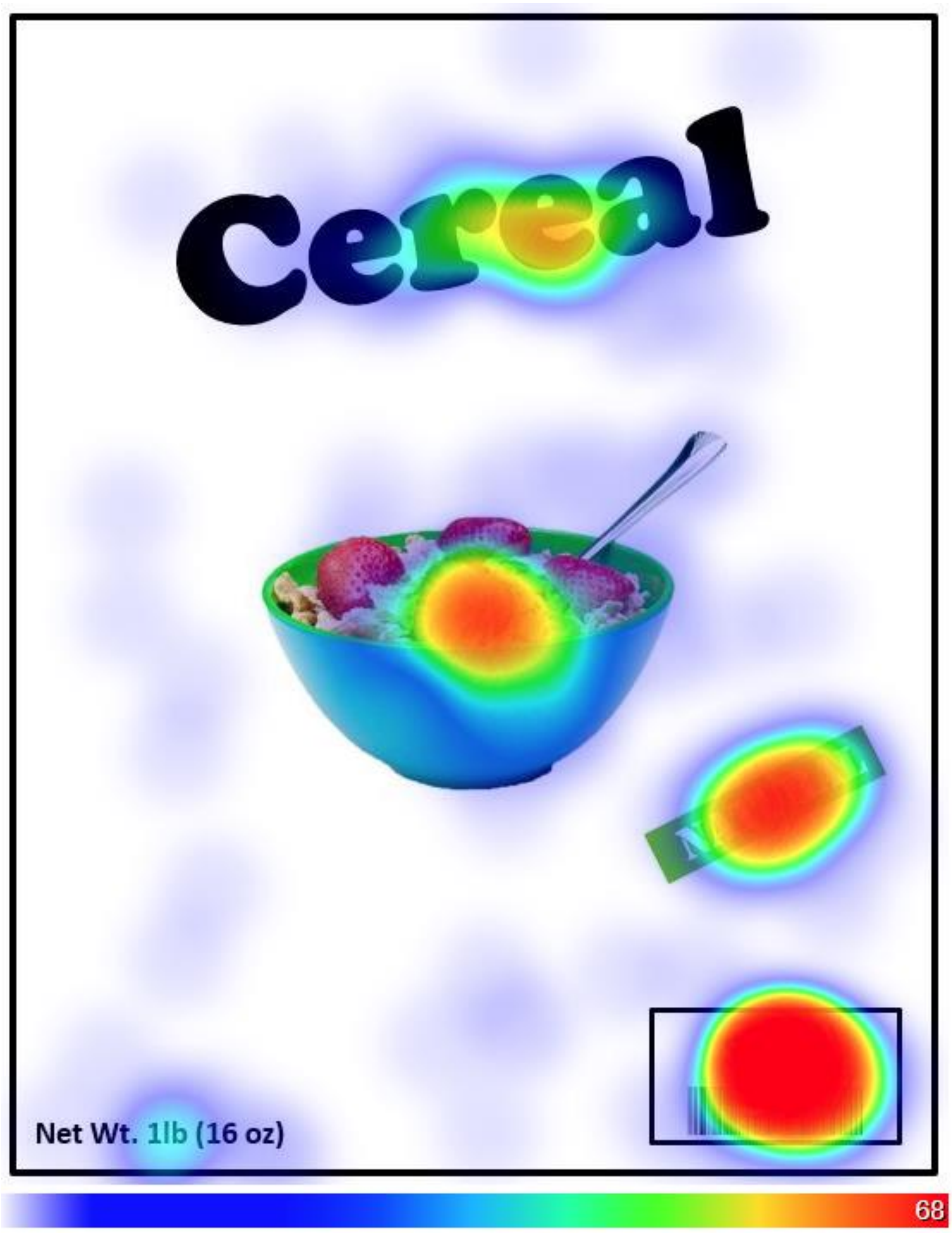

Q45 If you encountered this box of cereal in the grocery store, how likely would you be to purchase it? *(0 = no chance of buying; 100 = 100% chance of buying)*

End of Block: cereal4_natural_high

Start of Block: cereal5_health_low

Q46 Which part of the cereal box below is <u>most</u> attractive to you? *(please click or touch the image in the most attractive location)*

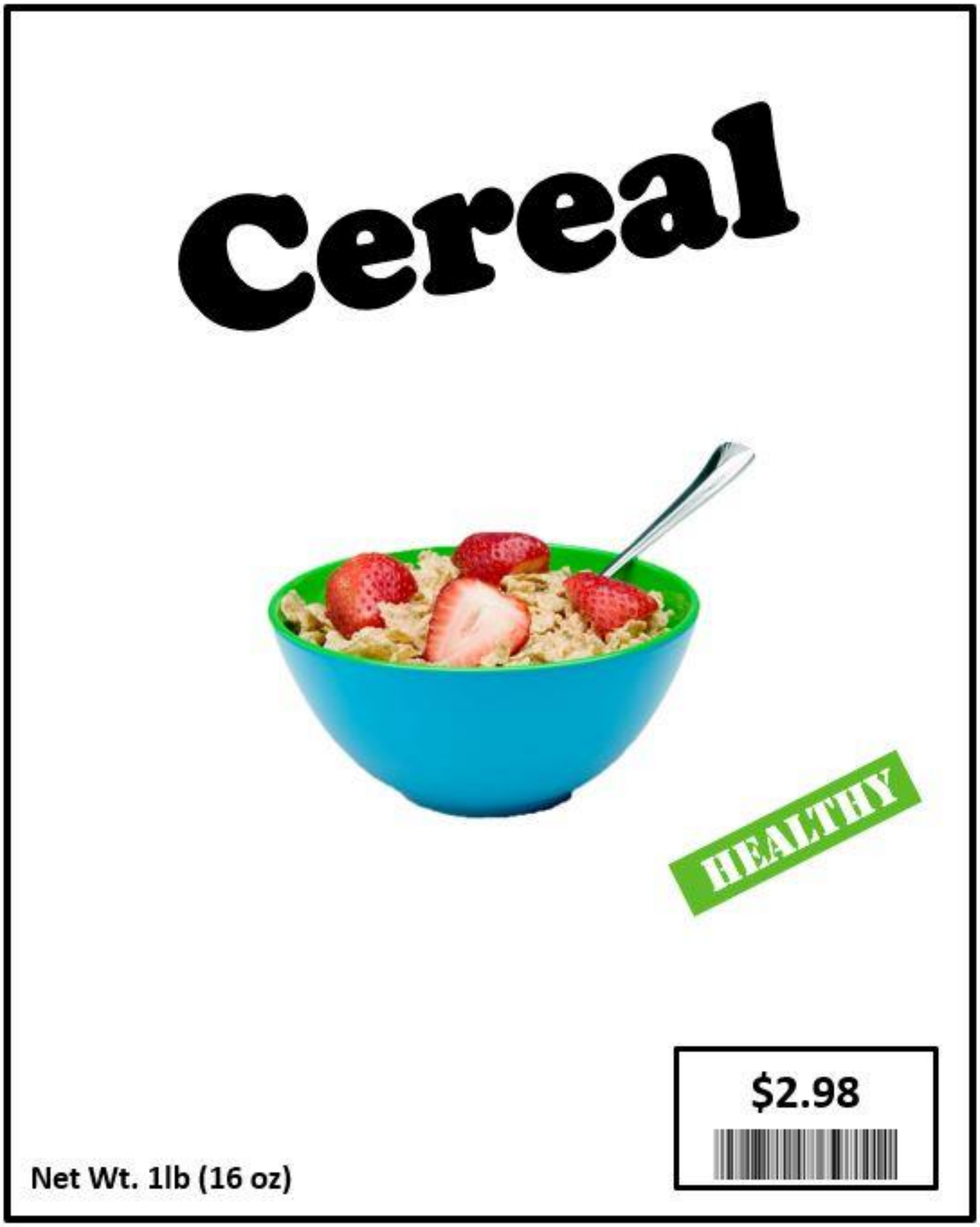

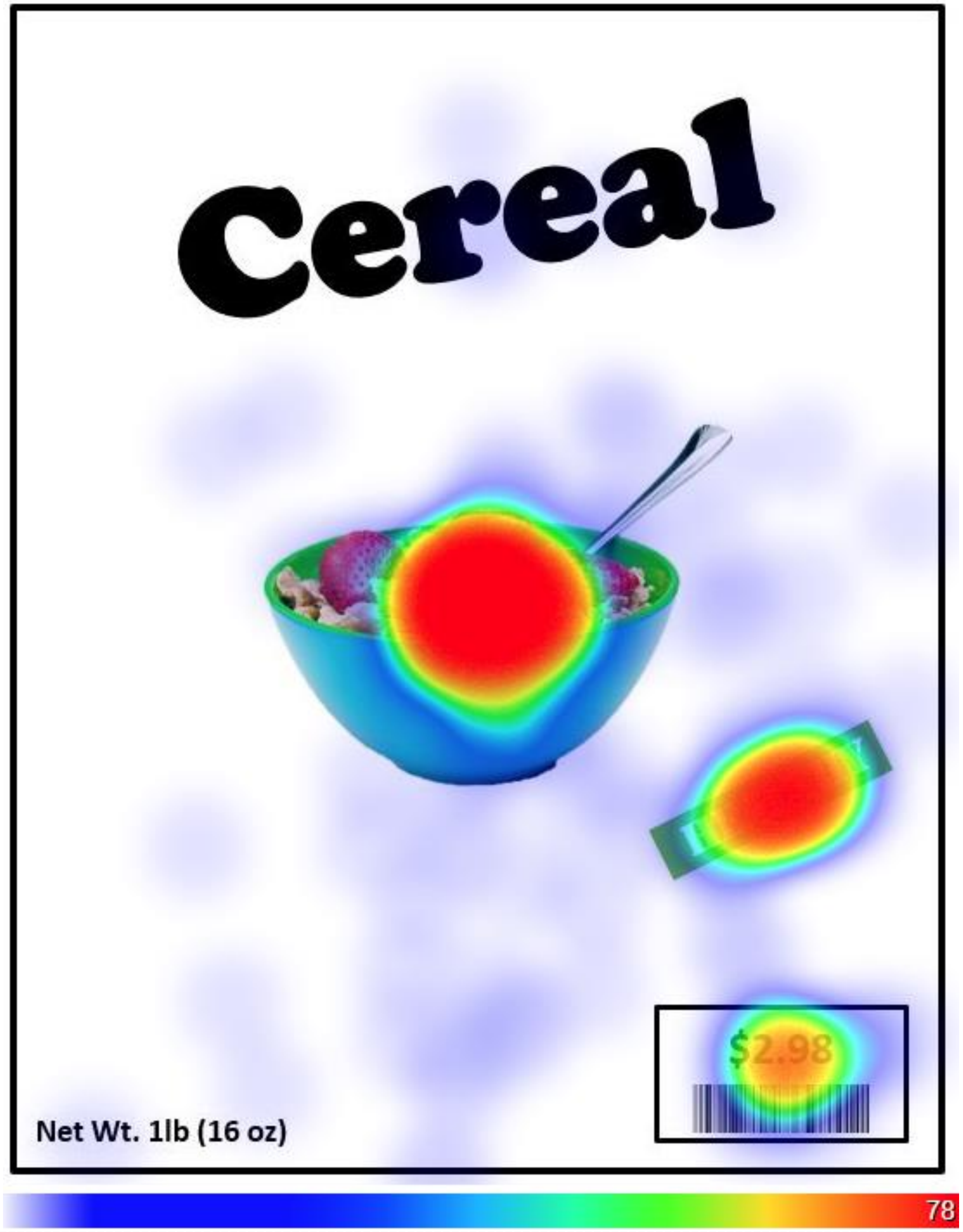
Cereal
$2.98
Net Wt. 1lb (16 oz)
78

Q47 Which part of the cereal box below is <u>least</u> attractive to you? *(please click or touch the image in the most attractive location)*

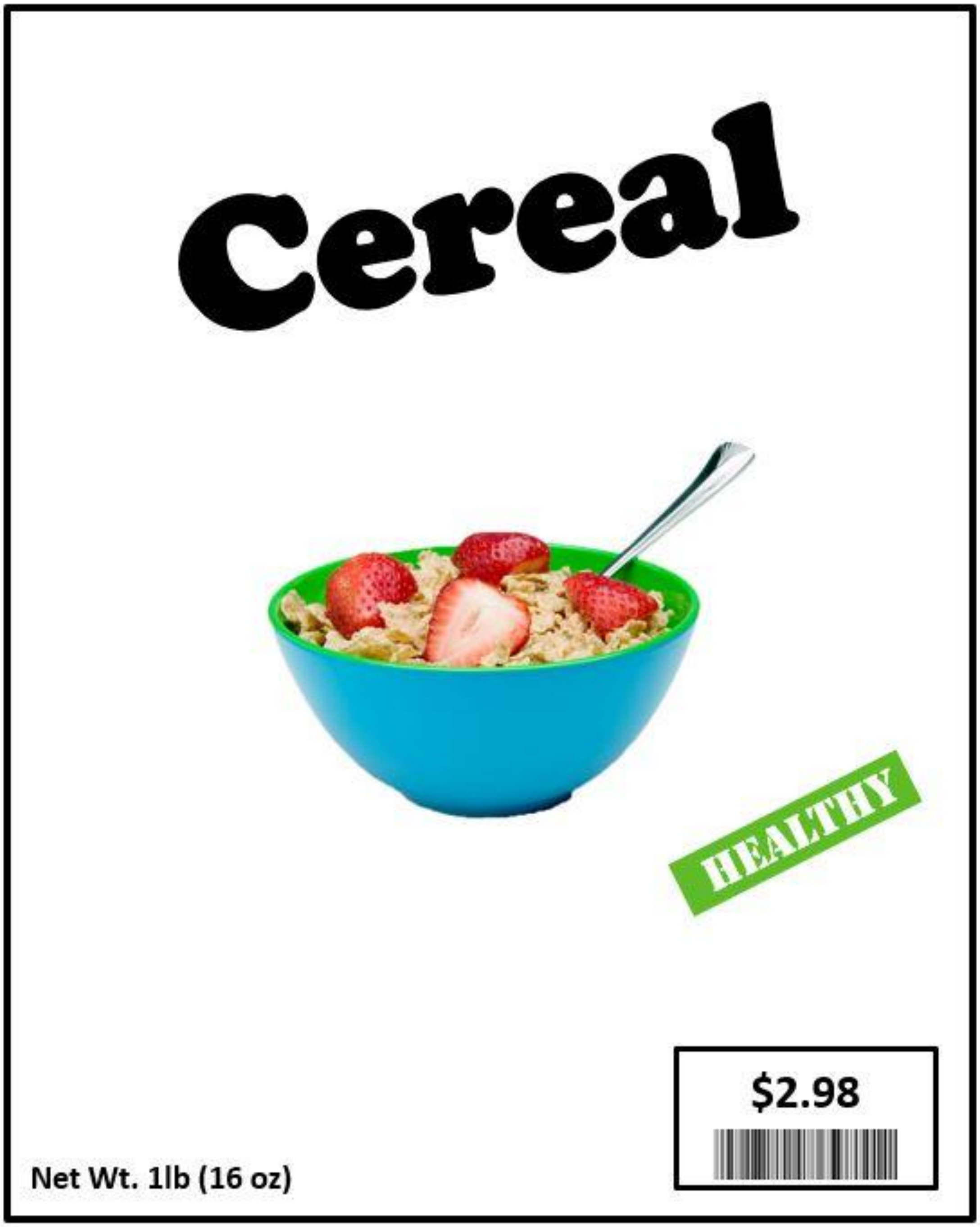

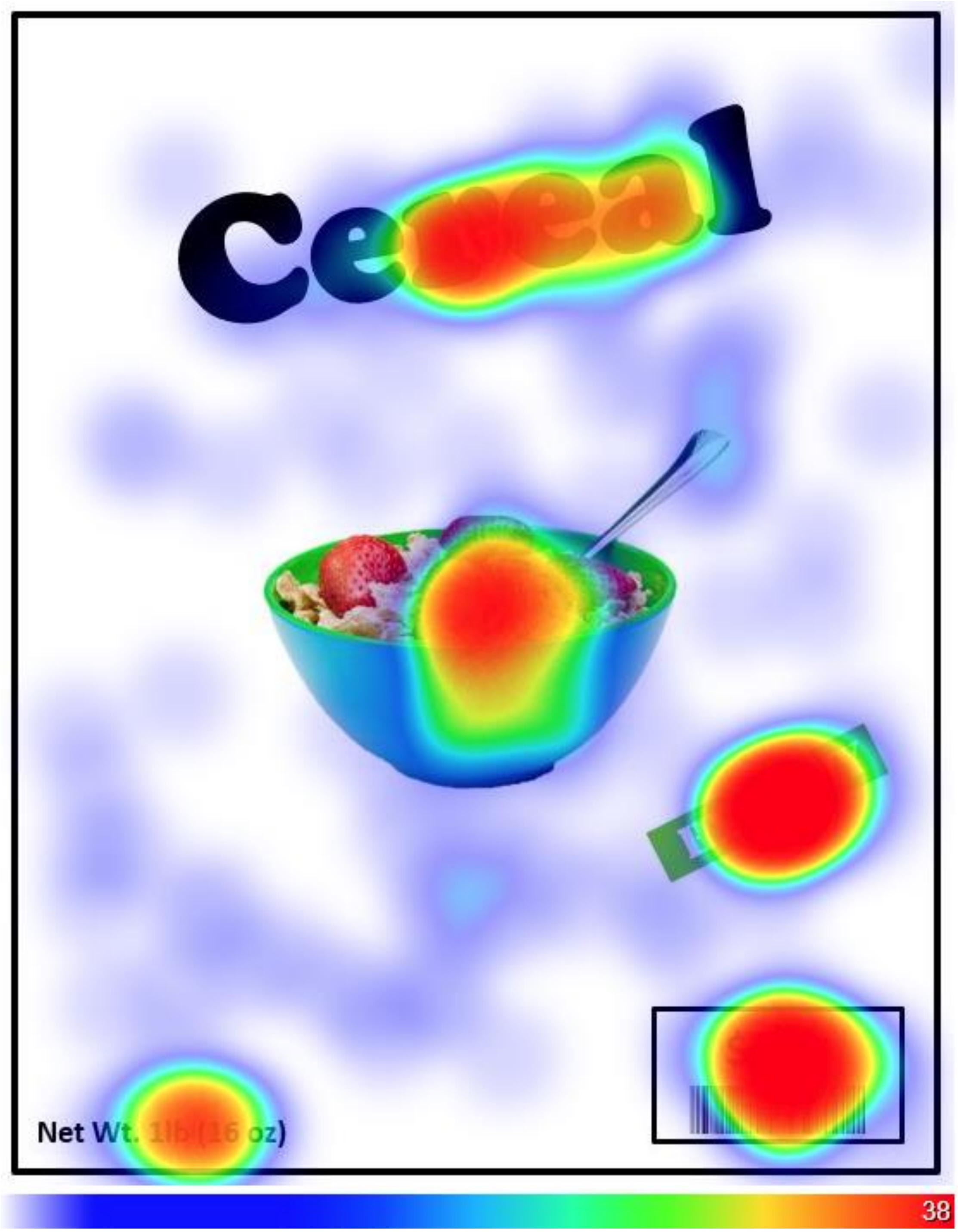

Q48 If you encountered this box of cereal in the grocery store, how likely would you be to purchase it? *(0 = no chance of buying; 100 = 100% chance of buying)*

End of Block: cereal5_health_low

Start of Block: cereal6_health_hi

Q49 Which part of the cereal box below is <u>most</u> attractive to you? *(please click or touch the image in the most attractive location)*

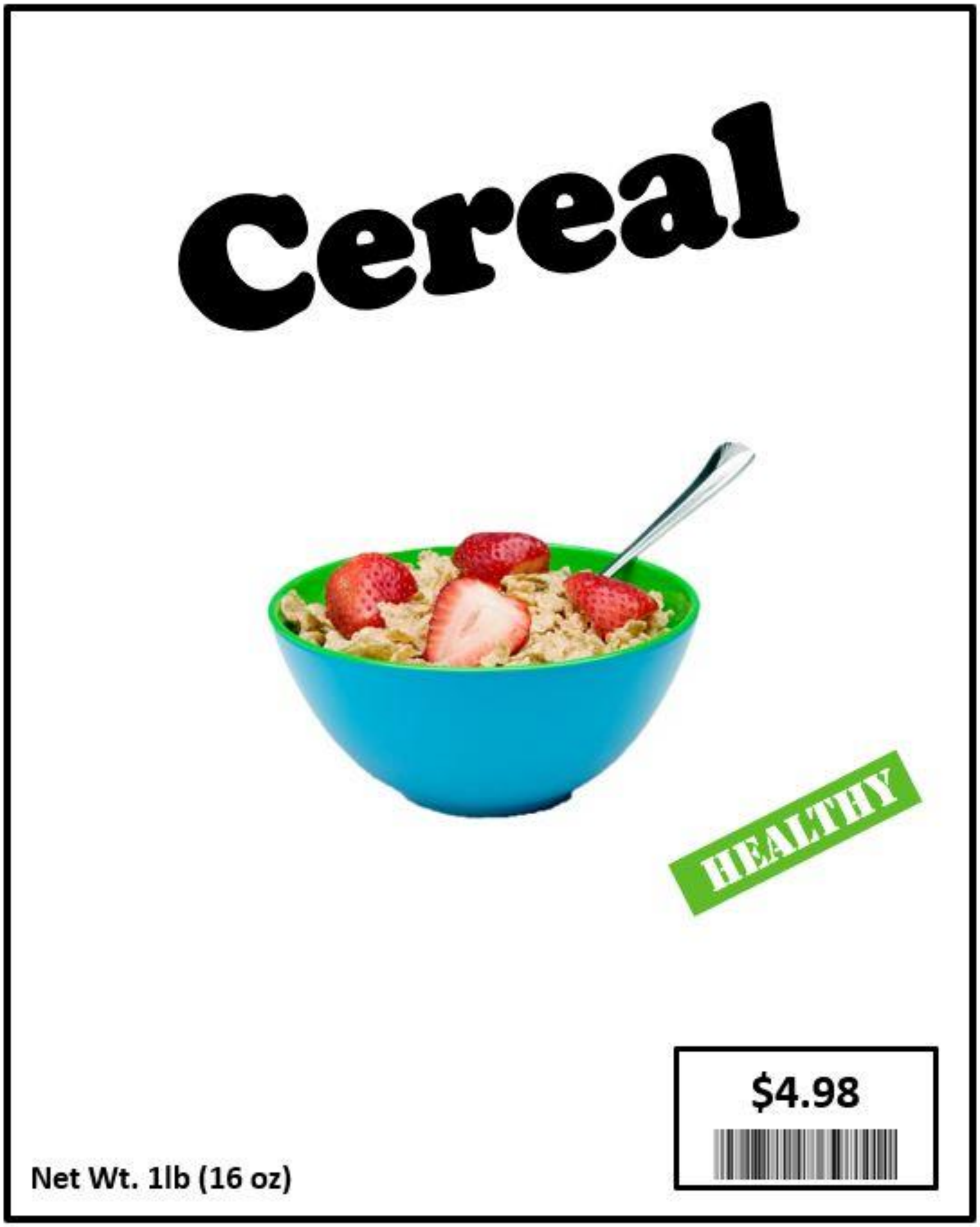

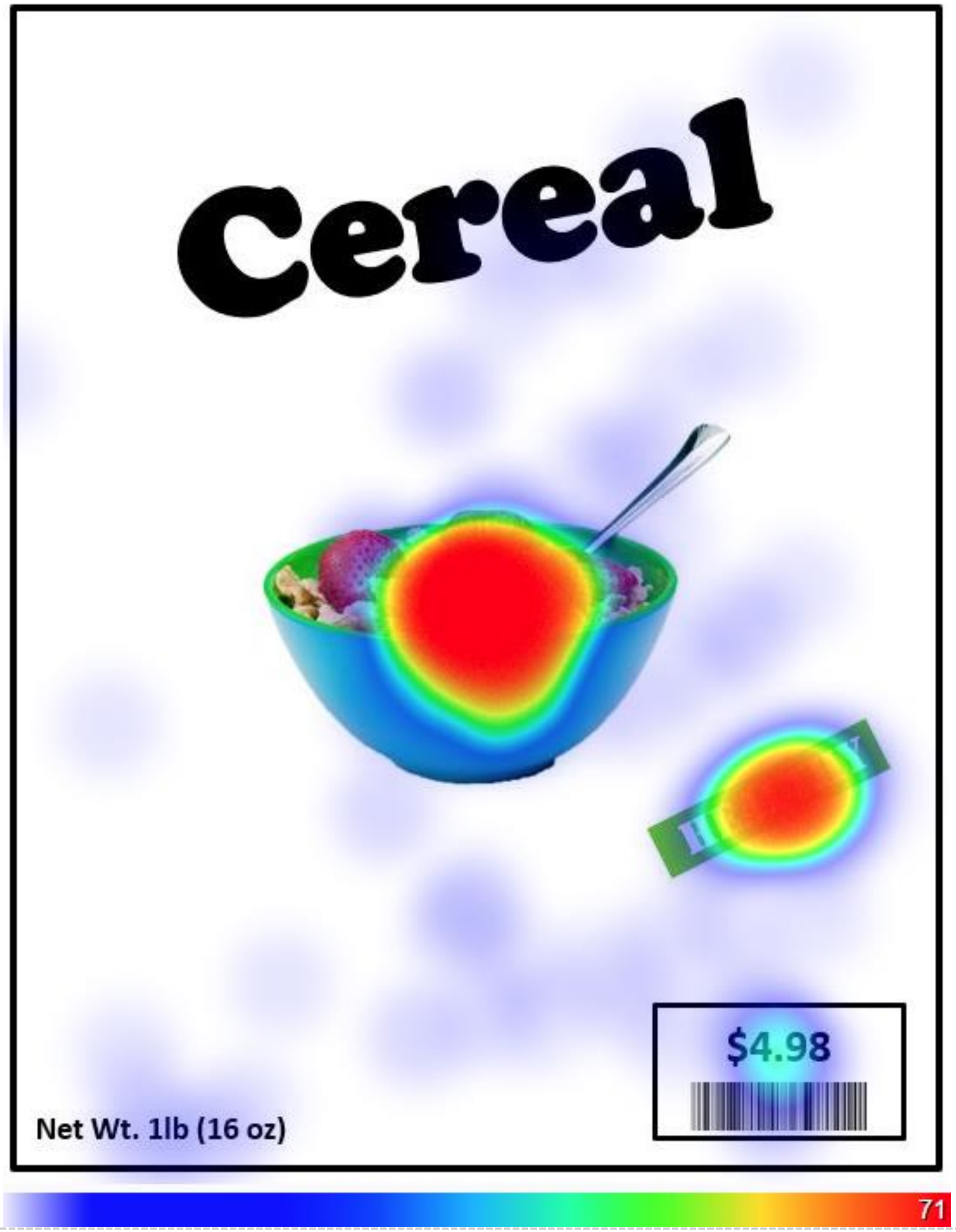
Cereal
$4.98
Net Wt. 1lb (16 oz)
71

Q50 Which part of the cereal box below is least attractive to you? *(please click or touch the image in the most attractive location)*

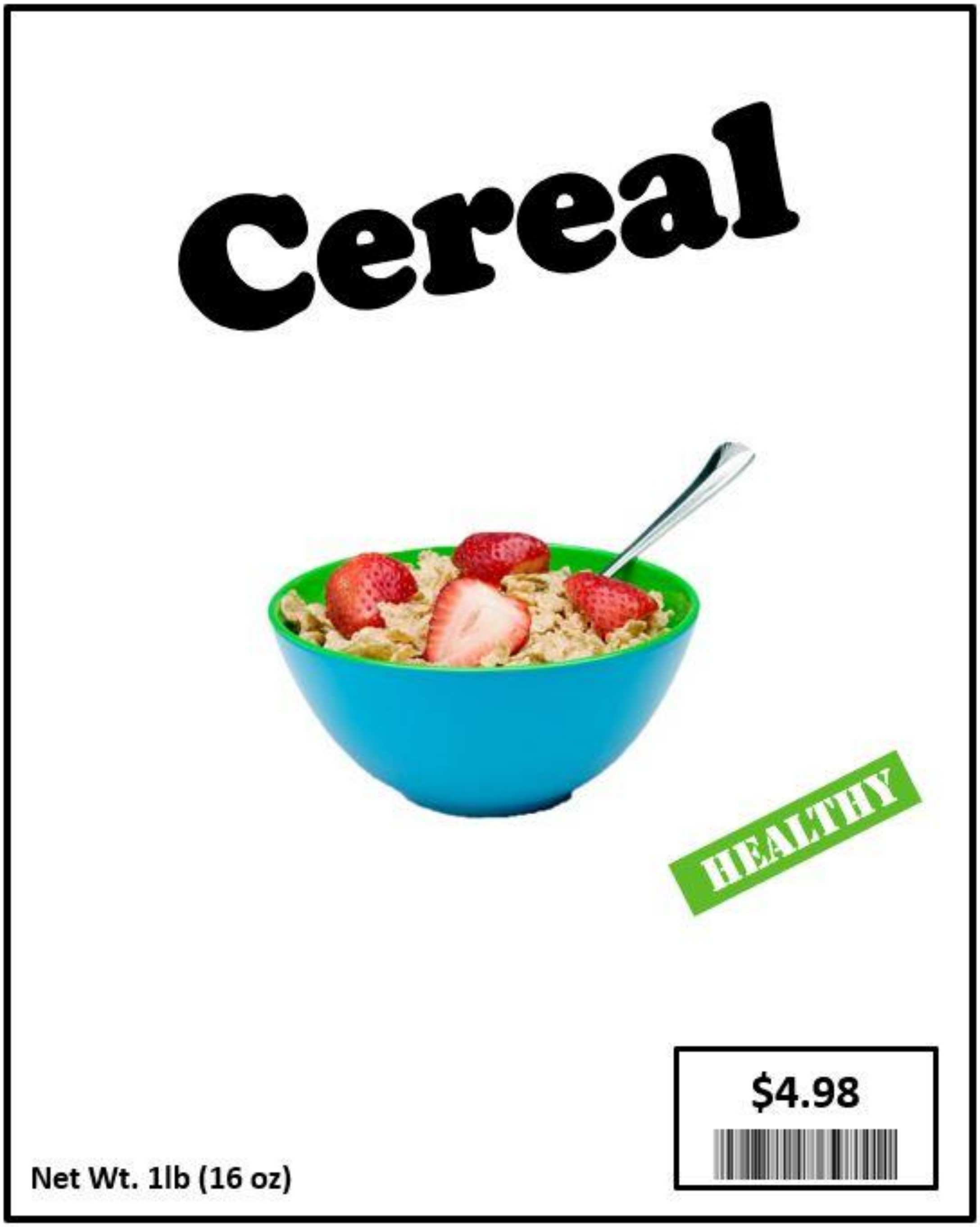

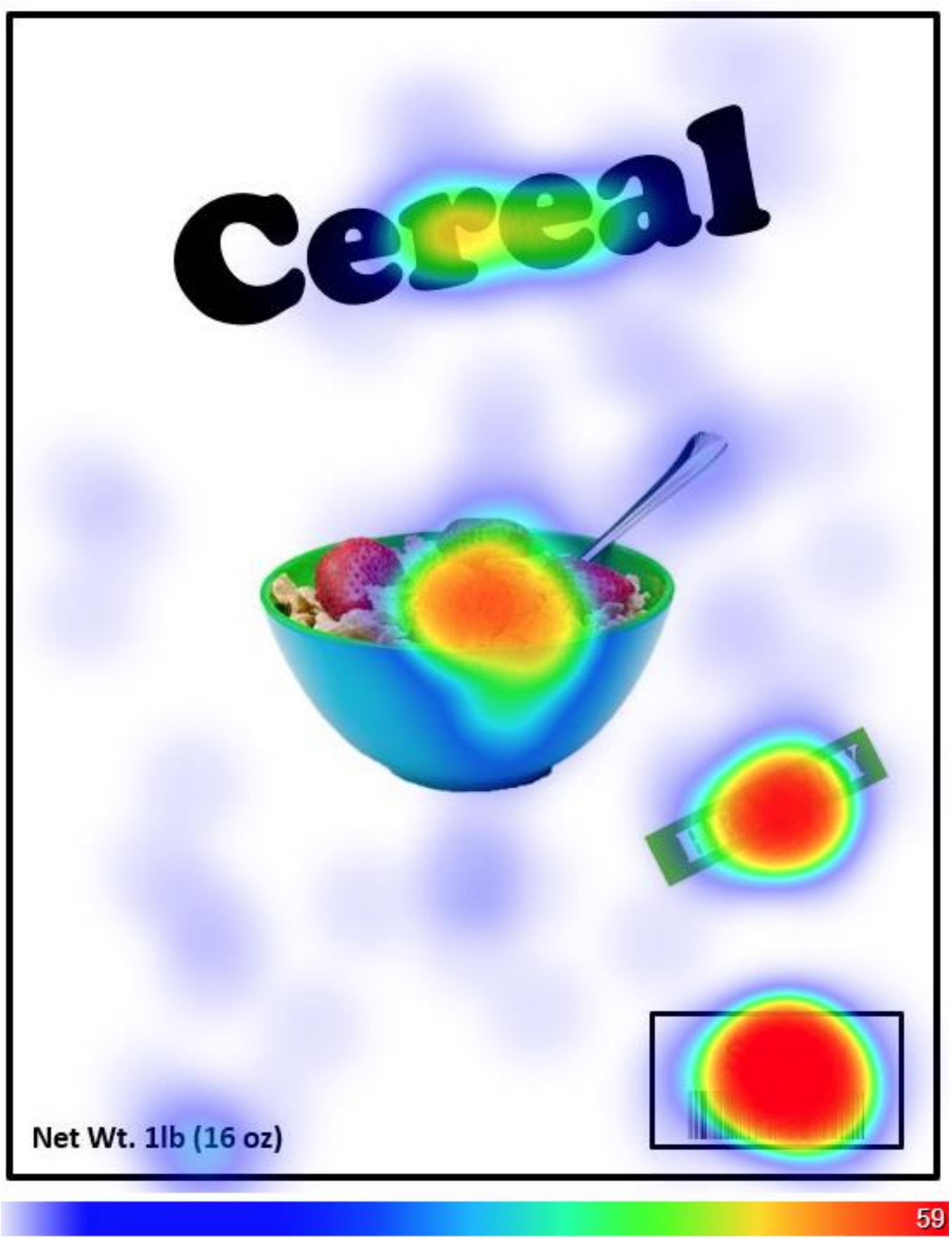

Q51 If you encountered this box of cereal in the grocery store, how likely would you be to purchase it? *(0 = no chance of buying; 100 = 100% chance of buying)*

End of Block: cereal6_health_hi

Start of Block: cereal7_organic_low

Q52 Which part of the cereal box below is <u>most</u> attractive to you? *(please click or touch the image in the most attractive location)*

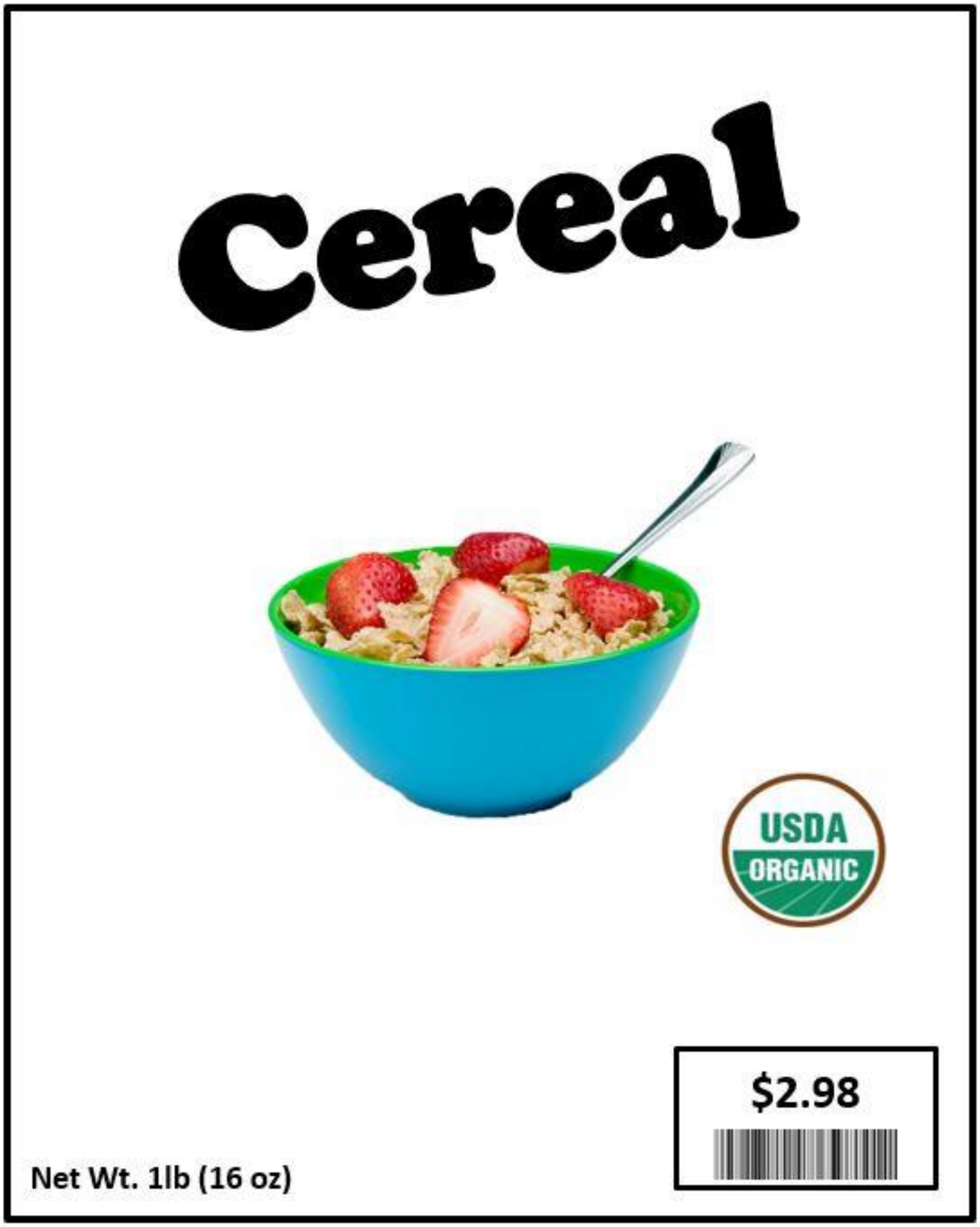

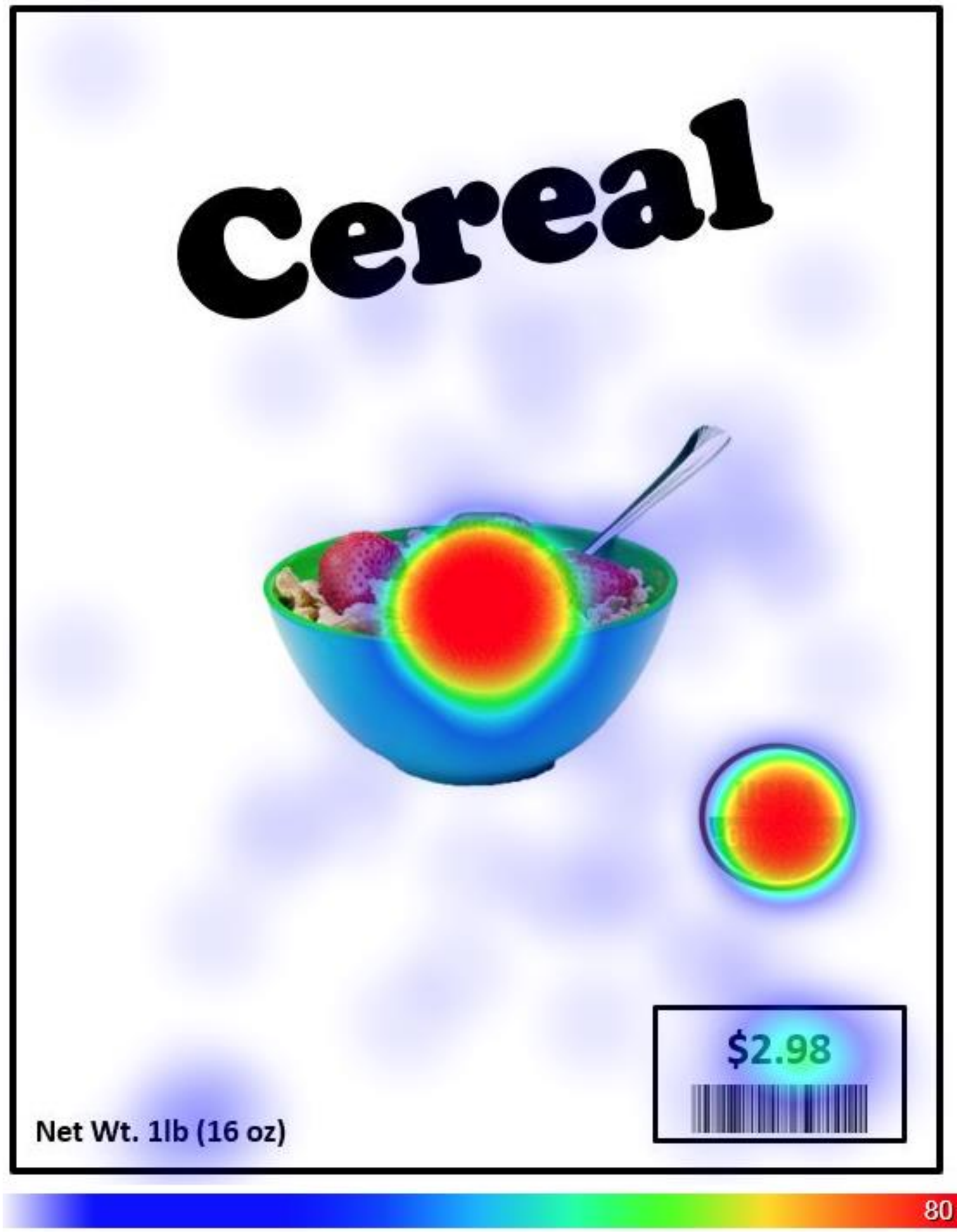
Cereal
$2.98
Net Wt. 1lb (16 oz)
80

Q53 Which part of the cereal box below is <u>least</u> attractive to you? *(please click or touch the image in the most attractive location)*

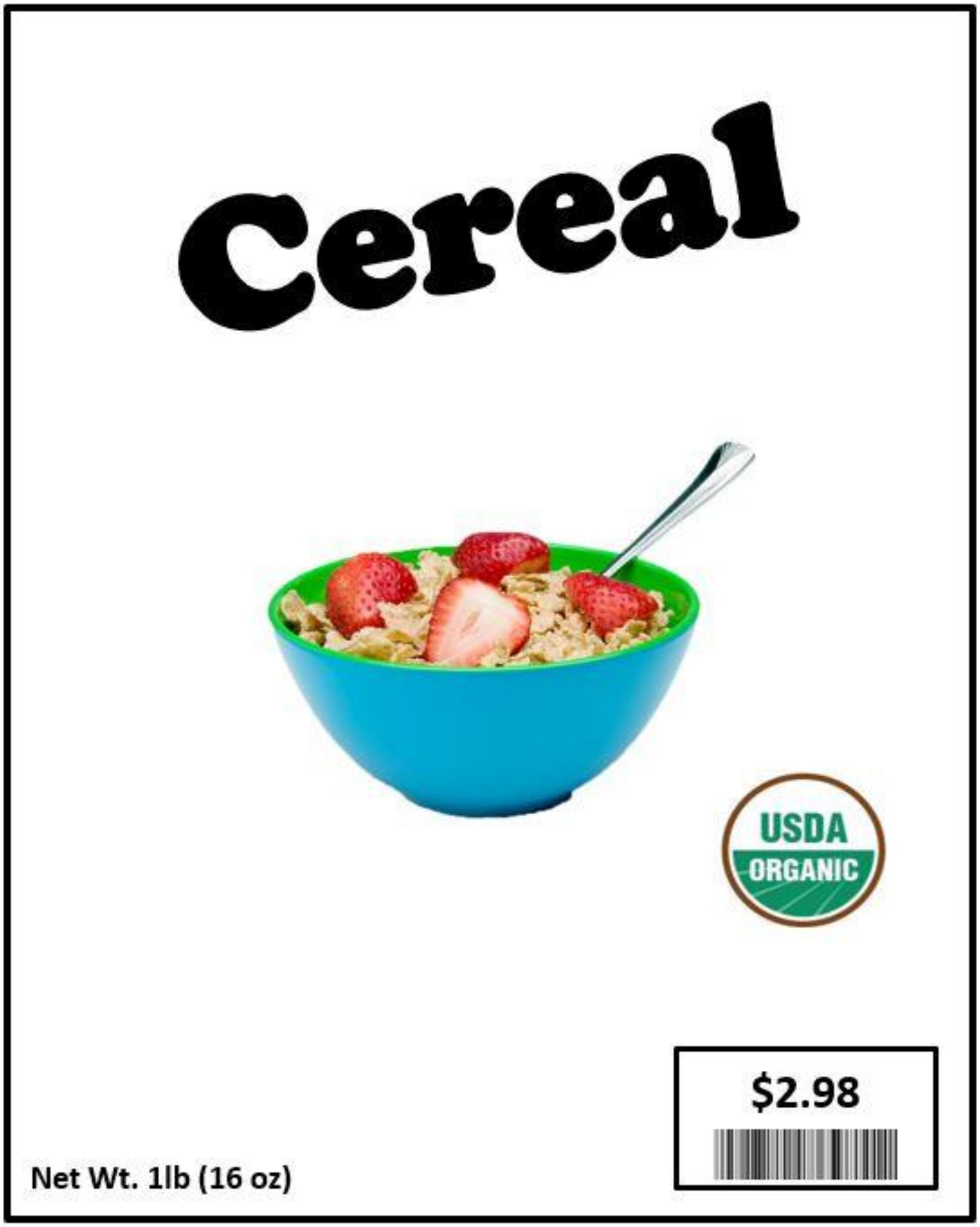

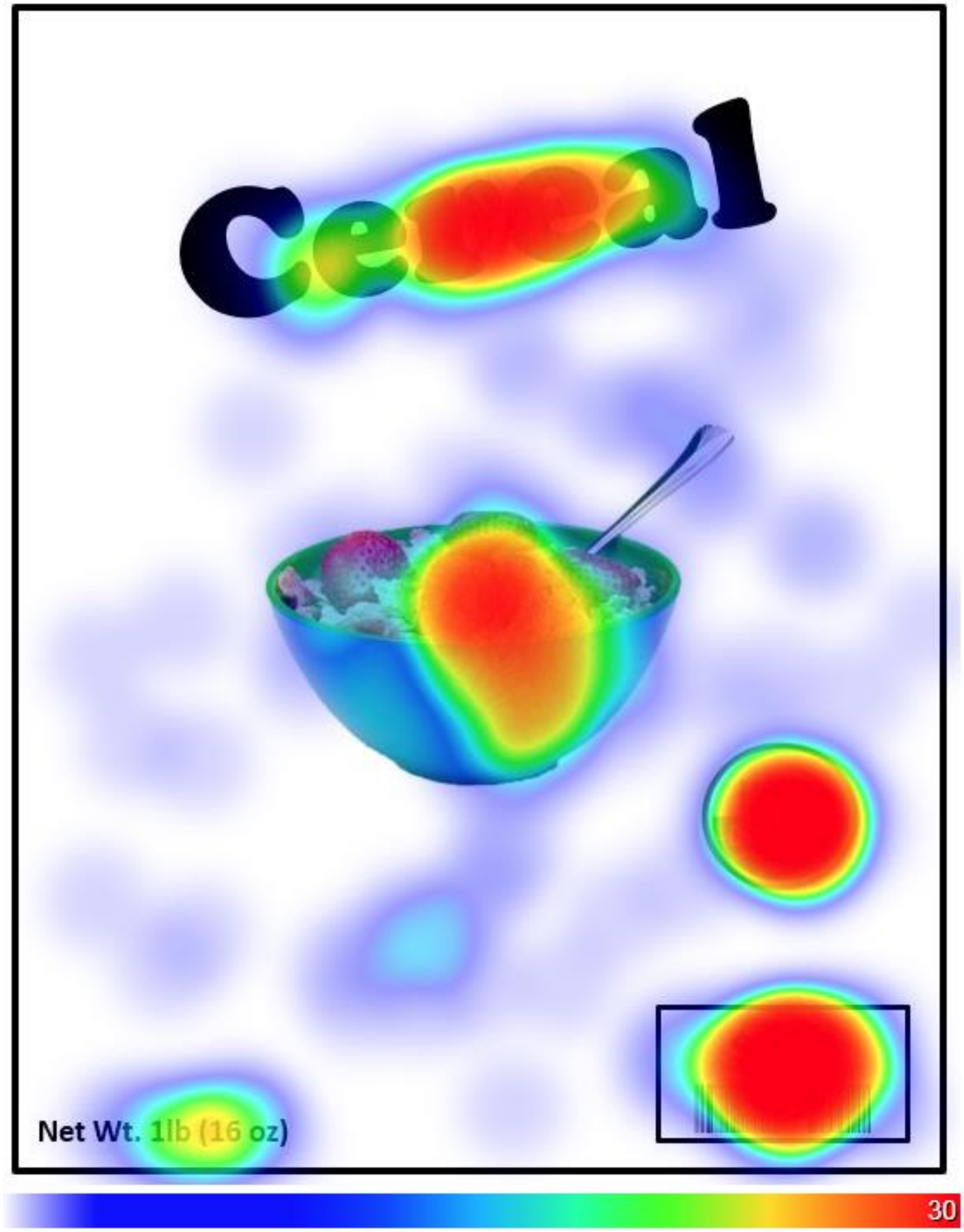

Q54 If you encountered this box of cereal in the grocery store, how likely would you be to purchase it? *(0 = no chance of buying; 100 = 100% chance of buying)*

End of Block: cereal7_organic_low

Start of Block: cereal8_organic_hi

Q55 Which part of the cereal box below is <u>most</u> attractive to you? *(please click or touch the image in the most attractive location)*

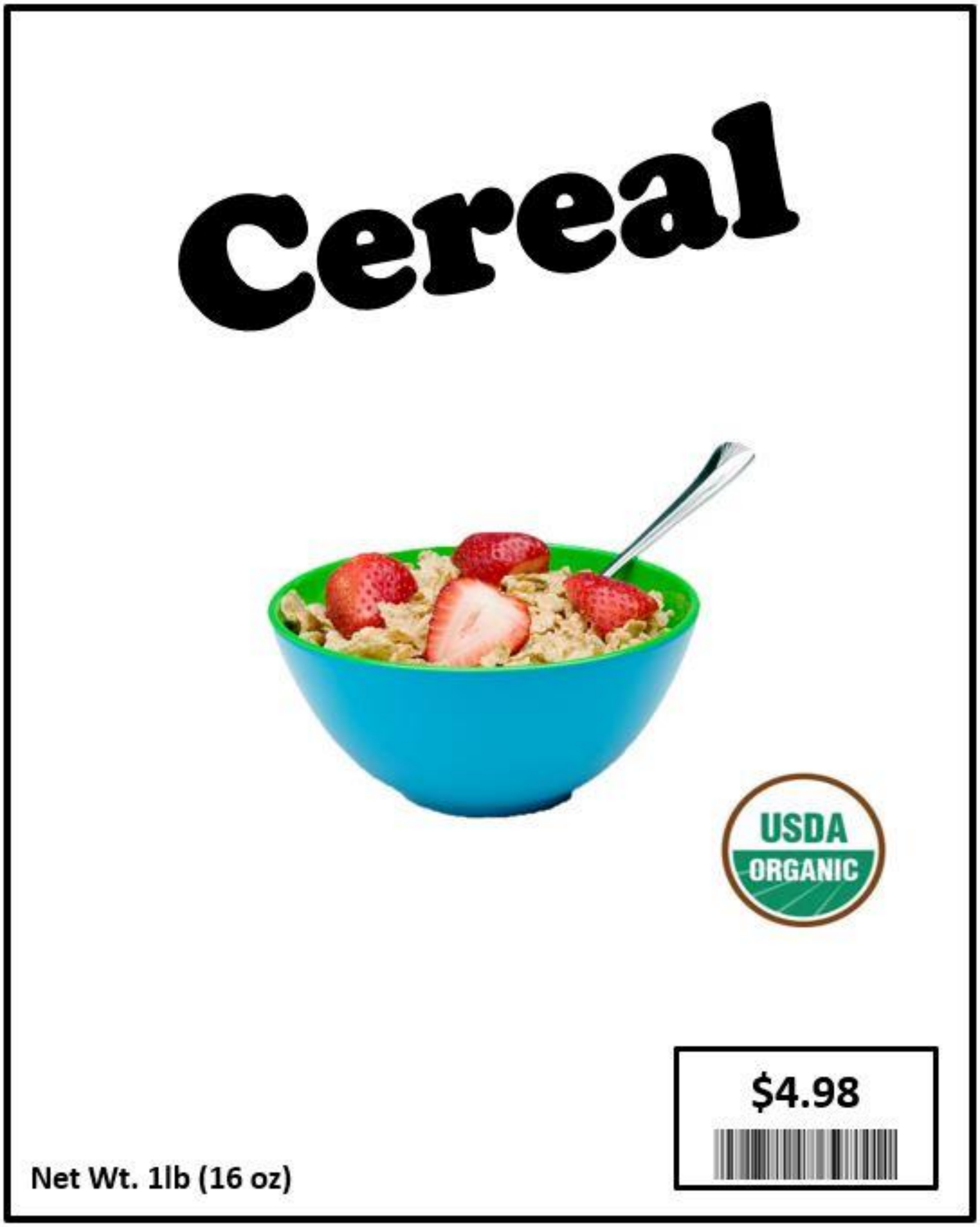

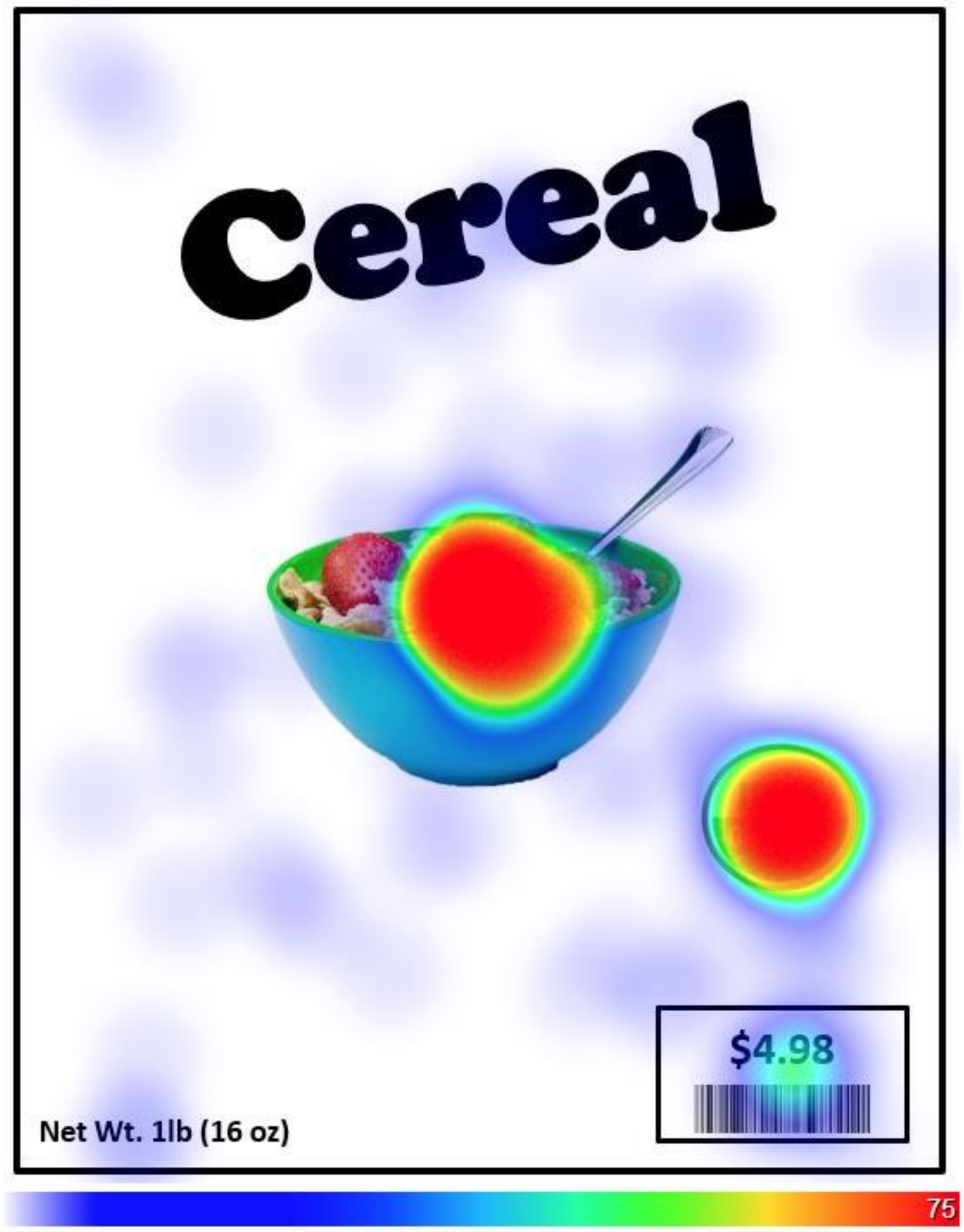
Cereal
$4.98
Net Wt. 1lb (16 oz)
75

Q56 Which part of the cereal box below is <u>least</u> attractive to you? *(please click or touch the image in the most attractive location)*

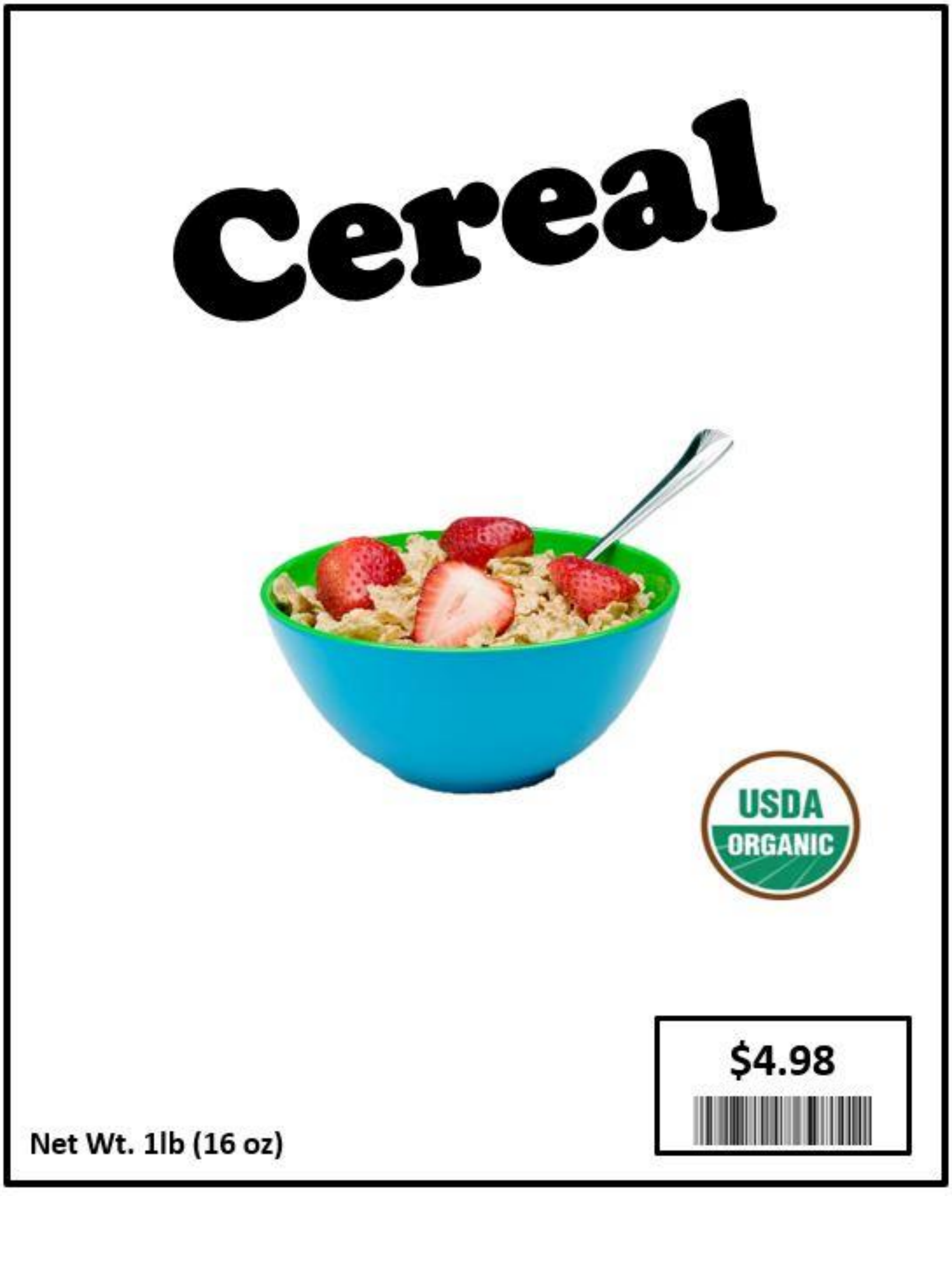

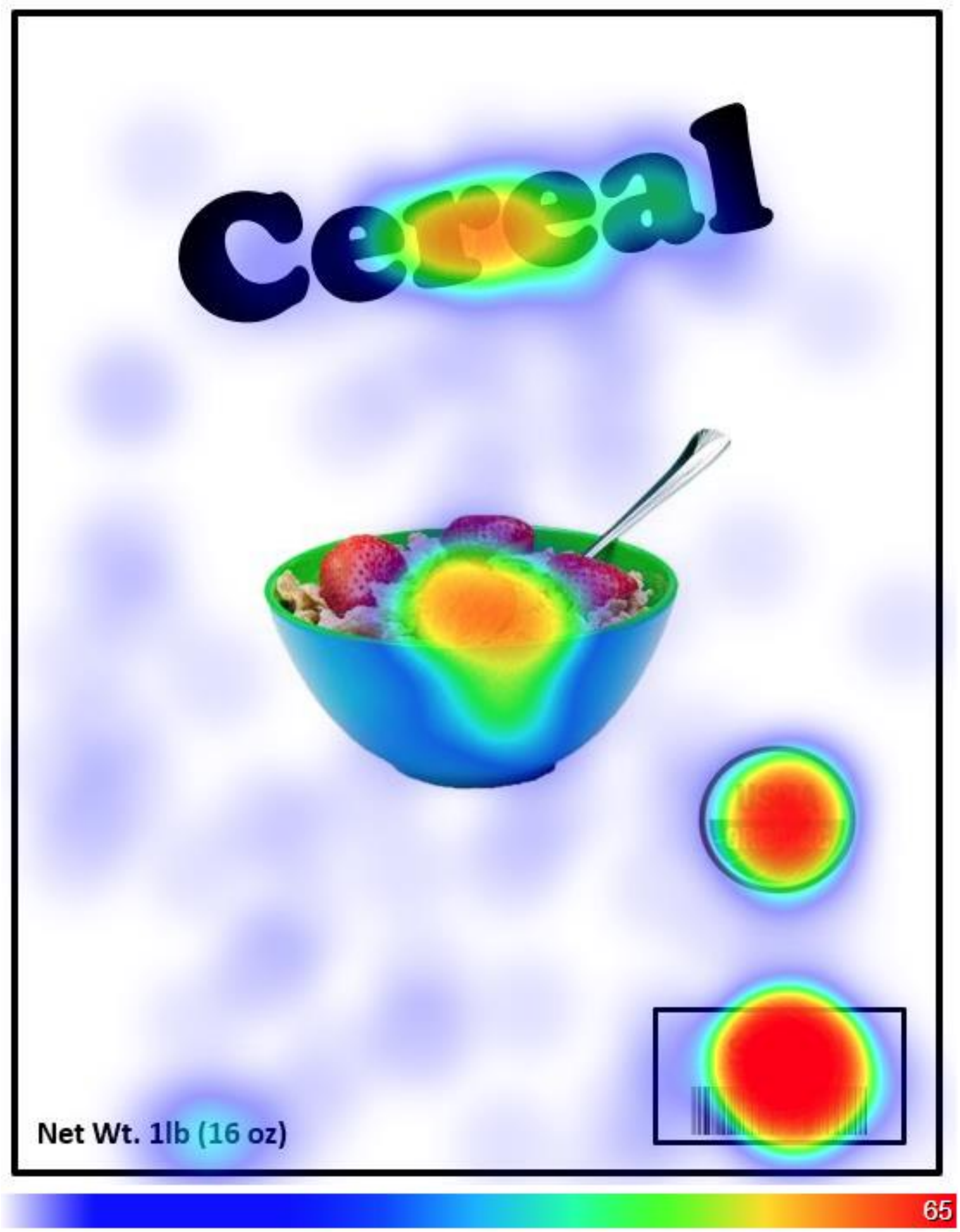

Q57 If you encountered this box of cereal in the grocery store, how likely would you be to purchase it? *(0 = no chance of buying; 100 = 100% chance of buying)*

End of Block: cereal8_organic_hi

Start of Block: cereal9_combo_low

Q58 Which part of the cereal box below is <u>most</u> attractive to you? *(please click or touch the image in the most attractive location)*

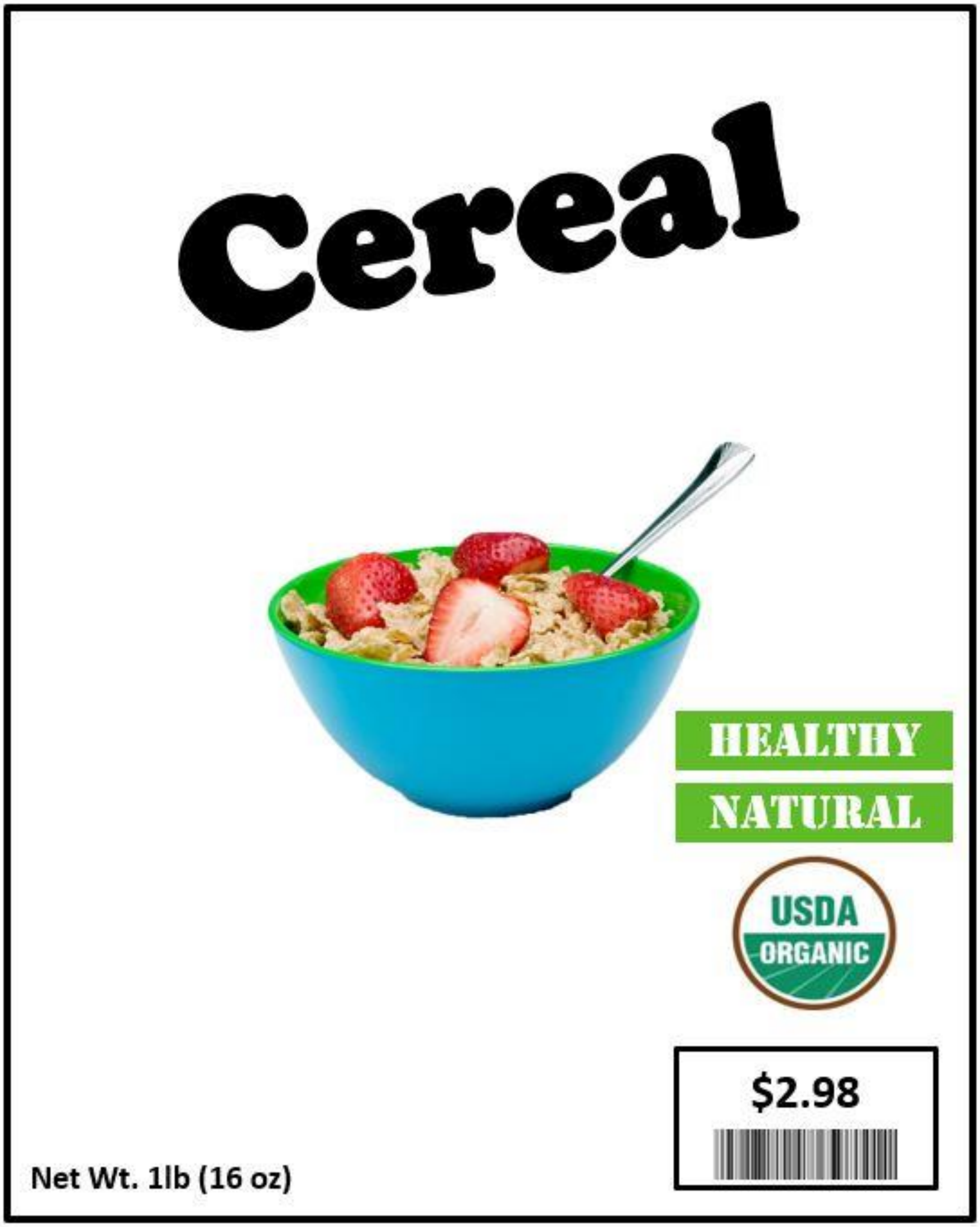

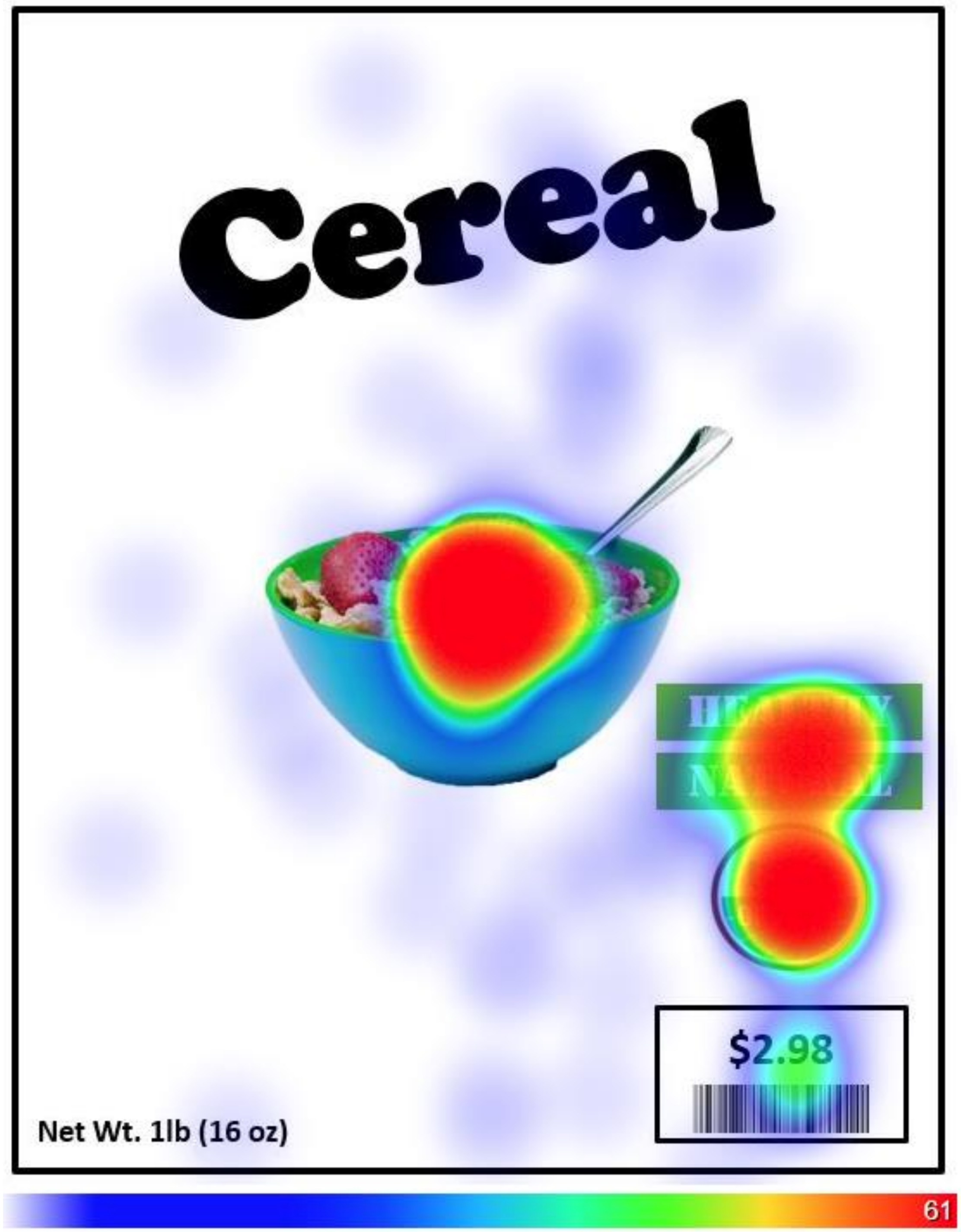
Cereal
$2.98
Net Wt. 1lb (16 oz)
61

Q59 Which part of the cereal box below is <u>least</u> attractive to you? *(please click or touch the image in the most attractive location)*

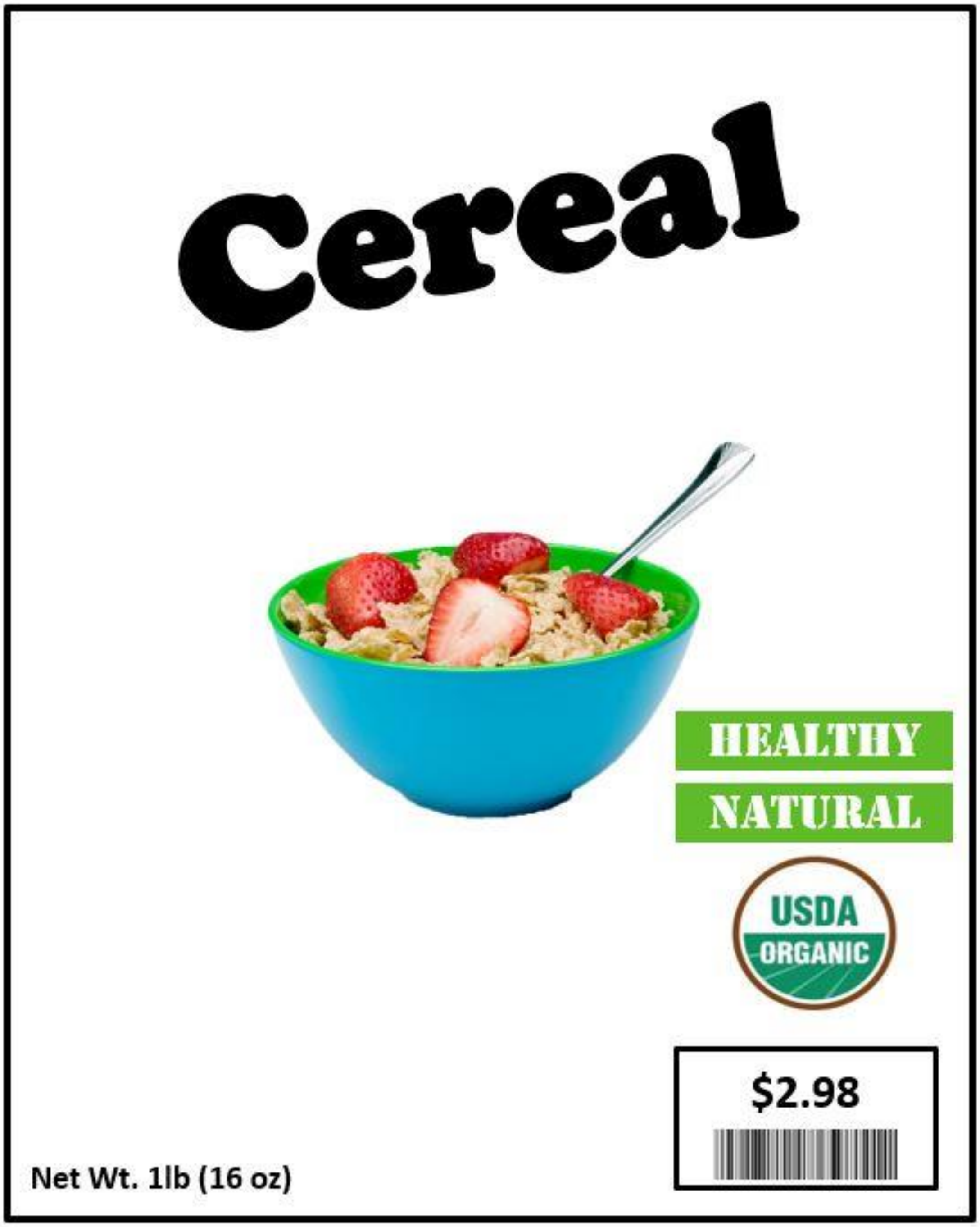

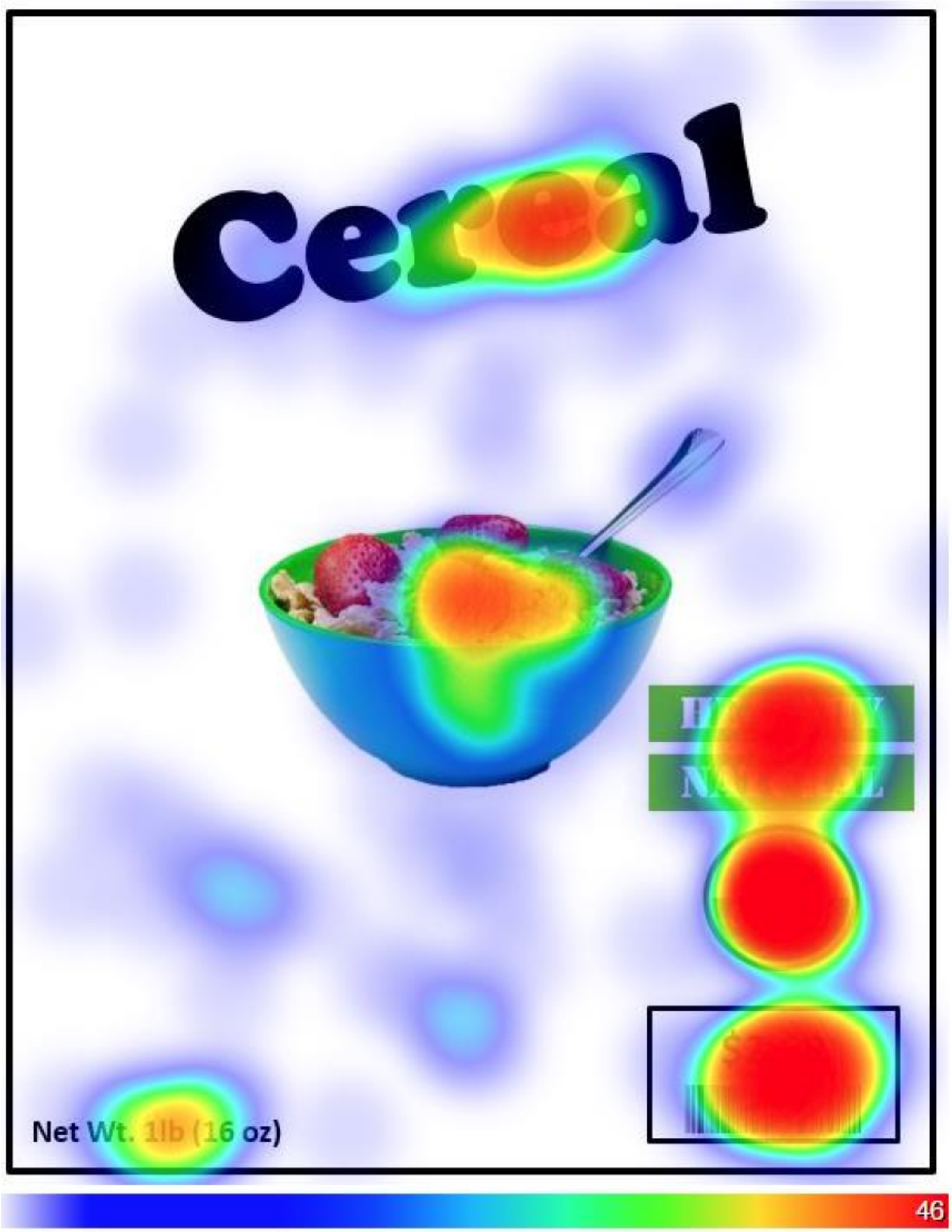

Q60 If you encountered this box of cereal in the grocery store, how likely would you be to purchase it? *(0 = no chance of buying; 100 = 100% chance of buying)*

End of Block: cereal9_combo_low

Start of Block: cereal10_combo_hi

Q61 Which part of the cereal box below is <u>most</u> attractive to you? *(please click or touch the image in the most attractive location)*

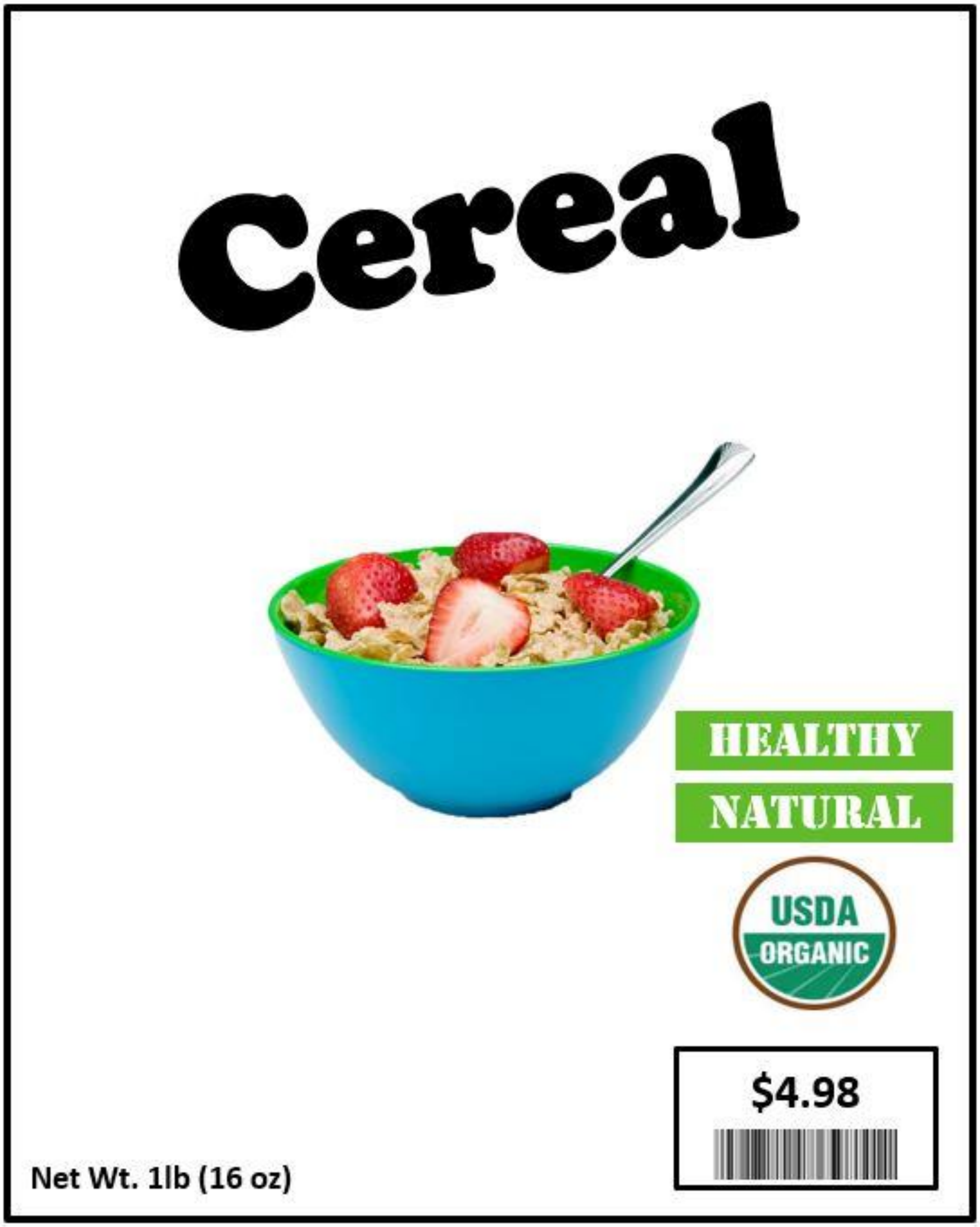

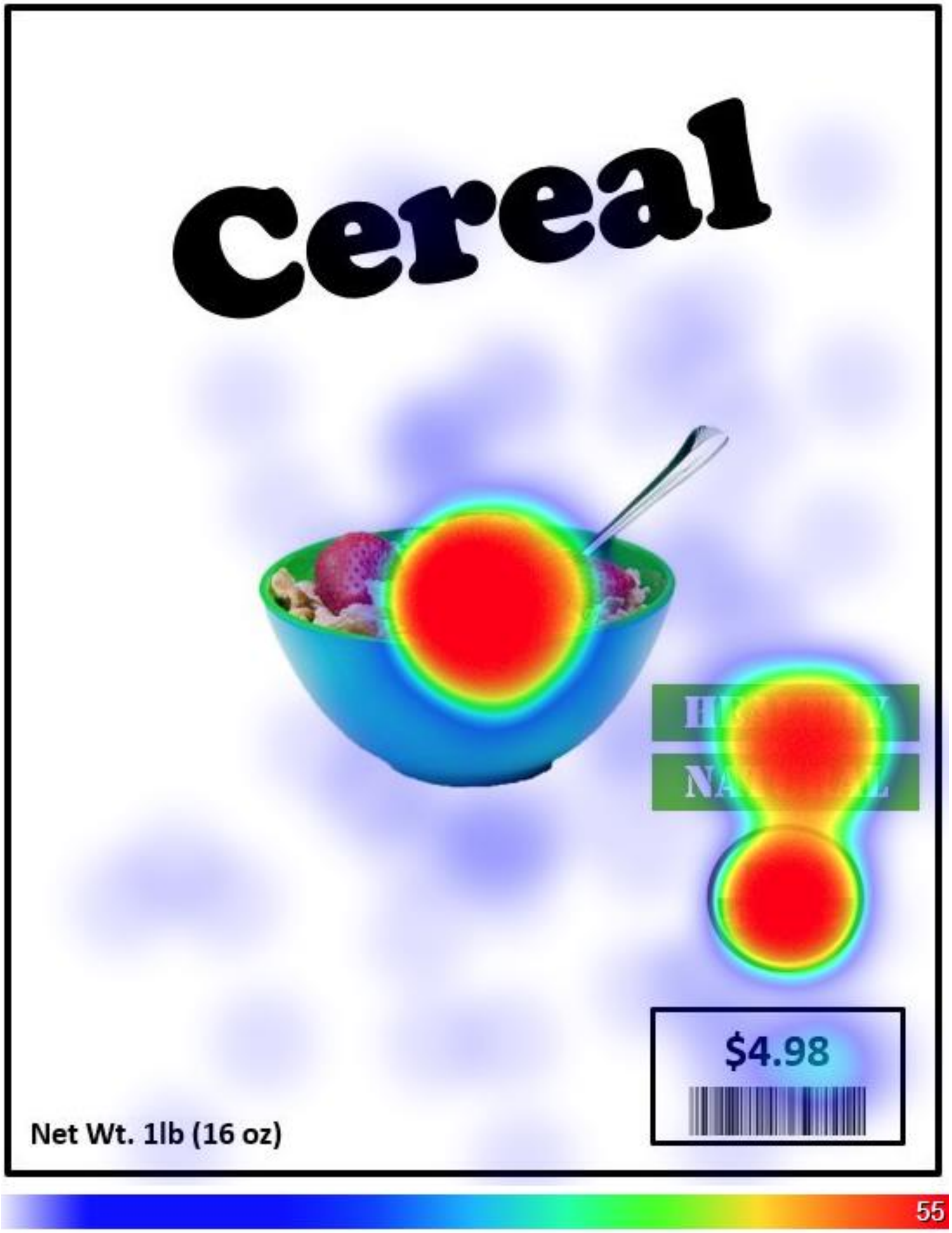
Cereal
$4.98
Net Wt. 1lb (16 oz)
55

Q62 Which part of the cereal box below is <u>least</u> attractive to you? *(please click or touch the image in the most attractive location)*

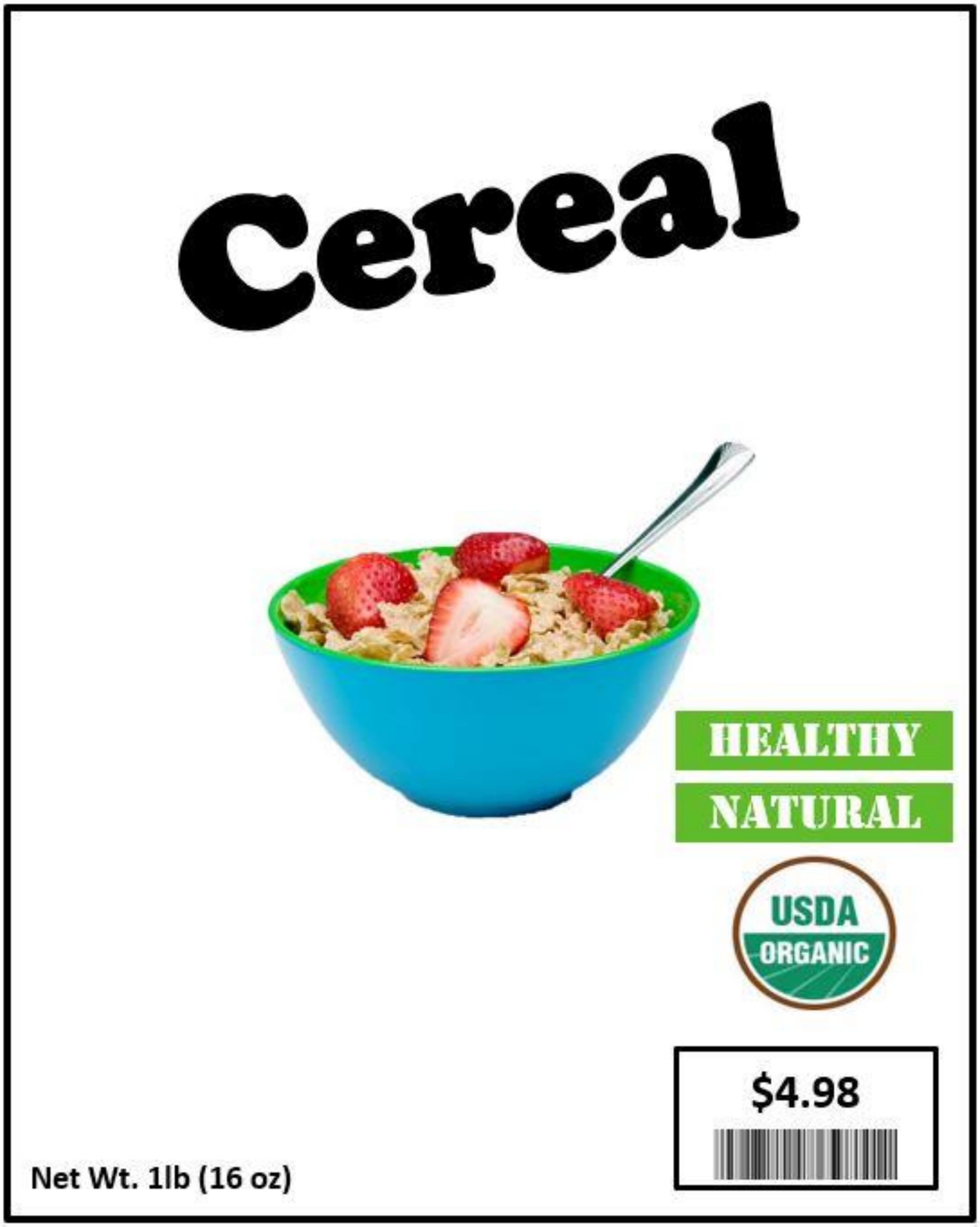

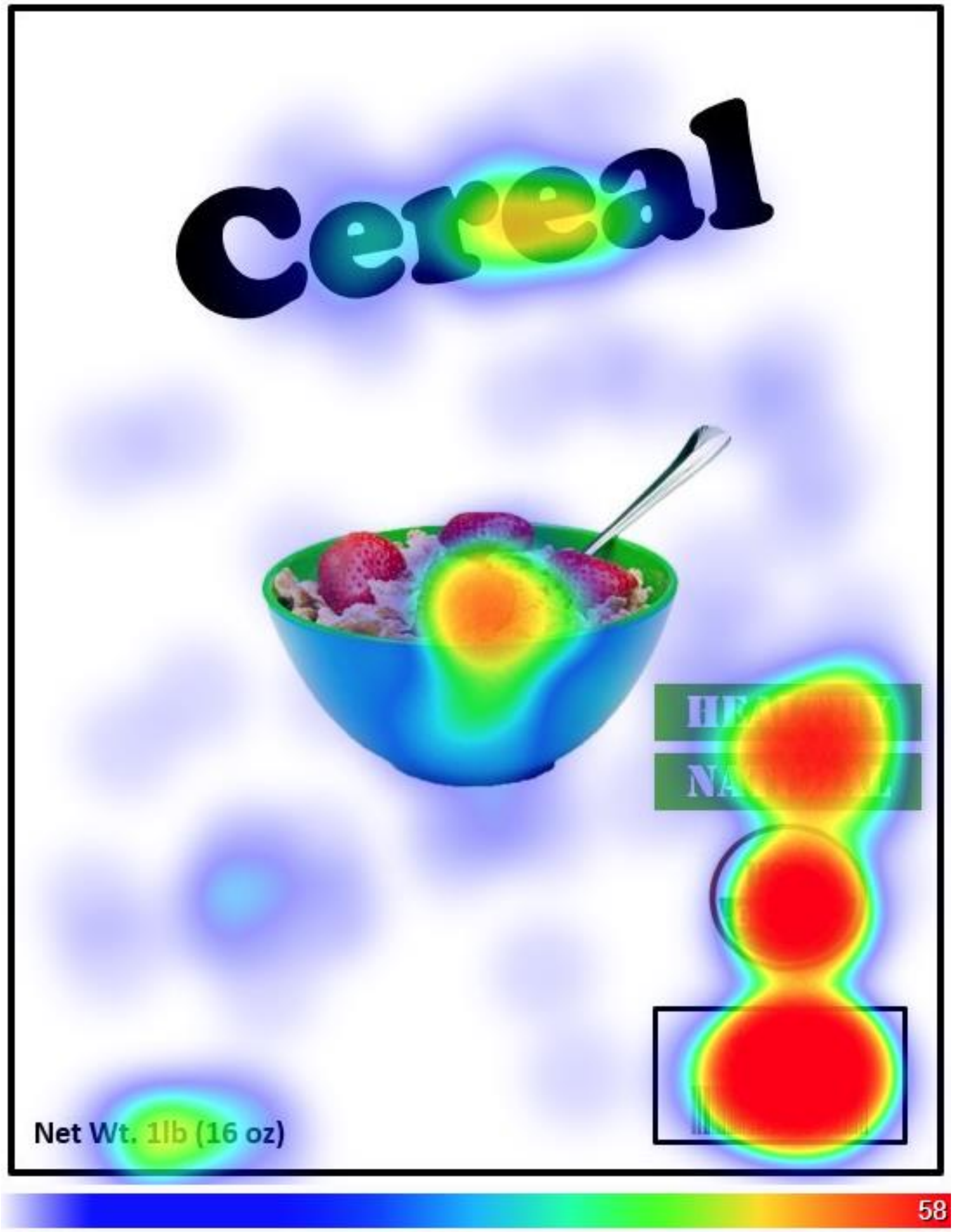

Q63 If you encountered this box of cereal in the grocery store, how likely would you be to purchase it? *(0 = no chance of buying; 100 = 100% chance of buying)*

End of Block: cereal10_combo_hi
