# Supplementary material for: Consumer beliefs about healthy foods and diets
Source: PLoS One. 2019 Oct 15;14(10):e0223098. doi: 10.1371/journal.pone.0223098 (PMC6793866; doi:10.1371/journal.pone.0223098)
Supplement: S1 Appendix — (Table A) Demographic Characteristics of Respondents. (Table B) Means and Correlations of Health Perceptions of 15 Foods. (Table C) Results of Factor Analysis Applied to Health Perceptions of 15 Foods: Rotated Factor Pattern (Standardized Regression Coefficients). (DOCX) [file pone.0223098.s002.docx]

# **Supplemental Appendix**

## **Table A**. Demographic Characteristics of Respondents

| **Demographic** | **Unweighted**  **(N=1,290)** | **Weighted**  **(N=1,290)** |
| --- | --- | --- |
| inc≤$19k | 13.2% | 16.9% |
| $20k≤inc≤$39k | 20.5% | 24.9% |
| $40k≤inc≤$59k | 16.7% | 17.5% |
| $60k≤inc≤$79k | 16.2% | 14.8% |
| $80k≤inc≤$99k | 9.9% | 8.6% |
| $100k≤inc≤$119k | 7.3% | 5.7% |
| $120k≤inc≤$139k | 5.3% | 4.1% |
| $140k≤inc≤$159k | 4.0% | 2.8% |
| $160k≤inc | 6.8% | 4.6% |
| 18≤age≤25 | 10.8% | 12.3% |
| 25≤age≤34 | 13.3% | 17.8% |
| 35≤age≤44 | 13.9% | 16.3% |
| 45≤age≤54 | 18.0% | 16.8% |
| 55≤age≤64 | 20.0% | 16.7% |
| 65≤age≤74 | 19.6% | 16.1% |
| 75≤age | 4.5% | 4.1% |
| HHsize=1 | 22.2% | 20.7% |
| HHsize=2 | 41.6% | 37.2% |
| HHsize=3 | 16.3% | 18.5% |
| HHsize=4 | 11.4% | 13.2% |
| HHsize=5 | 8.4% | 10.4% |
| HS edu | 18.2% | 36.7% |
| some college | 21.2% | 19.4% |
| associates | 12.2% | 11.4% |
| BS or BA | 28.6% | 17.9% |
| MS, MA, etc. | 13.3% | 8.4% |
| PhD, JD, etc | 4.8% | 3.1% |
| Female | 49.3% | 51.3% |
| White | 80.4% | 77.8% |
| Black | 10.1% | 11.9% |
| Other Race | 2.0% | 2.3% |
| Hispanic | 10.2% | 13.1% |
| Northeast | 19.0% | 17.2% |
| Midwest | 23.6% | 20.9% |
| South | 34.3% | 38.1% |
| West | 23.0% | 23.8% |
| Children under 12 in household | 21.7% | 26.5% |
| Vegetarian | 5.7% | 5.1% |
| SNAP participant | 11.4% | 14.8% |
| Democrat | 38.7% | 38.6% |
| Republican | 27.9% | 27.5% |
| Independent | 30.2% | 31.0% |
| Other Party | 3.2% | 3.0% |

**Table B**. Means and Correlations of Health Perceptions of 15 Foods

| **Food** | **Mean^a^** | **(1)** | **(2)** | **(3)** | **(4)** | **(5)** | **(6)** | **(7)** | **(8)** | **(9)** | **(10)** | **(11)** | **(12)** | **(13)** | **(14)** |
| --- | --- | --- | --- | --- | --- | --- | --- | --- | --- | --- | --- | --- | --- | --- | --- |
| Fresh fruit (1) | 1.05 | 1.00 |  |  |  |  |  |  |  |  |  |  |  |  |  |
| Frozen fruit (2) | 1.41 | 0.18 | 1.00 |  |  |  |  |  |  |  |  |  |  |  |  |
| Canned fruit (3) | 1.84 | -0.04 | 0.34 | 1.00 |  |  |  |  |  |  |  |  |  |  |  |
| Fresh vegetables (4) | 1.05 | 0.58 | 0.13 | 0.02 | 1.00 |  |  |  |  |  |  |  |  |  |  |
| Frozen vegetables (5) | 1.34 | 0.11 | 0.63 | 0.33 | 0.14 | 1.00 |  |  |  |  |  |  |  |  |  |
| Canned vegetables (6) | 1.72 | 0.01 | 0.31 | 0.67 | 0.04 | 0.35 | 1.00 |  |  |  |  |  |  |  |  |
| Beef (7) | 1.60 | 0.04 | 0.08 | 0.12 | 0.05 | 0.11 | 0.15 | 1.00 |  |  |  |  |  |  |  |
| Milk (8) | 1.37 | 0.19 | 0.10 | 0.20 | 0.12 | 0.09 | 0.17 | 0.33 | 1.00 |  |  |  |  |  |  |
| Eggs (9) | 1.23 | 0.20 | 0.15 | 0.08 | 0.17 | 0.15 | 0.12 | 0.39 | 0.36 | 1.00 |  |  |  |  |  |
| Chicken (10) | 1.24 | 0.22 | 0.08 | 0.10 | 0.20 | 0.14 | 0.15 | 0.43 | 0.36 | 0.46 | 1.00 |  |  |  |  |
| Bakery and cereal (11) | 2.10 | -0.04 | 0.07 | 0.30 | -0.04 | 0.09 | 0.20 | 0.18 | 0.24 | 0.09 | 0.05 | 1.00 |  |  |  |
| Vegetable oil (12) | 2.01 | 0.02 | 0.03 | 0.17 | 0.02 | 0.05 | 0.14 | 0.14 | 0.21 | 0.10 | 0.08 | 0.33 | 1.00 |  |  |
| Fish (13) | 1.14 | 0.31 | 0.08 | 0.03 | 0.34 | 0.06 | 0.07 | 0.25 | 0.29 | 0.36 | 0.51 | 0.02 | 0.04 | 1.00 |  |
| Candy (14) | 2.76 | -0.28 | 0.05 | 0.23 | -0.27 | 0.03 | 0.15 | 0.13 | 0.08 | -0.01 | -0.07 | 0.35 | 0.25 | -0.16 | 1.00 |
| Cheese (15) | 1.52 | 0.10 | 0.13 | 0.17 | 0.11 | 0.09 | 0.16 | 0.42 | 0.41 | 0.43 | 0.28 | 0.26 | 0.18 | 0.19 | 0.14 |

^a^1=healthy, 2=neither healthy nor unhealthy, 3=unhealthy

**Table C**. Results of Factor Analysis Applied to Health Perceptions of 15 Foods: Rotated Factor Pattern (Standardized Regression Coefficients)

| **Food** | **Factor1** | **Factor2** | **Factor3** |
| --- | --- | --- | --- |
| Fresh fruit | 0.153 | 0.114 | 0.599 |
| Frozen fruit | -0.100 | 0.691 | 0.190 |
| Canned fruit | 0.062 | 0.658 | -0.227 |
| Fresh vegetables | 0.136 | 0.136 | 0.580 |
| Frozen vegetables | -0.094 | 0.692 | 0.173 |
| Canned vegetables | 0.067 | 0.632 | -0.136 |
| Beef | 0.621 | -0.050 | -0.102 |
| Milk | 0.594 | 0.016 | -0.036 |
| Eggs | 0.615 | -0.023 | 0.125 |
| Chicken | 0.625 | -0.043 | 0.206 |
| Bakery and cereal items | 0.316 | 0.144 | -0.376 |
| Vegetable oil | 0.291 | 0.064 | -0.249 |
| Fish | 0.484 | -0.051 | 0.354 |
| Candy | 0.151 | 0.096 | -0.551 |
| Cheese | 0.613 | -0.002 | -0.124 |
